# Supplementary material for: XPRESSyourself: Enhancing, standardizing, and automating ribosome profiling computational analyses yields improved insight into data
Source: PLoS Comput Biol. 2020 Jan 31;16(1):e1007625. doi: 10.1371/journal.pcbi.1007625 (PMC7015430; doi:10.1371/journal.pcbi.1007625)
Supplement: S2 File — Archived version of the XPRESSpipe documentation. Current documentation can be found at https://xpresspipe.readthedocs.io/en/latest/. (PDF) [file pcbi.1007625.s002.pdf]

xpresspipe Documentation

Release 0.3.1

Jordan A. Berg

Oct 31, 2019

Contents

1 About **3**

2 Table of contents **5**
2.1 Overview 5
2.2 Quickstart 8
2.3 Beginner’s Guide 9
2.4 Installation 14
2.5 General Usage 16
2.6 Curating References 17
2.7 Single-End RNA-seq Pipeline 22
2.8 Paired-End RNA-seq Pipeline 24
2.9 Ribosome Profiling Pipeline 26
2.10 Quality Control 28
2.11 Analysis 33
2.12 Read Pre-Processing 36
2.13 Alignment 38
2.14 Read Quantification 41
2.15 Normalize 43
2.16 Other Features 44
2.17 FAQs 45
2.18 Updates 46

3 License **49**

4 Questions? **51**

CHAPTER 1

About

XPRESSpipe is a part of the XPRESSyourself suite of sequencing tools. XPRESSpipe is an automated, efficient, and flexible pipeline for end-to-end processing of ribosome profiling data.

XPRESSpipe is currently capable of handling single-end (SE), paired-end (PE), and ribosome profiling data.

If you have limited or no computational experience, please see our *Beginner's Guide*.

Please refer to the *Overview* page for more details regarding functionality.

For each sequencing type pipeline, all relevant quality control analyses are performed for that sequencing type, as well as gene coverage analysis of a housekeeping gene.

Other analyses can be performed by XPRESSplot. Please read the relevant documentation for more information.

CHAPTER 2

Table of contents

2.1 Overview

2.1.1 Ribosome Profiling

Ribosome profiling utilizes Next Generation Sequencing (NGS) to provide a detailed picture of the protein translation landscape within cells. Cells are lysed, translating ribosomes are isolated, and the ribosome protected mRNA fragments (ribosome footprints are integrated into a SE RNA-seq library. The library is then sequenced and processed similarly to a single-end RNA-seq run, with some exceptions:

- **5' and 3' ribosome footprint bias**: Footprint read pile-up at the 5' and 3' ends of transcripts is a well-documented phenomenon in ribosome profiling. This results from the kinetically slower translation initiation and termination steps, thus leading to higher density of ribosomes at these positions. Therefore, it is best to quantify reads by excluding the extremities of transcripts from consideration. The convention is to remove the first 45 nt and last 15 nt of each transcripts coding space. By using the `xpresspipe modifyGTF` or `xpresspipe curateReference` sub-modules with the flag `-t` provided, the user can prepare the required files for appropriate footprint quantification.

- **rRNA contamination**: Ribosome footprinting involves RNase digestion of a RNA. As these footprints are protected by the ribosome, they evade digestion and can later be incorporated into a sequence library. However, this leads to much of the ribosomal RNA being digested and fragments carrying through with the footprint samples. Commercial kits are often unable to target many of these randomly fragmented rRNA species, and it is thus advised to create depletion probes for dominant rRNA fragment species in ribosome profiling libraries for a given organism. By using the `xpresspipe rrnaProbe` sub-module, one can determine what the dominant consensus rRNA species are and create depletion probes to prevent their incorporation into future sequence libraries.

See this paper for a recent discussion and detailed protocol of the technique.

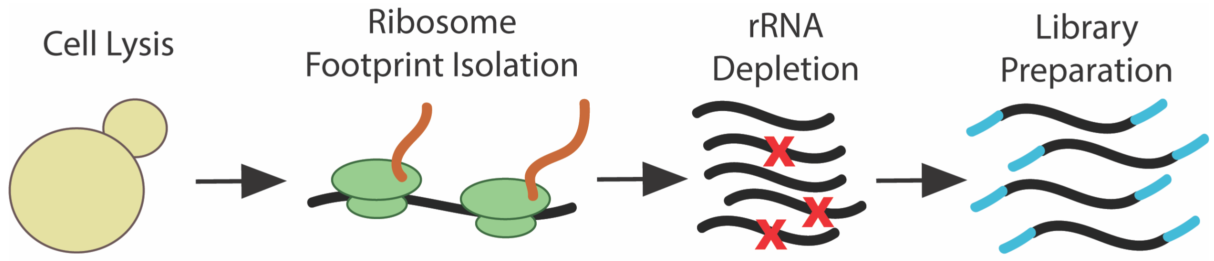

2.1.2 SE and PE RNA-seq

The XPRESSpipe pipeline is flexibly designed to be able to process and perform preliminary analyses on single-end (SE) or paired-end RNA-seq sequence read. Raw data is most often generated in the form of a .fastq or .txt file. This data is useful in determining the gene expression landscape of a population of cells. Other qualities, such as microRNA abundance, splice events, and sequence variants can also be detected and analyzed.

2.1.3 Software

XPRESSpipe aims to use the curate the most current and robust software packages required to process and analyze ribosome profiling and bulk RNA-sequencing. In designing XPRESSpipe, we referred to a variety of benchmarking studies to determine the best option for this pipeline. Below is a rationale for many of the packages chosen. As software continues to improve and benchmarking studies are published, XPRESSpipe and its documentation will be updated to reflect these improvements.

fastp – Read pre-processing
While external benchmarking has not been performed to our knowledge in recent years on read pre-processing tools, we chose to use fastp as it is fast, and (at least from self-reports) has reliable output. While most read pre-processing software does not diverge significantly in quality, we also favored fastp as it is able to handle more recent trends in RNA-Seq, such as trimming of unique molecular identifiers (UMIs).

STAR – Masking and Alignment
A recent benchmarking paper showed that STAR outperformed other comparable tools in speed and performance, increasing the number of correctly aligned reads, while reducing the number of falsely called reads as is the case with several other packages.

samtools/bedtools/deepTools – Alignment file post-processing
These tools handle the alignment file processing before quantification to identify PCR amplification artifacts (optional), remove non-uniquely aligned reads, and so on.

HTSeq – Read Quantification
HTSeq is used as it is a thoroughly vetting read counting package that has stood the test of time. Additionally, it is employed in the TCGA pipeline, thus we included this option to conform to these specifications. In the case of quantifying reads, HTSeq is very accurate and has the advantage of being able to quantify to specific gene features. This is particularly useful in ribosome profiling as it allows you to quantify reads to the protein coding space (CDS) of a transcript. Additionally, if one wanted to examine differences in uORF occupancy of ribosome footprints, they could specify `five_prime_utr` or `three_prime_utr` for the `--feature_type` option.

Cufflinks – Read quantification (Isoform abundance)

A recent benchmarking paper showed evidence that Cufflinks using default parameters performed the best compared to several other read quantification tools.

XPRESSpipe uses Cufflinks v2.1.1 as Cufflinks v2.2.1 appears to suffer from a persistent Seg Fault 11 error on MacOS. No significant changes effecting quantification have occurred between these versions. v2.1.1 is downloaded automatically for the user during installation of XPRESSpipe.

dupRadar – Library Complexity

dupRadar is a stable, easy to use tool for estimating library size complexity and doesn't suffer from systematic software issues like other tools that contain similar functionality.

SVA – Known Library Batch Correction

Used for correcting for known batch effects between samples (i.e. samples prepared on different days, by different people, etc.)

DESeq2 – Differential Expression Analysis

Perform differential expression analysis on the data.

MultiQC – Summary reports

MultiQC gathers log output from fastp, STAR, and HTSeq/Cufflinks to provide the user with a easy to view summary of their processed data at each step. A pipeline run will also FastQC

2.1.4 Methodology

We seek to provide the best methodology for high-throughput sequencing processing, and explain key components below.

Transcriptomic Reference Files

Read quantification often requires a transcriptome reference file in order to know what alignment coordinates map to what genes. We introduce a suite of GTF modification tools included in XPRESSpipe that we will briefly discuss:

- Isoforms: GTF files contain records for every isoform of a gene. However, since these isoforms all contain overlapping regions, many tools count a read mapping to one of these regions as a multi-mapper and either penalizes it or discards it completely. A common way to handle this is by taking only the longest transcript for each gene during quantification. This can be performed with `xpresspipe modifyGTF -l`.

- Protein Coding: When calculating mRNA expression levels, sample normalization to reduce technical bias from RNA-seq platforms is important. However, highly-abundant rRNAs can confound these metrics. Therefore, we provide an option to create a GTF file with only protein-coding annotated genes as input for quantification using `xpresspipe modifyGTF -p`.

- Ribosome Profiling Bias: During translation, there are three steps: 1) Initiation, 2) Elongation, and 3) Termination. There is usually a pause during Initiation and Termination, which will present itself as systematic spikes on the 5' and 3' ends of each transcript for ribosome profiling reads. A way to correct for the kinetics of initiation and termination and measure translational capacity itself is to avoid mapping reads to the first 15 codons and last 5 codons of a transcript. `xpresspipe modifyGTF -t` handles this by searching the exon space of each transcript and pruning the given amounts off of each so that these regions are considered non-coding space. This process is performed recursively, so that if you were trimming 45 nt from the 5' end and exon 1 was only 30 nt, exon 1 would be removed and exon 2 would be trimmed by 15 nt.

PCR De-Duplication

During sequence library creation, a PCR amplification step is common in order to produce enough sequence material, but often, different reads are amplified differentially. When UMIs are not used, these duplication events can lead to artificially higher expression of a transcript. We therefore include an optional PCR de-duplication step for experiments not using UMIs. Be warned, this can introduce additional biases and should be used with caution. Performing library complexity analysis on the samples should indicate whether or not computational de-duplication should be performed. If UMIs were used, these can be specified and will be handled by the pipeline.

Meta-Analysis

- Read distribution: Once reads are trimmed of low quality bases or adapter sequences, looking at the distribution of read lengths can be helpful in identifying that the expected RNA was incorporated into the library. This is especially useful in ribosome profiling datasets, where ideally all reads isolated and incorporated into the library should fall within the 21-33 nt range.

- Metagene: Metagene analysis takes the read coverage across all transcripts in a sample and compiles their distribution along a representative transcript. This is useful in identifying any systematic 5' or 3' biases in the library preparation step.

- Periodicity: A helpful metric of ribosome profiling libraries is looking at the characteristic 3 nt/1 codon stepping of the translating ribosome.

- Gene Coverage: Aspects of a transcript's read coverage or occupancy can be of interest. However, other genome browsers like IGV retain introns, and in the case of transcripts with massive introns, the actually coding space will be difficult to analyze succinctly. XPRESSpipe will plot the gene coverage across an exon-only transcript representation. However, it may still be worthwhile to explore intron coverage in some instances.

2.2 Quickstart

2.2.1 Running XPRESSpipe

Along with the video walkthroughs provided below, we recommend users start with the `build` module after installation:

```
$ xpresspipe build
```

This will present the user with a series of questions to help design the command that should be executed in the command line to curate a reference or to run the pipeline

2.2.2 Video Walkthroughs

The following is a short tutorial showing you how to install XPRESSpipe:

Reference building

Running XPRESSpipe on sequence data

If any of these are going too slow for you, check them out in the README

2.3 Beginner's Guide

2.3.1 First Steps

If this is your first time doing any programming, congratulations! You are embarking upon a very rewarding path. As with learning any new spoken language, there is a learning curve associated with learning a computer language. While XPRESSpipe is aimed at reducing the majority, if not (hopefully) all of the overhead associated with processing this data, using this software will still require some effort, just as would learning any new language or laboratory technique.

XPRESSpipe is run through the command line interface (or CLI). This may seem daunting, but luckily, several free online courses are available to quickly catch you up to speed on some of the basics that will be required to use this software. We recommend Codecademy's CLI course, which you can find here and should take only a couple of hours (Codecademy estimates ~10 hours, but you probably don't need to finish the course to be ready to use XPRESSpipe. The purpose of this is to help you become more comfortable with the command line). We recommend watching the walkthrough videos found on the *quickstart* page.

Once you're ready to jump into the command line, we can get rolling! For the steps below, we're going to assume we are on an Mac operating system and provide examples under this pretext, but this software is compatible with any Linux-like operating system and the syntax is largely the same (sorry Windows users! – but if you have a newer version of Windows, you may be able to use a Linux-flavored environment).

2.3.2 Install XPRESSpipe

- Please refer to the *installation documentation* or the walkthrough video below:

2.3.3 Generate Reference Files

- Before we can process our raw RNA-seq data, we need to create a reference directory (or for a folder, in other terms). In this example, we will be working with human-derived RNA-seq data, so let's perform the following in the command line:

```
$ cd ~/Desktop
$ mkdir reference_folder
$ mkdir reference_folder/fasta_files
```

1. The first command helped us navigate to the Desktop. The "~" icon is a shortcut for the User directory, and every directory needs to be separated by a "/"
2. The second command created a new folder in the Desktop directory called `reference_folder`
3. The third command created a new folder in the reference directory for intermediate reference files

- Now let's get the reference files. We're going to do this directly in the command line, but if you have trouble with this, I will explain an alternative afterwards. Quick note, because the next lines of code are a bit long, I used the "" character to indicate I am continuing the command in the next line. You not include these characters when executing the command, they just help make the code more readable. We will first read the retrieval commands into a file which will additionally act as a log file for the version for the genome version we are using.
- You should modify the the variable calls between the # signs. For `GTF_URL`, you should change the URL currently provided to the one appropriate for your organism of interest. Make sure you are downloading the GTF file and NOT the GFF file. For `FASTA_URL`, you should do the same as before with the URL to the chromosome DNA FASTA files, but you should only copy the URL up to "chromosome", but not include the chromosome identifier. For `CHROMOSOMES`, type out the chromosome identifiers you want to download between the " characters with a space between each.

```
$ cd reference_folder/

### Change these ###
$ echo 'GTF_URL=ftp://ftp.ensembl.org/pub/release-97/gtf/homo_sapiens/Homo_sapiens.
↪GRCh38.97.gtf.gz' >> fetch.sh
$ echo 'FASTA_URL=ftp://ftp.ensembl.org/pub/release-97/fasta/homo_sapiens/dna/Homo_
↪sapiens.GRCh38.dna.chromosome' >> fetch.sh
$ echo 'CHROMOSOMES="1 2 3 4 5 6 7 8 9 10 11 12 13 14 15 16 17 18 19 20 21 22 X Y"'
####################

$ echo 'curl -O $GTF_URL' >> fetch.sh
$ echo 'gzip -d Homo_sapiens.GRCh38.97.gtf.gz' >> fetch.sh
$ echo 'mv Homo_sapiens.GRCh38.97.gtf transcripts.gtf' >> fetch.sh
$ echo 'cd fasta_files/' >> fetch.sh
$ echo 'for X in $CHROMOSOMES; ' >> fetch.sh
$ echo 'do curl -O ftp://ftp.ensembl.org/pub/release-97/fasta/homo_sapiens/dna/Homo_
↪sapiens.GRCh38.dna.chromosome.${X}.fa.gz; done ' >> fetch.sh
$ echo 'gzip -d *.gz' >> fetch.sh
$ echo 'cd ../' >> fetch.sh
$ bash fetch.sh
```

- Let's discuss what we just did:
1. We navigated into the reference folder, downloaded a GTF reference file and unzipped it, then navigated to the `fasta_file` directory to download the raw reference data and unzipped it. Finally, we returned to the main reference directory.
2. If this didn't work, we can navigate to Ensembl to download the relevant data. We need to get the GTF and DNA chromosomal FASTA files for our organism of interest. The link to the chromosome sequence files actually contains more files than we need. We just need the files that start with `Homo_sapiens.GRCh38.dna.chromosome`. You can download them, move them to the appropriate directories within your reference directory, and unzip the files by double-clicking on them.

- Now we need to curate these references files into something the sequencing alignment software can use. Since we are using ribosome profiling data, we want a reference that will allow us to avoid mapping to the 5' and 3' ends of

genes. We also don't want to align to anything but protein coding genes. Finally, we want to quantify to the longest transcript. This last bit just helps the software avoid confusion when a gene has multiple splice variants to choose from. Since this is short read sequencing (let's say we were doing 50 bp single-end sequencing), we also want to factor this into the curation of the reference (see the `--sjdbOverhang` argument below).

```
$ xpresspipe curateReference \
              --output ./ \
              --fasta fasta_files/ \
              --gtf ./transcripts.gtf \
              --protein_coding \
              --truncate \
              --sjdbOverhang 49

### or ###

$ xpresspipe build

### And then choose the curate option ###
```

- The truncation option is only necessary when using XPRESSpipe to process ribosome profiling samples and their associated RNA-seq samples.
- If interested in quantifying miRNA, etc, leave out the `--protein_coding` argument.
- If running sequencing where the read (single-end) or mates not equal to 100 bp, you will want to change the `--sjdbOverhang` argument to be the length of one of the paired-end reads - 1, so if we ran 2x100bp sequencing, we would specify `--sjdbOverhang 99` (although in this case, the default of `--sjdbOverhang 100` is just fine). If you changed this number, remember this for the next steps as you will need to provide it again if changed here.
- This may take awhile, and as we will discuss later, you may want to run these steps on a supercomputer, but this will serve as a preliminary guide for now.
- One final consideration – if we are dealing with an organism with a smaller genome size, we will want to provide the `--genome_size` parameter with the the number of nucleotides in the organism's genome. If you change this parameter in this step, you will need to provide the parameter and value in the `align`, `riboseq`, `seRNAseq`, and `seRNAseq` modules.

2.3.4 Process Raw Sequencing Files

- Now let's get our raw data::
1. Make a new folder, something called `raw_data` or whatever you like and place your data there.
2. Make sure the files follow proper naming conventions (see naming conventions at *General Usage*)
3. Now let's process the data
4. Let's also create a folder called something like `output`
5. Also, make sure you have the 3' adapter sequence handy used when generating your sequencing library
6. We'll feed the program the new GTF file that contains only longest transcript, protein coding, truncated references generating in the reference curation step
7. We'll give the experiment a name and also specify what method of sample normalization we want performed on the count data
8. We also need to specify the `--sjdbOverhang` amount we fed into the reference curation step, so in this case we will use `--sjdbOverhang 49`

```
$ xpresspipe riboseq --input raw_data/ \
                    --output output/ \
                    --reference reference_folder/ \
                    --gtf reference_folder/transcripts_LCT.gtf
                    --experiment riboseq_test
                    --adapter CTGTAGGCACCATCAAT
                    --method RPKM
                    --sjdbOverhang 49

### or ###

$ xpresspipe build

### And then choose the appropriate pipeline to build
```

- If you are running a lot of files, especially for human samples, this may take a lot of time. We recommend running this on some kind of server. A situation like yeast with few samples may be feasible to run on a personal computer, but will likely also take some time.

Sequencing Metrics

In your output folder, you will see a file named `riboseq_test_multiqc_report.html`. This file will compile the statistics from each processing step of the pipeline for each sample file you provided as input. Things like read quality, mapping, and quantification statistics can be found here. Just double-click the file or execute the following command to open in your default browser window.

```
$ open riboseq_test_multiqc_report.html
```

Library Complexity

Within the `complexity` directory in your output folder, you will find summary PDFs for all samples processed analyzing library complexity of each sample.

Metagene Analysis

Within the `metagene` directory in your output folder, you will find summary PDFs for all samples processed analyzing the metagene profile of each sample.

Periodicity (Ribosome Profiling)

Within the `periodicity` directory in your output folder, you will find summary PDFs for all samples processed analyzing ribosome periodicity of each of each sample containing reads 28-30nt.

Count Data and Downstream Analysis

Within the `counts` directory in your output folder, you will find individual counts tables for each sample, as well as compiled tables for each sample that was processed.

2.3.5 Supercomputing

Install

- Much of the same commands will be performed as above, aside from a couple exceptions:
1. When installing XPRESSpipe, you need to provide a location for personal storage of the software:

```
$ python setup.py install --prefix ~/.local/bin
```

2. Add this path to your `$PATH`:

```
$ echo 'export PATH="~/.local/bin:$PATH"' >> ~/.bash_profile
$ echo 'export PYTHONPATH="/uufs/chpc.utah.edu/common/home/u0690617/.local/bin/lib/
↪python3.7/site-packages"' >> ~/.bash_profile
```

3. Let's test this to make sure everything is operating properly:

```
$ cd ~/
$ xpresspipe test
```

Run Data

- The commands here are the same as above, but likely the method of execution will be different. A lot of supercomputing clusters manage job submission through a system called SLURM. Each supercomputing cluster should have individualized and tailored instructions for proper usage. We will briefly provide an example of how one would submit a job to a SLURM batch system:

```
#!/bin/bash
#SBATCH --time=72:00:00
#SBATCH --nodes=1
#SBATCH -o /scratch/general/lustre/$USER/slurmjob-%j
#SBATCH --partition=this_cluster_has_no_name

#set up the temporary directory
SCRDIR=/scratch/general/lustre/$USER/$SLURM_JOBID
mkdir -p $SCRDIR

# Provide location of raw data and parent reference directory
SRA=/scratch/general/lustre/$USER/files/your_favorite_experiment_goes_here
REF=/scratch/general/lustre/$USER/references/fantastic_creature_reference

# Send raw data to your Scratch directory
mkdir $SCRDIR/input
cp $SRA/*.fastq $SCRDIR/input/.
```

(continues on next page)

(continued from previous page)

```
# Make an output directory
mkdir $SCRDIR/output
cd $SCRDIR/.

xpresspipe riboseq -i $SCRDIR/input -o $SCRDIR/output/ -r $REF --gtf $REF/transcripts_
↪CT.gtf -e this_is_a_test -a CTGTAGGCACCATCAAT --sjdbOverhang
```

- To queue this script into the job pool, you would do the following:

```
$ sbatch my_batch_script.sh
```

- To monitor the progress of your job, execute the following:

```
$ watch -n1 squeue -u $USER
```

- After the job is finished, you can export the data as shown in the next section.

Explore the Data

Once the data is finished processing, we can start exploring the output. Explanations each quality control analysis can be found in the *Analysis* section of the documentation.
In order to get the data from a HPC to your personal computer, you can use a command like the following:

```
$ cd ~/Desktop # Or any other location where you want to store and analyze the data
$ scp USERNAME@this_cluster_has_no_name.chpc.university.edu:/full/path/to/files/file_
↪name.suffix ./
```

2.4 Installation

2.4.1 Install XPRESSpipe

- Let's enter the command line.
1. Click on the Finder icon the top right side of the screen on your Mac (or wherever else it might be located)
2. Type "Terminal" into the search bar and click on the app icon

- Great! Now we are in the command line interface. As a review, anything followed by a "$" in the command line is a command and you can execute each command by pressing Enter after typing. You can also auto-complete file names using Tab. But be careful, **space and characters must be typed exactly and commands are case-sensitive**.
- First, let's install conda, a package manager that will help us download all required dependencies.

```
# If on a MacOS
$ curl -O https://repo.anaconda.com/miniconda/Miniconda3-latest-MacOSX-x86_64.sh

# If on a LinuxOS
$ curl -O https://repo.anaconda.com/miniconda/Miniconda3-latest-Linux-x86_64.sh
$ bash ~/Miniconda3-latest-MacOSX-x86_64.sh

# Enter yes for all successive prompts and allow the script to install Conda into␣
↪your path
# After installation, the install script can be removed
rm ~/Miniconda3-latest-MacOSX-x86_64.sh
```

- Let's get the latest version of XPRESSpipe by executing the lines of code in the code block below. Replace the URL for the version of XPRESSpipe for whatever version you want (these can be found under the `releases` tab on the XPRESSpipe GitHub repository).

```
$ cd ~
$ curl -L -O https://github.com/XPRESSyourself/XPRESSpipe/archive/v0.2.1b0.tar.gz
$ tar xvzf v0.2.1b0.tar.gz
$ cd XPRESSpipe-0.2.1b0
```

- Now we can give Conda the dependency file to process for us from XPRESSpipe:

```
$ conda env create --name xpresspipe -f requirements.yml
# conda activate xpresspipe
```

- This installation method will create a separate environment for XPRESSpipe and all its dependencies to live in. Each time you open the command line, you will need to type `conda activate xpresspipe` to use XPRESSpipe
- Let's install XPRESSpipe and test that the installation was successful by executing the following:

```
$ python setup.py install
$ xpresspipe test
```

- If a summary menu appeared in the command line interface, it means we are good to go! Congrats! You are almost ready to use XPRESSpipe!
- You can run `xpresspipe --help` to see a list of the available modules within XPRESSpipe. To see specific parameters for a module, type `xpresspipe <module_name> --help`.

2.4.2 Docker Container

NOTE: The Docker containerization is still under development.

XPRESSpipe is available as a fully independent Docker container. By downloading the Docker software and using the below command, a image of XPRESSpipe and all associated dependencies localized to a single file for ease of use.

1. Download XPRESSpipe Docker image:

```
$ docker image pull jordanberg/xpresspipe:latest
```

2. Run XPRESSpipe:

```
$ docker run jordanberg/xpresspipe --help
```

If the help menu prints, XPRESSpipe if functioning properly and you can replace the `--help` option with the appropriate sub-module and arguments.

2.5 General Usage

XPRESSpipe can be run essentially from beginning to end as a pipeline, or as individual sub-modules. We will describe each option in more detail in each section of the documentation. The purpose of XPRESSpipe is to automate the alignment, quality control, and initial analysis of single-end (SE), paired-end (PE), and ribosome profiling data. It is intended that input data is in its own directory and that each file is a properly formatted `fastq` file. However, the suffix for these files can be `fq` or `txt` as well. They can be zipped (`zip` or `gz`) or unzipped. When using intermediate sub-modules, such as `align` or `readDistribution`, input will vary and is explicated

in the `--help` menu for each sub-module.

Further analysis on the resulting datasets can be performed using XPRESSplot.

2.5.1 File Naming

In order for many of the XPRESSpipe functions to perform properly and for the output to be reliable after alignment (except for generation of a raw counts table), file naming conventions must be followed.

1. Download your raw sequence data and place in a folder – this folder should contain all the sequence data and nothing else.

2. If you are working with single-end data, the files must be a FASTQ-formatted file and end with the suffix `fastq`, `fastq.gz`, `fq`, `fq.gz`, `txt`, `txt.gz`. We recommend the `fastq` or `fastq.gz` suffix.

3. If you are working with paired-end data, the rules from `Step 2` apply, but must the suffix must be prefaced by the paired read group number as below:

or

2.5.2 Data Output

Running `seRNAseq`, `peRNAseq`, or `riboseq` will output all intermediate and final data files as shown in this schematic:

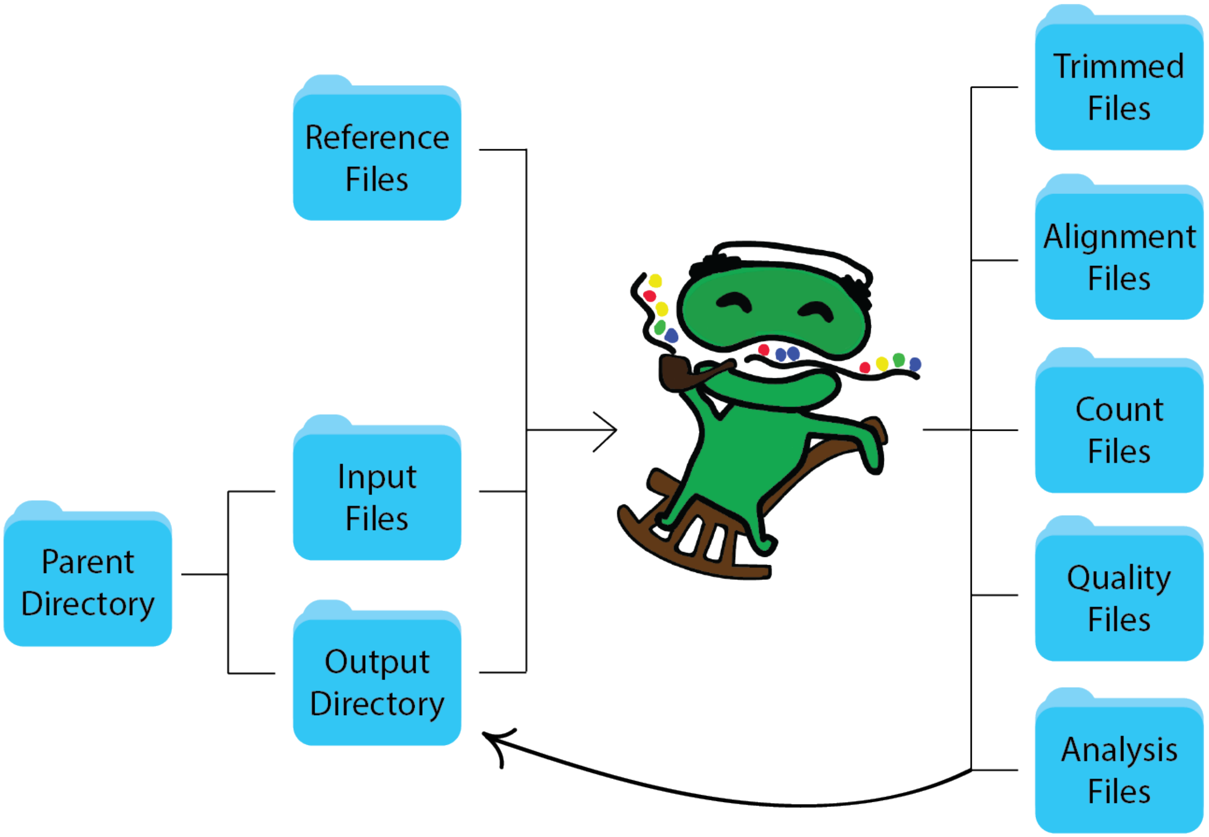

2.6 Curating References

In order to quantify transcription levels from RNA-Seq data, reads must be mapped to a reference genome or transcriptome. While there are multiple alignment software packages available, XPRESSpipe performs this step using a current version of STAR for several reasons:

- **Splice Junction Aware**: STAR is capable of mapping reads spanning a splice junction, where more traditional packages, such as Bowtie, are incapable of doing so and are better suited for tasks such as genome alignment.

- **Performance**: While computationally greedy (a human genome alignment requires upwards of 30 Gb RAM), the performance and accuracy is excellent compared to the majority of other splice-aware aligners currently available

- **Standard**: The foundation of the pipeline used in XPRESSpipe is based in the TCGA standards for RNA-Seq alignment. This method utilizes a guided or 2-pass alignment program. In the guided alignment, a GTF with annotated splice junctions is used to guide the alignments over splice juntions. In the 2-pass alignment, reads are mapped across the genome to identify novel splice junctions. These new annotations are then incorporated into the reference index and reads are re-aligned with this new reference. While more time-intensive, this step can aid in aligning across these junctions, especially in organisms where the transcriptome is not as well annotated. If mapping to a well-documented organism, this step can be forgone and STAR will use the GTF annotations to determine intronic regions of transcripts for read mapping.

2.6.1 XPRESSpipe Reference Requirements

An XPRESSpipe compatible reference directory must meet some requirements:
- All chromosomal genome fasta files are in their own directory within the parent reference directory. If a FASTA file with all chromosomes combined is available for your organism, this can be provided, but must be in its own directory.
- A sub-directory, named `genome`, contains the STAR reference files. If `createReference` is used to curate the reference, and the parent reference directory was provided as output location, this directory creation and file formatting will be handled automatically by XPRESSpipe.
- A transcript reference (GTF), is located in the reference parent directory and is named `transcripts.gtf`. If a coding-only or truncated reference GTFs are desired for read quantification, these should also be in this directory (`truncate` will handle file naming and formatting so long as the output location is specified as this parent directory). This file will then need to be specified within an XPRESSpipe pipeline.
A completed reference directory can be created that follows these requirements by creating a directory, placing the transcripts.gtf and genomic chromosome fasta files in the parent directory and running :data:'curateReference' as described below

2.6.2 Get Sequence Files

The following is an example of how to get the reference files needed for generating a human reference:

```
$ mkdir human_reference
$ mkdir human_reference/genome_fasta
$ cd human_reference/
$ curl ftp://ftp.ensembl.org/pub/release-95/gtf/homo_sapiens/Homo_sapiens.GRCh38.95.
↪gtf.gz -o transcripts.gtf.gz
$ for i in 1 2 3 4 5 6 7 8 9 10 11 12 13 14 15 16 17 18 19 20 21 22 X Y; do curl -O␣
↪ftp://ftp.ensembl.org/pub/release-95/fasta/homo_sapiens/dna/Homo_sapiens.GRCh38.dna.
↪chromosome.${i}.fa.gz; done
$ gzip -d *.gz
$ mv *fasta genome_fasta
```

- The chromosome IDs may vary depending on your organism.

2.6.3 Perform Full Reference Curation

The following will create a XPRESSpipe-formatted reference directory containing all STAR reference files and transcript references needs for quantification and meta-analysis.
A parent reference directory containing the transcripts.gtf file and all chromosomal genome fasta files must be present.
More details as to what each specific parameter is doing can be found in the sections below.

Arguments

The help menu can be accessed by calling the following from the command line:

```
$ xpresspipe curateReference --help
```

Required Arguments	Description
`-o <path>, --output <path>`	Path to output directory
`-f <path>, --fasta <path>`	Path to genome fasta files (file names should end in .fa, .fasta, or .txt and no other files should exist in the directory with similar extensions)
`-g </path/transcripts.gtf>, --gtf </path/transcripts.gtf>`	Path and file name to transcript reference file names 'transcripts.gtf'

Optional Arguments	Description
`-l, --longest_transcript`	Provide argument to keep only longest transcript per gene record (RECOMMENDED)
`-p, --protein_coding`	Provide argument to keep only gene records annotated as protein coding genes
`-t, --truncate`	Provide argument to truncate gene records
`--truncate_5prime`	Amount to truncate from 5' end of each transcript, requires –truncate argument (default: 45)
`--truncate_3prime`	Amount to truncate from 3' end of each transcript, requires –truncate argument (default: 15)
`--sjdbOverhang <value>`	Specify length of genomic sequences for constructing splice-aware reference. Ideal length is `read length - 1`, so for 2x100bp paired-end reads, you would use 100 - 1 = 99. However, the default value of `100` should work in most cases
`--genome_size <int>`	If mapping to an organism with a small genome, provide the length in nucleotides. If you are not sure your organism has a small genome, provide the number of bases and XPRESSpipe will decide if this parameter needs to be changed during runtime
`-m`	Number of max processors to use for tasks (default: No limit)

Examples

Example 1 – Create XPRESSpipe-formatted reference for single-end alignment:
- Creates a star reference for single-end read mapping (1x50bp reads)
- Keeps the longest transcript for each gene record
- Keeps only protein_coding annotated transcripts
- Truncates the first 45 nucleotides from the first exon of every transcript (default)
- Truncates the last 15 nucleotides from the last exon of every transcript (default)

```
$ xpresspipe curateReference -o /path/to/se/ref/ -f /path/to/se/ref/ -g /path/to/se/
↪ref/transcripts.gtf --longest_transcript --protein_coding --truncate --sjdbOverhang␣
↪49
```

Example 2 – Create refFlat files:
- Creates a star reference for paired-end read mapping (2x100bp reads)
- No modifications are made to the GTF file
- Processes are limited to 10 cores

```
$ xpresspipe curateReference -o /path/to/pe/ref/ -f /path/to/pe/ref/ -g /path/to/pe/
↪ref/transcripts.gtf -m 10
```

2.6.4 STAR Reference Curation

The following creates a STAR reference compatible with XPRESSpipe. These files are output in a directory created during curation called `genome` in the specified `--output` directory.

Arguments

The help menu can be accessed by calling the following from the command line:

```
$ xpresspipe makeReference --help
```

Required Arguments	Description
`-o <path>, --output <path>`	Path to output directory
`-f <path>, --fasta <path>`	Path to genome fasta files (file names should end in .fa, .fasta, or .txt and no other files should exist in the directory with similar extensions)
`-g </path/transcripts.gtf>, --gtf </path/transcripts.gtf>`	Path and file name to transcript reference file names 'transcripts.gtf (DO NOT USE MODIFIED GTF HERE)'

Optional Arguments	Description
`--sjdbOverhang <int>`	Specify length of genomic sequences for constructing splice-aware reference. Ideal length is `read length - 1`, so for 2x100bp paired-end reads, you would use 100 - 1 = 99. However, the default value of `100` should work in most cases
`--genome_size <int>`	If mapping to an organism with a small genome, provide the length in nucleotides. If you are not sure your organism has a small genome, provide the number of bases and XPRESSpipe will decide if this parameter needs to be changed during runtime
`-m`	Number of max processors to use for tasks (default: No limit)

Examples

Example 1 – Create a single-end sequencing reference:

- Paths to output and location of genome fasta files for each chromosome are provided, as well as path and file name to transcripts.gtf file
- Default number of threads are used for preparing reference

```
$ xpresspipe makeReference -o /path/to/reference/ -f /path/to/reference/ -g /path/to/
↪reference/transcripts.gtf --sjdbOverhang 49
```

Example 2 – Create a paired-end sequencing reference:

- 12 threads are specified for reference creation
- The as 2x100bp paired-end sequencing was used, the default value for `--sjdbOverhang` of `100` is appropriate in this case

```
$ xpresspipe makeReference -o /path/to/reference/ -f /path/to/reference/ -g /path/to/
↪reference/transcripts.gtf -t 12
```

Example 3 – Create a single-end sequencing reference for Saccharomyces cerevisiae:
- Paths to output and location of genome fasta files for each chromosome are provided, as well as path and file name to transcripts.gtf file
- Default number of threads are used for preparing reference
- Genome size is specified

```
$ xpresspipe makeReference -o /path/to/reference/ -f /path/to/reference/ -g /path/to/
↪reference/transcripts.gtf --sjdbOverhang 49 --genome_size 3000000
```

2.6.5 Transcript Reference Modification

At times, quantification of transcripts to a modified transcript reference is desirable. Below are some examples:
1. As ribosomal RNA (rRNA) contamination is common in RNA-seq, even when a depletion step was performed prior to library preparation, it is sometimes desirable to not count these and other non-coding RNAs in the quantification and analysis.
2. During ribosome profiling library preparation, where a 5' and 3' pile-up of ribosome footprints due to slow initation and termination kinetics of footprints is common, it is suggested to exclude the first 45-50 nucleotides from the 5' end and 15 nucleotides from the 3' end of each transcript during quantification. This command will automatically curate an Ensembl GTF to meet these demands for read quantification.
3. Several genes encode multiple isoforms or transcripts. During quantification, many software packages for counting reads to genes consider a read mapping to multiple transcripts of the same gene as a multi-mapper. Unless interested in alternate isoform usage, it is recommended that transcriptome reference files only contain the longest transcript for each gene.

The `modifyGTF` sub-module provides the ability to make the above-mentioned modifications to a GTF transcriptome reference file. The modified GTF file is output at the end and the filename is labeled with the modifications made. Truncations to each transcript reference are stranded-aware.

Arguments

The help menu can be accessed by calling the following from the command line:

```
$ xpresspipe modifyGTF --help
```

Required Arguments	Description
`-g </path/transcripts.gtf>, --gtf </path/transcripts.gtf>`	Path and file name to reference GTF

Optional Arguments	Description
`-l, --longest_transcript`	Provide argument to keep only longest transcript per gene record (RECOMMENDED)
`-p, --protein_coding`	Provide argument to keep only gene records annotated as protein coding genes
`-t, --truncate`	Provide argument to truncate gene records
`--truncate_5prime`	Amount to truncate from 5' end of each transcript, requires –truncate argument (default: 45)
`--truncate_3prime`	Amount to truncate from 3' end of each transcript, requires –truncate argument (default: 15)
`-m`	Number of max processors to use for tasks (default: No limit)

Examples

Example 1 – Create longest transcript-only, protein coding-only, truncated reference:
- Keeps the longest transcript for each gene record
- Keeps only protein_coding annotated transcripts
- Truncates the first 45 nucleotides from the first exon of every transcript (default)
- Truncates the last 15 nucleotides from the last exon of every transcript (default)
- Each modification desired must be implicitly passed to the sub-module

```
$ xpresspipe modifyGTF -g /path/to/reference/transcripts.gtf --longest_transcript --
↪protein_coding --truncate
```

2.7 Single-End RNA-seq Pipeline

The following pipeline will pre-process, align, and quality check single-end RNA-seq samples using the sub-modules discussed in earlier chapters. For more detailed information concerning these steps, please refer to the appropriate chapter.

Required Arguments	Description
`-i <path>, --input <path>`	Path to input directory – if paired-end, file names should be exactly the same except for `r1/r2.fastq` or similar suffix
`-o <path>, --output <path>`	Path to output directory
`-r <path>, --reference <path>`	Path to parent organism reference directory
`-g </path/transcripts.gtf>, --gtf </path/transcripts.gtf>`	Path and file name to GTF used for alignment quantification (only used for HTSeq quantification)
`-e, --experiment`	Experiment name

Optional Arguments	Description
--two-pass	Use a two-pass STAR alignment for novel splice junction discovery
-a <adapter1 ...> [<adapter1 ...> ...], --adapter <adapter1 ...> [<adapter1 ...> ...]	Specify adapter(s) in list of strings – for single-end, only provide one adapter – if None are provided, software will attempt to auto-detect adapters – if “POLYX” is provided as a single string in the list, polyX adapters will be trimmed. If you want to auto-detect adapters in for paired-end reads, provide None twice
-q <PHRED_value>, --quality <PHRED_value>	PHRED read quality threshold (default: 28)
--min_length <length_value>	Minimum read length threshold to keep for reads (default: 17)
--umi_location <location>	Provide parameter to process UMIs – provide location (see fastp documentation for more details, generally for single-end sequencing, you would provide ‘read1’ here; does not work with -a polyX option)
--umi_length <length>	Provide parameter to process UMIs – provide UMI length (must provide the –umi_location argument); does not work with -a polyX option)
--no_multimappers>	Include flag to remove multimapping reads to be output and used in downstream analyses
--deduplicate	Include flag to quantify reads with de-duplication (will search for files with suffix _dedupRemoved.bam)
--output_bed	Include flag to output BED files for each aligned file
-c, --quantification_method	Specify quantification method (default: htseq; other option: cufflinks. If using Cufflinks, no downstream sample normalization is required)
--feature_type <feature>	Specify feature type (3rd column in GTF file) to be used if quantifying with htseq (default: CDS)
--stranded <fr-unstranded/ fr-firststrand / fr-secondstrand||no/yes>	Specify whether library preparation was stranded (Options before || correspond with Cufflinks inputs, options after correspond with htseq inputs)
--method <RPM, RPKM, FPKM, TPM>	Normalization method to perform (options: “RPM”, “TPM”, “RPKM”, “FPKM”) – if using either TPM, RPKM, or FPKM, a GTF reference file must be included
--vcf </path/to/file.vcf>	Provide full path and file name to VCF file if you would like detect personal variants overlapping alignments
--batch </path/filename.tsv>	Include path and filename of dataframe with batch normalization parameters
--sjdbOverhang <sjdbOverhang_amount>	Specify length of genomic sequences for constructing splice-aware reference. Ideal length is read length - 1, so for 2x100bp paired-end reads, you would use 100 - 1 = 99. However, the default value of 100 should work in most cases
--mismatchRatio <mismatchRatio>	Alignment ratio of mismatches to mapped length is less than this value. See STAR documentation for more information on setting this parameter
--seedSearchStartLmax <seedSearchStartLmax>	Adjusting this parameter by providing a lower number will improve mapping sensitivity (recommended value = 15 for reads ~ 25 nts). See STAR documentation for more information on setting this parameter
genome_size	Only needs to be changed if this argument was provided curing reference building AND using a two-pass alignment. This should be the length of the organism’s genome in nucleotides
-m	Number of max processors to use for tasks (default: No limit)

Run the following for more details:

```
$ xpresspipe seRNAseq --help
```

2.7.1 Examples

Example 1 – Run pipeline on single-end RNA-seq sample files

```
$ xpresspipe seRNAseq \
              -i se_test \
              -o se_out \
              -r se_reference \
              --gtf transcripts_LC.gtf \
              -e se_test \
              -a CTGTAGGCACCATCAAT \
              --method TPM \
              --sjdbOverhang 49
```

2.8 Paired-End RNA-seq Pipeline

The following pipeline will pre-process, align, and quality check paired-end RNA-seq samples using the sub-modules discussed in earlier chapters. For more detailed information concerning these steps, please refer to the appropriate chapter.

Required Arguments	Description
`-i <path>, --input <path>`	Path to input directory – if paired-end, file names should be exactly the same except for `r1/r2.fastq` or similar suffix
`-o <path>, --output <path>`	Path to output directory
`-r <path>, --reference <path>`	Path to parent organism reference directory
`-g </path/transcripts.gtf>, --gtf </path/transcripts.gtf>`	Path and file name to GTF used for alignment quantification (only used for HTSeq quantification)
`-e`, `--experiment`	Experiment name

Optional Arguments	Description
`--two-pass`	Use a two-pass STAR alignment for novel splice junction discovery
`-a <adapter1 ...> [<adapter1 ...> ...], --adapter <adapter1 ...> [<adapter1 ...> ...]`	Specify adapter(s) in list of strings – for single-end, only provide one adapter – if `None` are provided, software will attempt to auto-detect adapters – if "POLYX" is provided as a single string in the list, polyX adapters will be trimmed. If you want to auto-detect adapters in for paired-end reads, provide `None` twice
`-q <PHRED_value>, --quality <PHRED_value>`	PHRED read quality threshold (default: `28`)
`--min_length <length_value>`	Minimum read length threshold to keep for reads (default: `17`)
`--umi_location <location>`	Provide parameter to process UMIs – provide location (see fastp documentation for more details, generally for single-end sequencing, you would provide 'read1' here; does not work with -a polyX option)
`--umi_length <length>`	Provide parameter to process UMIs – provide UMI length (must provide the –umi_location argument); does not work with -a polyX option)
`--no_multimappers>`	Include flag to remove multimapping reads to be output and used in downstream analyses
`--deduplicate`	Include flag to quantify reads with de-duplication (will search for files with suffix `_dedupRemoved.bam`)
`--output_bed`	Include flag to output BED files for each aligned file
`-c`, `--quantification_method`	Specify quantification method (default: htseq; other option: cufflinks. If using Cufflinks, no downstream sample normalization is required)
`--feature_type <feature>`	Specify feature type (3rd column in GTF file) to be used if quantifying with htseq (default: CDS)
`--stranded <fr-unstranded/ fr-firststrand / fr-secondstrand||no/yes>`	Specify whether library preparation was stranded (Options before || correspond with Cufflinks inputs, options after correspond with htseq inputs)
`--method <RPM, RPKM, FPKM, TPM>`	Normalization method to perform (options: "RPM", "TPM", "RPKM", "FPKM") – if using either TPM, RPKM, or FPKM, a GTF reference file must be included
`--vcf </path/to/file.vcf>`	Provide full path and file name to VCF file if you would like detect personal variants overlapping alignments
`--batch </path/filename.tsv>`	Include path and filename of dataframe with batch normalization parameters
`--sjdbOverhang <sjdbOverhang_amount>`	Specify length of genomic sequences for constructing splice-aware reference. Ideal length is `read length - 1`, so for 2x100bp paired-end reads, you would use 100 - 1 = 99. However, the default value of `100` should work in most cases
`--mismatchRatio <mismatchRatio>`	Alignment ratio of mismatches to mapped length is less than this value. See STAR documentation for more information on setting this parameter
`--seedSearchStartLmax <seedSearchStartLmax>`	Adjusting this parameter by providing a lower number will improve mapping sensitivity (recommended value = 15 for reads ~ 25 nts). See STAR documentation for more information on setting this parameter
`genome_size`	Only needs to be changed if this argument was provided curing reference building AND using a two-pass alignment. This should be the length of the organism's genome in nucleotides
`-m`	Number of max processors to use for tasks (default: No limit)

Run the following for more details:

```
$ xpresspipe peRNAseq --help
```

2.8.1 Examples

Example 1 – Run pipeline on paired-end RNA-seq sample files

```
$ xpresspipe peRNAseq \
              -i pe_test \
              -o pe_out \
              -r pe_reference \
              --gtf transcripts.gtf \
              -e pe_test \
              -a AGATCGGAAGAGCGTCGTGTAGGGAAAGAGTGT AGATCGGAAGAGCACACGTCTGAACTCCAGTCAC␣
↪\
              --method TPM \
              --sjdbOverhang 100
```

2.9 Ribosome Profiling Pipeline

The following pipeline will pre-process, align, and quality check ribosome profiling samples using the sub-modules discussed in other sections of this documentation. For more detailed information concerning these steps, please refer to the appropriate chapter for the step you are interested in.

Required Arguments	Description
`-i <path>, --input <path>`	Path to input directory – if paired-end, file names should be exactly the same except for `r1/r2.fastq` or similar suffix
`-o <path>, --output <path>`	Path to output directory
`-r <path>, --reference <path>`	Path to parent organism reference directory
`-g </path/transcripts.gtf>, --gtf </path/transcripts.gtf>`	Path and file name to GTF used for alignment quantification (only used for HTSeq quantification)
`-e`, `--experiment`	Experiment name

Optional Arguments	Description
--two-pass	Use a two-pass STAR alignment for novel splice junction discovery
-a <adapter1 ...> [<adapter1 ...> ...], --adapter <adapter1 ...> [<adapter1 ...> ...]	Specify adapter(s) in list of strings – for single-end, only provide one adapter – if None are provided, software will attempt to auto-detect adapters – if "POLYX" is provided as a single string in the list, polyX adapters will be trimmed. If you want to auto-detect adapters in for paired-end reads, provide None twice
-q <PHRED_value>, --quality <PHRED_value>	PHRED read quality threshold (default: 28)
--min_length <length_value>	Minimum read length threshold to keep for reads (default: 17)
--umi_location <location>	Provide parameter to process UMIs – provide location (see fastp documentation for more details, generally for single-end sequencing, you would provide 'read1' here; does not work with -a polyX option)
--umi_length <length>	Provide parameter to process UMIs – provide UMI length (must provide the –umi_location argument); does not work with -a polyX option)
--no_multimappers>	Include flag to remove multimapping reads to be output and used in downstream analyses
--deduplicate	Include flag to quantify reads with de-duplication (will search for files with suffix _dedupRemoved.bam)
--output_bed	Include flag to output BED files for each aligned file
-c, --quantification_method	Specify quantification method (default: htseq; other option: cufflinks. If using Cufflinks, no downstream sample normalization is required)
--feature_type <feature>	Specify feature type (3rd column in GTF file) to be used if quantifying with htseq (default: CDS)
--stranded <fr-unstranded/ fr-firststrand / fr-secondstrand||no/yes>	Specify whether library preparation was stranded (Options before || correspond with Cufflinks inputs, options after correspond with htseq inputs)
--method <RPM, RPKM, FPKM, TPM>	Normalization method to perform (options: "RPM", "TPM", "RPKM", "FPKM") – if using either TPM, RPKM, or FPKM, a GTF reference file must be included
--vcf </path/to/file.vcf>	Provide full path and file name to VCF file if you would like detect personal variants overlapping alignments
--batch </path/filename.tsv>	Include path and filename of dataframe with batch normalization parameters
--sjdbOverhang <sjdbOverhang_amount>	Specify length of genomic sequences for constructing splice-aware reference. Ideal length is read length - 1, so for 2x100bp paired-end reads, you would use 100 - 1 = 99. However, the default value of 100 should work in most cases
--mismatchRatio <mismatchRatio>	Alignment ratio of mismatches to mapped length is less than this value. See STAR documentation for more information on setting this parameter
--seedSearchStartLmax <seedSearchStartLmax>	Adjusting this parameter by providing a lower number will improve mapping sensitivity (recommended value = 15 for reads ~ 25 nts). See STAR documentation for more information on setting this parameter
genome_size	Only needs to be changed if this argument was provided curing reference building AND using a two-pass alignment. This should be the length of the organism's genome in nucleotides
-m	Number of max processors to use for tasks (default: No limit)

Run the following for more details:

```
$ xpresspipe riboseq --help
```

2.9.1 Examples

Example 1 – Run pipeline on ribosome profiling sample files

```
$ xpresspipe riboseq \
              -i riboprof_test \
              -o ribopipe_out \
              -r se_reference \
              --gtf se_reference/transcript_CT.gtf \
              -e riboprof_test \
              -a CTGTAGGCACCATCAAT \
              --method RPM \
              --sjdbOverhang 49
```

2.10 Quality Control

2.10.1 Read Distribution Analysis

When performing RNA-seq, your sequencing library population is important to assess to ensure a quality sequencing run. Unexpected populations can be indicative of RNA degradation or other effects. In ribosome profiling, the expected footprint size is ~28-30 nucleotides, so you would expect a peak in this region when running your analysis. The following module will run read distribution analysis for all `fastq` samples within a given directory. It is recommended this analysis be performed on trimmed reads to clean up adapters and get the true distribution of sequence reads in the library. When this is run within the pipeline, it will analyze just the post-trimming `fastq` files.

Additionally, if running one of XPRESSpipe's pipelines, you can refer to the MultiQC `html` file for general summary statistics, which include read length distributions for all samples.

Arguments

The help menu can be accessed by calling the following from the command line:

```
$ xpresspipe readDistribution --help
```

Required Arguments	Description
`-i <path>, --input <path>`	Path to input directory of trimmed fastq (or untrimmed fastq) files
`-o <path>, --output <path>`	Path to output directory

Required Arguments	Description
-t <SE or PE>, --type <SE or PE>	Sequencing type ("SE" for single-end, "PE" for paired-end)
-e <experiment_name>, --experiment <experiment_name>	Experiment name
-m	Number of max processors to use for tasks (default: No limit)

Examples

Example 1 – Analyze read distributions from ribosome profiling libraries

```
$ xpresspipe readDistribution -i riboprof_out/trimmed_fastq -o riboprof_out -e se_test
```

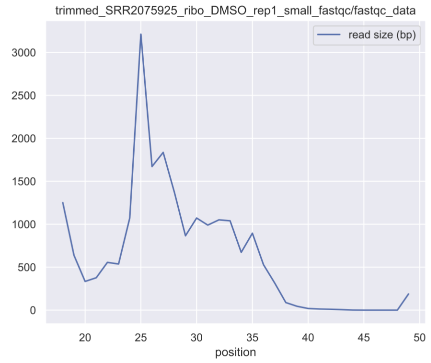

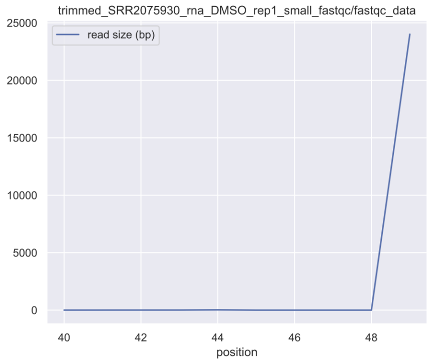

2.10.2 Metagene Analysis

Analyze each sequencing sample to ensure equal distribution of reads across all transcripts. Can be useful in identifying 5' or 3' biases in sequence preparation.

Requires a transcriptome-mapped BAM files, which can be output by STAR and are automatically output during any XPRESSpipe alignment run.

```
$ xpresspipe metagene --help
```

Required Arguments	Description
-i <path>, --input <path>	Path to input directory of transcriptome-mapped BAM files
-o <path>, --output <path>	Path to output directory
-g </path/transcripts.gtf>, --gtf </path/transcripts.gtf>	Path and file name to un-modified reference GTF

Optional Arguments	Description
-e <experiment_name>, --experiment <experiment_name>	Experiment name
--feature_type <feature_type>	Specify feature type (3rd column in GTF file) to be used in calculating metagene coverage (default: exon; alternative: CDS)
--bam_suffix <suffix>	Change from default suffix of toTranscriptome.out.bam if transcriptome-mapped files were processed outside of XPRESSpipe
-m <processors>, --max_processors <processors>	Number of max processors to use for tasks (default: No limit)

Examples

Example 1 – Analyze metagene profiles of sequence libraries

- Use default transcript reference (maps to all transcripts, even if non-coding)

```
$ xpresspipe metagene -i riboprof_out/alignments/ -o riboprof_out -g se_reference/
↪transcripts.gtf -e se_test
```

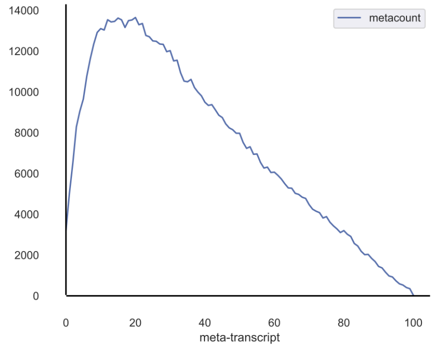

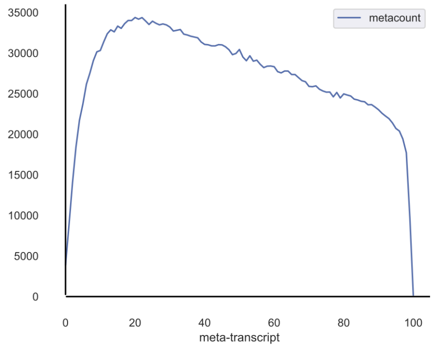

NOTE: As you can appreciate, there are systematic 5' biases in these library preparations. A good RNA-seq library should generally have even coverage across all transcripts.

2.10.3 Intron-collapsed Gene Coverage Analysis

Plot the coverage of a given gene for a sample or set of samples with introns collapsed.

```
$ xpresspipe geneCoverage --help
```

Required Arguments	Description
-i <path>, --input <path>	Path to input directory of transcriptome-aligned BAM files
-o <path>, --output <path>	Path to output directory
-g </path/transcripts.gtf>, --gtf </path/transcripts.gtf>	Path and file name to reference GTF
-n <gene_name>, --gene_name <gene_name>	Gene name (case sensitive)

Optional Arguments	Description
`-e <experiment_name>, --experiment <experiment_name>`	Experiment name to save output summaries as
`--bam_suffix <suffix>`	Change from default suffix of toTranscriptome.out.bam if using a different BAM file
`--type <type>`	Record type to map across (i.e. “exon”, “CDS”) (case-sensitive)
`--samples <sample_list> [<sample_list> ...]`	Provide a space-separated list of sample names to include in analysis (will only include those listed, and will plot in the order listed)
`--sample_names <suffix>`	Provide a space-separated list of sample names to use for labels
`--plot_color <color>`	Indicate plotting color
`-m <processors>, --max_processors <processors>`	Number of max processors to use for tasks (default: No limit)

Examples

Example 1 – Analyze gene coverage profile of sequence libraries

- Use default transcript reference (will generate a longest transcript-only reference)
- Analyze SLC1A1
- Analyze along chosen record type (default: exon, but could also use CDS if looking at ribosome profiling data)
- Analyzing BAM files ending in `sort.bam`
- Specifying names to use in plotting – if not using `--samples`, these files will be plotted alphabetically, so the listed order should also be alphabetical. If using `--samples`, need to specify names in the same order you provided for this argument.

```
$ xpresspipe geneCoverage -i /path/to/bam_files -o ./ -g /path/to/reference.gtf \
  -n SLC1A1 --type exon --bam_suffix .sort.bam \
  --sample_names SRR1795425 SRR1795433 SRR1795435 SRR1795437
```

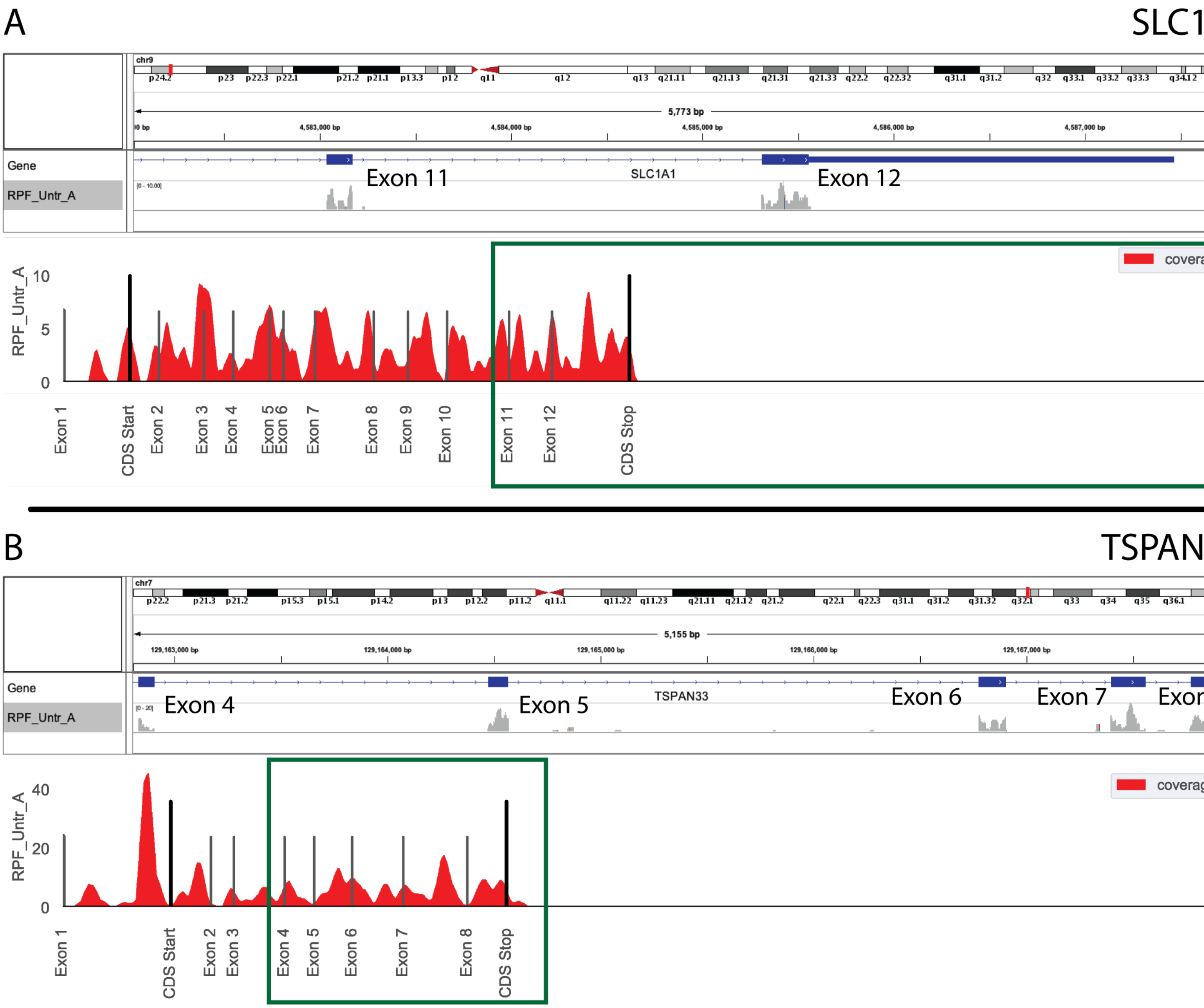

NOTE: The coverage estimations use a 20 nt rolling window mean method to smoothen the coverage plots. In both A and B in the image above, the top plot was generated with IGV (https://software.broadinstitute.org/software/igv/) and the bottom with `xpresspipe geneCoverage`. Green boxes show approximately the same region for comparison.

2.10.4 Codon Periodicity Analysis

Analyze periodicity of most abundant read length. Useful in ribosome profiling samples for identifying that ribosomes are taking the expected 3 nucleotide steps along a transcript. If this is not apparent from the analysis, it may be indicative of poor sequence coverage of the ribosome profiling libraries.

```
$ xpresspipe periodicity --help
```

Required Arguments	Description
`-i <path>, --input <path>`	Path to input directory of transcriptome-aligned BAM files
`-o <path>, --output <path>`	Path to output directory
`-g </path/transcripts.gtf>, --gtf </path/transcripts.gtf>`	Path and file name to reference GTF

Optional Arguments	Description
`-e <experiment_name>, --experiment <experiment_name>`	Experiment name to save output summaries as
`--bam_suffix <suffix>`	Change from default suffix of toTranscriptome.out.bam if using a different BAM file
`-m <processors>, --max_processors <processors>`	Number of max processors to use for tasks (default: No limit)

Examples

Example 1 – Analyze periodicity from ribosome profiling libraries

```
$ xpresspipe periodicity -i riboprof_out/alignments/ -o riboprof_out -g se_reference/
↪transcripts.gtf -e se_test
```

2.11 Analysis

2.11.1 Differential Expression Analysis

Differential Expression analysis allows one to determine significantly enriched or depleted genes between two conditions. XPESSpipe acts as a wrapper for DESeq2. Please refer to its documentation for more information.

NOTE: If intending to use the `diffxpress` sub-module, you need to have used `--quantification_method htseq` for during read quantification and DESeq2 requires integer count data.

Assumptions:

- R is installed on your machine and is in your $PATH (this should be handled in the installation)
- All input files are tab-delimited (with .txt or .tsv suffix)
- Design formula does not include the tilde (~) and there are no spaces
- Your base parameter in a given column in the sample_info file must be first alphabetically. If this is not the base, you can append letters to the beginning to force alphabetical order

Arguments

The help menu can be accessed by calling the following from the command line:

```
$ xpresspipe diffxpress --help
```

Required Arguments	Description
`-i <path/filename.tsv>, --input <path/filename.tsv>`	Path and file name of expression counts matrix
`-s <path/filename.tsv>, --sample <path/filename.tsv>`	Path and file name of sample information matrix
`--design <formula>`	Design formula for differential expression analysis (spaces in command line are conserved in input string. DO NOT INCLUDE ~ OR SPACES IN FORMULA IN COMMAND LINE, will be automatically added)

Examples

Example 1 – Analyze RNA-seq data

```
> data = pd.read_csv('/path/to/expression_counts.tsv', index_col=0)
> data
                s1  s2  s3  s4  ...
ENSG00000227232 66  59  1   82  ...
ENSG00000240361 35  0   7   72  ...
ENSG00000238009 20  70  85  78  ...
ENSG00000241860 96  7   93  38  ...
ENSG00000187634 73  41  92  77  ...
> batch = pd.read_csv('/path/to/sample_info.tsv', index_col=0)
> batch
  Sample  Replicate Condition
0 s1      rep1      a_WT
1 s2      rep2      a_WT
2 s3      rep1      a_WT
3 s4      rep2      a_WT
4 s5      rep1      b_EXP
5 s6      rep2      b_EXP
6 s7      rep1      b_EXP
7 s8      rep2      b_EXP
```

```
$ xpresspipe diffxpress -i test_r/test_dataset.tsv --sample test_r/sample_info.tsv --
↪design Condition
```

Example 2 – Analyze RNA-seq data that was prepared in different batches:

```
> data = pd.read_csv('/path/to/expression_counts.tsv', index_col=0)
> data
                s1  s2  s3  s4  ...
ENSG00000227232 66  59  1   82  ...
ENSG00000240361 35  0   7   72  ...
ENSG00000238009 20  70  85  78  ...
ENSG00000241860 96  7   93  38  ...
ENSG00000187634 73  41  92  77  ...
```

(continues on next page)

(continued from previous page)

```
> batch = pd.read_csv('/path/to/sample_info.tsv', index_col=0)
> batch
  Sample  Replicate Condition Batch
0 s1      rep1      a_WT      batch1
1 s2      rep2      a_WT      batch1
2 s3      rep1      a_WT      batch1
3 s4      rep2      a_WT      batch1
4 s5      rep1      b_EXP     batch2
5 s6      rep2      b_EXP     batch2
6 s7      rep1      b_EXP     batch2
7 s8      rep2      b_EXP     batch2
```

```
$ xpresspipe diffxpress -i test_r/test_dataset.tsv --sample test_r/sample_info.tsv --
↪design Condition+Batch
```

Example 3 – Analyze ribosome profiling data:
For ribosome profiling, you need to divide the footprint samples by their corresponding mRNA sample to account for translation efficiency

```
> data = pd.read_csv('/path/to/expression_counts.tsv', index_col=0)
> data
                s1_fp   s1_rna  s2_fp   s2_rna  ...
ENSG00000227232 66      59      1       82      ...
ENSG00000240361 35      0       7       72      ...
ENSG00000238009 20      70      85      78      ...
ENSG00000241860 96      7       93      38      ...
ENSG00000187634 73      41      92      77      ...
> batch = pd.read_csv('/path/to/sample_info.tsv', index_col=0)
> batch
  Sample  Replicate Condition Type
0 s1_fp   rep1      a_WT      RPF
1 s1_rna  rep1      a_WT      RNA
2 s2_fp   rep2      a_WT      RPF
3 s2_rna  rep2      a_WT      RNA
4 s3_fp   rep1      b_EXP     RPF
5 s3_rna  rep1      b_EXP     RNA
6 s4_fp   rep2      b_EXP     RPF
7 s4_rna  rep2      b_EXP     RNA
```

```
$ xpresspipe diffxpress -i test_r/test_dataset.tsv --sample test_r/sample_info.tsv --
↪design Type+Condition+Type:Condition
```

2.11.2 rRNA Probe

Ribosome RNA (rRNA) contamination is common in RNA-seq library preparation. As the bulk of RNA in a cell at any given time is dedicated to rRNA, and as these rRNA sequences are relatively few and therefore highly repeated, depletion of these sequences is often desired in order to have better depth of coverage of non-rRNA sequences. In order to facilitate this depletion, many commercial kits are available that target specific rRNA sequences for depletion, or that enrich mRNA polyA tails. However, and especially in the case of ribosome profiling experiments, where RNA is digested to create ribosome footprints that commercial depletion kits won't detect and polyA selection

kits are inoperable as footprints will not have the requisite polyA sequence. To this end, custom rRNA probes are recommended, and the `rrnaProbe` sub-module was designed to facilitate this process.

`rrnaProbe` works by doing the following:

1. Run FASTQC to detect over-represented sequences
2. Collate these sequences to determine consensus fragments
3. Output rank ordered list of over-represented fragments within the appropriate length range to target for depletion

NOTE: BLAST capability to verify over-represented consensus fragments are indeed rRNA sequences is not yet incorporated, so any sequences that will be used as probes should be BLAST-verified first.

```
$ xpresspipe rrnaProbe --help
```

Required Arguments	Description
`-i <path>, --input <path>`	Path to zipped FASTQC files
`-o </path/filename>, --output </path/filename>`	Path and file name to write output

Optional Arguments	Description
`-m <value>, --min_overlap <value>`	Minimum number of bases that must match on a side to combine sequences (default: 5)
`--footprint_only`	Only take zip files that are ribosome profiling footprints (file names must contain “FP”, “RPF”, or “FOOTPRINT”)

Examples

Example 1 – Generate rank-ordered list of over-represented sequences

```
$ xpresspipe rrnaProbe -i riboprof_out/fastqc_out/ -o riboprof_out/sequences.txt --
↪footprint_only

TTGATGATTCATAATAACTTTTCGAATCGCAT    514832
TATAAATCATTTGTATACGACTTAGAT         121739
TTGATGATTCATAATAACTTTTCGAATCGCAT    15776
TTTGATGATTCATAATAACTTTTCGAATCGCAC   33325
ATAAATCATTTGTATACGACTTAGAC          13603
```

2.12 Read Pre-Processing

2.12.1 Read Trimming

Trimming is a necessary part of RNAseq data processing due to the technological limitations described below:

- Inherent in RNA-seq library creation, RNA is fragmented and adapter sequences are ligated to the sequence. These adapters include information such as sample batch and act as a primer for the sequencer to recognize the fragment as something to analyze. However, these adapters, once sequenced, prevent alignment to a reference as large chunks of the fragment are synthetic sequence not found in the actual organism’s genome/transcriptome.

- A sequencer’s job is to read a fragment base by base and determine the nucleotide species each step of the way. While the technology has greatly improved over the years, a probability of error remains. Mis-called bases can

prevent proper alignment of the sequenced fragment to the reference. Therefore, it is important for low confidence base calls to be trimmed from each read.

Trimming is performed by fastp

Arguments

The help menu can be accessed by calling the following from the command line:

```
$ xpresspipe trim --help
```

Required Arguments	Description
-i <path>, --input <path>	Path to input directory – if paired-end, file names should be exactly the same except for r1/r2.fastq or similar suffix
-o <path>, --output <path>	Path to output directory

Optional Arguments	Description
-a <adapter1 ...> [<adapter1 ...> ...], --adapter <adapter1 ...> [<adapter1 ...> ...]	Specify adapter(s) in list of strings – for single-end, only provide one adapter – if None are provided, software will attempt to auto-detect adapters – if "POLYX" is provided as a single string in the list, polyX adapters will be trimmed. If you want to auto-detect adapters in for paired-end reads, provide None twice
-q <PHRED_value>, --quality <PHRED_value>	PHRED read quality threshold (default: 28)
--min_length <length_value>	Minimum read length threshold to keep for reads (default: 17)
--umi_location <location>	Provide parameter to process UMIs – provide location (see fastp documentation for more details, generally for single-end sequencing, you would provide 'read1' here; does not work with -a polyX option)
--umi_length <length>	Provide parameter to process UMIs – provide UMI length (must provide the –umi_location argument); does not work with -a polyX option)
-m	Number of max processors to use for tasks (default: Max)

Examples

Example 1 – Trim ribosome profiling (or single-end) sequence data using default preferences:

- Raw reads are fastq-like and found in the -i riboprof_test/ directory. Can be uncompressed or compressed via gz or zip
- A general output directory has been created, -o riboprof_out/
- All other arguments use the default value

```
$ xpresspipe trim -i riboprof_test/ -o riboprof_out/
```

Example 2 – Predict adapter and trim ribosome profiling (or single-end) sequence data:

- A minimum read length of 22 nucleotides after trimming is required in order to keep the read
- A maximum or 6 processors can be used for the task
- The `--adapters` argument was not passed, so an attempt to discover adapter sequences will be made (this is not always the most efficient or thorough method of trimming and providing the adapter sequences is recommended)

```
$ xpresspipe trim -i riboprof_test/ -o riboprof_out/ --min_length 22 -m 6
```

Example 3 – Pass explicit adapter trim ribosome profiling (or single-end) sequence data:
- The default minimum read length threshold will be used
- The maximum number of processors will be used by default
- The `--adapters` argument was passed, so adapter sequences will trimmed explicitly

```
$ xpresspipe trim -i riboprof_test/ -o riboprof_out/ -a CTGTAGGCACCATCAAT
```

Example 4 – Predict adapter and trim paired-end sequence data:
- The `--adapters` argument was passed as `None None`, so an attempt to discover adapter sequences will be made for paired-end reads. The `-a None None` syntax is essential for `trim` to recognize the reads as paired-end

```
$ xpresspipe trim -i pe_test/ -o pe_out/ -a None None
```

Example 5 – Pass explicit adapter and trim paired-end sequence data:
- The `--adapters` argument was passed, so adapter sequences will trimmed explicitly

```
$ xpresspipe trim -i pe_test/ -o pe_out/ -a ACACTCTTTCCCTACACGACGCTCTTCCGATC␣
↪GATCGGAAGAGCGGTTCAGCAGGAATGCCGAG
```

Example 6 – Trim single-end sequence data of polyX adapters:
- The `--adapters POLYX` argument was passed, so adapter sequences will trimmed of polyX sequences

```
$ xpresspipe trim -i se_test/ -o se_out/ -a POLYX
```

2.13 Alignment

In order to quantify transcription on a transcript to transcript basis, individual reads called during sequencing must be mapped to the genome. While there are multiple alignment software packages available, XPRESSpipe uses a current version of STAR to perform this step in transcription quantification for several reasons:
- Performance: While computationally greedy (a human genome alignment requires upwards of 30 Gb RAM), the performance and accuracy is superior to the majority of other splice aware aligners currently available

- Splice Junction Aware: STAR is capable of mapping reads spanning a splice junction, where more traditional packages, such as Bowtie, are incapable of doing so and are better suited for tasks such as genome alignment.
- Standard: The foundation of the pipeline used in XPRESSpipe is based in the TCGA standards for RNAseq alignment. This method utilizes a guided or 2-pass alignment program. In the guided alignment, a GTF with annotated splice junctions is used to guide the alignments over splice juntions. In the 2-pass alignment, reads are mapped across the genome to identify novel splice junctions. These new annotations are then incorporated into the reference index and reads are re-aligned with this new reference. While more time-intensive, this step can aid in aligning across these junctions, especially in organisms where the transcriptome is not as well annotated.
- Variant Aware: The user can provide a VCF, such as those provided by ClinVar and dbSNP. These files are useful in integrating information about common or disease nucleotide variants into the RNA-Seq alignment stage. The files you use should match the build of the genome you are using (i.e., if using Homo Sapiens GRCh38, these builds should match between curated reference files and VCF file).

2.13.1 Single-End RNAseq Alignment

The following runs single-end reads alignment using the specified XPRESSpipe-formatted reference directory.

Notes:

- For the `--sjdbOverhang` argument, the same value should be entered that was used when creating the STAR reference files.
- Ribosome profiling data can be run as a single-end RNA-seq

Arguments

The help menu can be accessed by calling the following from the command line:

```
$ xpresspipe align --help
```

Required Arguments	Description
`-i <path>, --input <path>`	Path to input directory
`-o <path>, --output <path>`	Path to output directory
`-r <path>, --reference <path>`	Path to parent organism reference directory (must have a file called transcripts.gtf within)
`-t <SE or PE>, --type <SE or PE>`	Sequencing type ("SE" for single-end, "PE" for paired-end)

Optional Arguments	Description
`--two-pass`	Use a two-pass STAR alignment for novel splice junction discovery
`--no_multimappers>`	Include flag to remove multimapping reads to be output and used in downstream analyses
`--deduplicate`	Include flag to quantify reads with de-duplication (will search for files with suffix `_dedupRemoved.bam`)
`--vcf </path/to/file.vcf>`	Provide full path and file name to VCF file if you would like detect personal variants overlapping alignments
`--output_bed`	Include flag to output BED files for each aligned file
`--sjdbOverhang <sjdbOverhang_amount>`	Specify length of genomic sequences for constructing splice-aware reference. Ideal length is `read length - 1`, so for 2x100bp paired-end reads, you would use 100 - 1 = 99. However, the default value of `100` should work in most cases
`--mismatchRatio <mismatchRatio>`	Alignment ratio of mismatches to mapped length is less than this value. See STAR documentation for more information on setting this parameter
`--seedSearchStartLmax <seedSearchStartLmax>`	Adjusting this parameter by providing a lower number will improve mapping sensitivity (recommended value = 15 for reads ~ 25 nts). See STAR documentation for more information on setting this parameter
`genome_size`	Only needs to be changed if this argument was provided curing reference building AND using a two-pass alignment. Enter the size of your organism's genome in nucleotides
`-m`	Number of max processors to use for tasks (default: No limit)

Examples

Example 1 – Single-end RNAseq alignment:

- Raw reads are `fastq`-like and found in the `-i /path/to/input/files/` directory. Can be uncompressed or compressed via `gz` or `zip`
- A general output directory has been created, `-o riboseq_out/`
- `--type` is specified as 'SE' and path to parent reference directory is provided
- The value for `--sjdbOverhang` used in reference creation is provided. Failure to do so will trigger an error
- BED and BIGWIG files will be output in their own directories in `output`
- All other arguments use the default value

```
$ xpresspipe align -i /path/to/input/files/ -o riboseq_out/ -t SE -r /path/to/
↪reference/ --sjdbOverhang 49 --output_bed --output_bigwig
```

2.13.2 Paired-End RNAseq Alignment

The following runs paired-end reads alignment using the specified XPRESSpipe-formatted reference directory.
Notes:
- For the `--sjdbOverhang` argument, the same value should be entered that was used when creating the STAR reference files.

Arguments

The help menu can be accessed by calling the following from the command line:

```
$ xpresspipe align --help
```

Required Arguments	Description
`-i <path>, --input <path>`	Path to input directory
`-o <path>, --output <path>`	Path to output directory
`-r <path>, --reference <path>`	Path to parent organism reference directory
`-t <SE or PE>, --type <SE or PE>`	Sequencing type ("SE" for single-end, "PE" for paired-end)

Optional Arguments	Description
`--output_bed`	Include flag to output BED files for each aligned file
`--output_bigwig`	Include flag to output bigwig files for each aligned file
`--sjdbOverhang <sjdbOverhang_amount>`	Specify length of genomic sequences for constructing splice-aware reference. Ideal length is `read length - 1`, so for 2x100bp paired-end reads, you would use 100 - 1 = 99. However, the default value of `100` should work in most cases
`-m`	Number of max processors to use for tasks (default: No limit)

Examples

Example 1 – Paired-end RNAseq alignment:
- Raw reads are `fastq`-like and found in the `-i pe_test/` directory. Can be uncompressed or compressed via `gz` or `zip`
- A general output directory has been created, `-o pe_out/`
- `--type` is specified as 'PE' and path to parent reference directory is provided
- The value for `--sjdbOverhang` used in reference creation is provided. Failure to do so will trigger an error. In this case, since the reference was created using default values, the optional flag is not used
- BED and BIGWIG files are not output
- All other arguments use the default value

```
$ xpresspipe align -i /path/to/input/files/ -o riboseq_out -t PE -r /path/to/
↪reference/
```

2.14 Read Quantification

2.14.1 Quantifying and Collating Reads

In order to quantify aligned reads, they must be counts to a reference transcriptome. This will tell you in relative terms how much of each transcript is expressed in a system. The following sub-module will perform this quantification, as well as compile all sample quantifications into a single data matrix for downstream use.

XPRESSpipe uses Cufflinks as the default, but HTSeq can also be specified. Cufflinks is one of the most accurate read quantifiers currently available, but HTSeq is still widely used and is part of the TCGA pipeline.

Arguments

The help menu can be accessed by calling the following from the command line:

```
$ xpresspipe count --help
```

Required Arguments	Description
-i <path>, --input <path>	Path to input directory of SAM files
-o <path>, --output <path>	Path to output directory
-g </path/transcripts.gtf>, --gtf </path/transcripts.gtf>	Path and file name to GTF used for alignment quantification (if a modified GTF was created, this should be provided here; if using Cufflinks and you want isoform abundance estimates, important that you do not provide a longest transcript only GTF)

Optional Arguments	Description
-e, --experiment	Experiment name
-c, --quantification_method	Specify quantification method (default: htseq; other option: cufflinks. If using Cufflinks, no downstream sample normalization is required)
--feature_type <feature>	Specify feature type (3rd column in GTF file) to be used if quantifying with htseq (default: CDS)
--stranded <fr-unstranded/ fr-firststrand / fr-secondstrand||no/yes>	Specify whether library preparation was stranded (Options before || correspond with Cufflinks inputs, options after correspond with htseq inputs)
--deduplicate	Include flag to quantify reads with de-duplication (will search for files with suffix _dedupRemoved.bam)
--bam_suffix	Change from default suffix of _Aligned.sort.bam
-m	Number of max processors to use for tasks (default: No limit)

Examples

Example 1 – Count ribosome profiling alignments:
- Input points to directory with SAM alignment files that are sorted by name
- An experiment name is provided to name the final data matrix
- Reads are quantified only to coding genes and are not counted if mapping to the first x nucleotides of each transcript exon 1 (x being the value provided for truncation when initially creating the reference files)

```
$ xpresspipe count -i riboseq_out/alignments/ -o riboseq_out/ -r se_reference/ -g se_
↪reference/transcripts_codingOnly_truncated.gtf -e se_test
```

Example 2 – Count paired-end alignments:
- Input points to directory with SAM alignment files that are sorted by name

- An experiment name is not provided and a default name is given to the data matrix using datatime
- Reads are quantified to the entire transcriptome (coding and non-coding, no truncation)

```
$ xpresspipe count -i pe_out/alignments/ -o pe_out/ -r pe_reference/
```

2.15 Normalize

Note: Sample and batch normalization can be performed in a single command. If this is done, batch normalization will be performed following sample normalization.

2.15.1 Sample Normalize

Due to inherent biases in RNA-seq samples (most commonly, different amounts of total RNA per sample in a given lane), samples must be normalized to obtain an accurate representation of transcription per sample. Additional normalization can be performed to normalize for transcript length ("per kilobase million") as longer transcripts will naturally create more fragments mapping to a given gene, thus potentially making 1 transcript appear as many when quantified.

The following equations summarize different way to normalize samples for RNA-seq:

- **Reads per Million**
$RPM_g = \frac{1e6 \cdot r_{ge}}{\sum_{g=1}^{n} r_{ge}}$
- **Reads per Kilobase of Reads per Million**
$RPKM_g = \frac{1e9 \cdot r_{ge}}{(\sum_{g=1}^{n} r_{ge}) \cdot l_{ge}}$
- **Fragments per Kilobase of Fragments per Million**
$FPKM_g = \frac{1e9 \cdot f_{ge}}{(\sum_{g=1}^{n} f_{ge}) \cdot l_{ge}}$
- **Transcripts per Million** (same as RPKM, but order of operations is different)
$TPM_g = \frac{1e6 \cdot r_{ge}}{(\sum_{g=1}^{n} (\frac{1e3 \cdot r_{ge}}{l_{ge}})) \cdot l_{ge}}$

In each of the above, assume *g* is gene *n*, *ge* is cumulative exon space for gene *n*, *r* is total reads, *f* is total fragments, and *l* is length

Assumptions:
- R is installed on your machine and is in your $PATH if using the `batch` argument
- All input files are tab-delimited (with .txt or .tsv suffix)

Batch Correction:
When multiple people perform library preparation, or when libraries are prepared on different days, this can lead to inherent biases in count distributions between batches of samples. It is therefore necessary to normalize these effects when appropriate.

Arguments

The help menu can be accessed by calling the following from the command line:

```
$ xpresspipe normalizeMatrix --help
```

Required Arguments	Description
`-i <path/filename.tsv>, --input <path/filename.tsv>`	Path and file name of expression counts matrix

Optional Arguments	Description
`--method <RPM, RPKM, FPKM, LOG>`	Normalization method to perform (options: "RPM", "TPM", "RPKM", "FPKM") – if using either TPM, RPKM, or FPKM, a GTF reference file must be included
`-g </path/transcripts.gtf>, --gtf </path/transcripts.gtf>`	Path and file name to reference GTF (RECOMMENDED: Do not use modified GTF file)
`--batch </path/filename.tsv>`	Include path and filename of dataframe with batch normalization parameters

Examples

Example 1 – Perform RPKM normalization on single-end RNA-seq data:

```
$ xpresspipe normalizeMatrix -i riboprof_out/counts/se_test_counts_table.tsv --method␣
↪RPKM -g se_reference/transcripts_coding_truncated.gtf
```

Example 2 – Perform batch normalization on RNA-seq data:

```
> batch = pd.read_csv('./riboprof_out/counts/batch_info.tsv', sep='\t', index_col=0)
> batch
  Sample  Batch
0 s1      batch1
1 s2      batch2
2 s3      batch1
3 s4      batch2
```

```
$ xpresspipe normalizeMatrix -i riboprof_out/counts/se_test_counts_table.tsv --batch␣
↪riboprof_out/counts/batch_info.tsv
```

2.16 Other Features

2.16.1 Convert Counts Table Gene Names

Count tables are often produced with systematic names used to label each gene. The following sub-module will convert the column of systematic gene names to a common name using a reference GTF file

Arguments

The help menu can be accessed by calling the following from the command line:

```
$ xpresspipe convertNames --help
```

Required Arguments	Description
`-i <path/filename>, --input <path/filename>`	Path and file name to sequence dataframe
`-g </path/transcripts.gtf>, --gtf </path/transcripts.gtf>`	Path and file name to GTF

Optional Arguments	Description
`--orig_name_label <label>`	Label of original name (usually "gene_id ")
`--orig_name_location <position>`	Position in last column of GTF where relevant data is found (i.e. 0 would be the first sub-string before the first comma, 3 would be the third sub-string after the second comma before the third comma)
`--new_name_label <label>`	Label of original name (usually "gene_id ")
`--new_name_location <position>`	Position in last column of GTF where relevant data is found (i.e. 0 would be the first sub-string before the first comma, 3 would be the third sub-string after the second comma before the third comma)
`--refill <label>`	In some cases, where common gene names are unavailable, the dataframe will fill the gene name with the improper field of the GTF. In this case, specify this improper string and these values will be replaced with the original name

Examples

Example 1 – Convert gene names in count dataframe

```
$ xpresspipe convertNames -i riboprof_out/counts/se_test_counts_table.csv -g se_
↪reference/transcripts.gtf
```

2.17 FAQs

If you have questions, requests, or bugs to report, please use the XPRESSpipe issues forum.

As this software is used more, we will compile some of the most commonly asked questions here…

2.17.1 A step of the pipeline is erroring for no apparent reason?

First, please check the output in your terminal, along with in the log file. If the step that the pipeline breaks on does not output any useful information, check that the required dependencies were installed correctly. For example, when we were testing the the `geneCoverage` module on a supercomputing cluster, the pipeline responded saying it

couldn't find the appropriate index file. It turned out the R package, GenomicFeatures was not downloaded due to issues with the rtracklayers package. For this situation, we fixed it by uninstalling Anaconda and reinstalling the dependencies, as below:

```
# Run each of these steps. If a command doesn't work, skip to the next one
$ conda install anaconda-clean
$ anaconda-clean --yes
$ rm -rf ~/miniconda
$ rm ~/.condarc
$ rm -r ~/.conda/
```

```
$ cd ~
$ curl -O https://repo.anaconda.com/miniconda/Miniconda3-latest-Linux-x86_64.sh
$ bash ~/Miniconda3-latest-Linux-x86_64.sh
$ conda env base --file XPRESSpipe/requirements.yml
```

During Anaconda installation, reply `yes` to all prompts. If you wish to install the XPRESSpipe dependencies to their own environment, replace `base` with `your_environment_name_here` in the last step. If XPRESSpipe continues to malfunction after completion of these steps, please reach out to us on the XPRESSpipe issues forum.

2.17.2 The pipeline breaks because of a segmentation fault during alignment.

Occasionally, depending on allocation of CPUs, 32 virtual CPUs may be available, but only 16 are configured. This may lead to memory overloads by trying to use more than configured, as the large index files will be temporarily copied to each processing core. If this is the case, provide the `max_processors` with the number in the log file stated as available. For a computing node with 64 GB of RAM available, we generally see that 20 CPUs is stable. See log example below:

```
sh: line 1: 70311 Segmentation fault      STAR --runThreadN 30 ...

or

WARNING: fastp uses up to 16 threads although you specified 32
```

2.18 Updates

2.18.1 v0.3.1

- Fix BAM file threshold for metagene and geneCoverage to avoid OOM errors
- Turn off BAM file threshold for counting (low memory footprint, so can use all cores available)
- Import openssl library manually in Rperiodicity – occasionally had trouble finding the library on its own and would error

2.18.2 v0.3.0

- Transfers R dependency installs to Anaconda environment load

- Modified fastq and bam memory factor to optimize resources
- Rebuilt read distribution module with JuliaLang for super memory efficiency during parallelization
- Fixed issue where onel -exon genes would not display feature annotations in *geneCoverage* modules
- Made matplotlib backend calls flexible for HPC usage
- Made directory checks more thorough
- Fixed a potential offl -byl -one issue with GTF truncator
- Updated appropriate tests
- Updates to documentation
- Added code of conduct and contributions information

2.18.3 v0.2.4-beta

- Manuscript submission version
- Fixed issues with using polyX adaptors
- Allowed more multi-threading during post-processing of aligned reads to use resources more efficiently
- Added integrated pipeline tests for Travis CI build to assess pipeline integrity each push
- Updated install walkthrough video

CHAPTER 3

License

XPRESSpipe and the XPRESSyourself suite is developed and maintained by Jordan Berg in the Rutter Lab @ the University of Utah, along with other collaborators. We welcome pull requests if you would like to contribute to the project.

XPRESSpipe is perpetually open source under a GNU General Public License (v3.0).

CHAPTER 4

Questions?

If you have questions, requests, or bugs to report, please use the XPRESSpipe issues forum.
