# Supplementary material for: XPRESSyourself: Enhancing, standardizing, and automating ribosome profiling computational analyses yields improved insight into data
Source: PLoS Comput Biol. 2020 Jan 31;16(1):e1007625. doi: 10.1371/journal.pcbi.1007625 (PMC7015430; doi:10.1371/journal.pcbi.1007625)
Supplement: S3 File — Archived version of the XPRESSplot documentation. Current documentation can be found at https://xpressplot.readthedocs.io/en/latest/. (PDF) [file pcbi.1007625.s003.pdf]

xpressplot Documentation

Release 0.2.2

Jordan A. Berg

Oct 29, 2019

Contents

1 About 3

2 Table of contents 5
- 2.1 Wait! I've never programmed before! 5
- 2.2 Installation 7
- 2.3 General Usage 8
- 2.4 Retrieving Data 8
- 2.5 Normalization and Quality Control 17
- 2.6 Analysis 24
- 2.7 Updates 43

3 License 45

4 Questions? 47

CHAPTER 1

About

XPRESSplot is a part of the XPRESSyourself suite of sequencing tools. XPRESSplot is a Python toolkit for navigating and analyzing gene expression (Microarray or RNAseq) datasets. Features include the ability to import GEO-accessible datasets and metadata or your own datasets, clean, normalize, and analyze sequence data.

XPRESSplot and the XPRESSyourself suite is developed and maintained by Jordan Berg in the Rutter Lab @ the University of Utah, along with other collaborators.

CHAPTER 2

Table of contents

2.1 Wait! I've never programmed before!

If you don't have any programming experience and find this all very daunting, this is the documentation for you! We will walk through installation and usage step by step, and explain what we are doing along the way.

2.1.1 Installation

Installation requires the use of the command line interface (CLI). If you would like some background on how this programming environment works, you can try the Codecademy module which will familiarize you with this language

To begin, you will need to install the software package, xpressplot. To do so, we will use a Package Manager which will ease the overhead involved in installing software and other software packages it relies on.

1. We will need to use the command line interface (CLI, also known as the Terminal) to install and begin using the software

- Linux: Press Ctrl + Alt + T on your keyboard and the Terminal will open

- Mac: Click on the Finder icon (a magnifying glass) at the top right corner of your Desktop, type in Terminal, and double-click the corresponding icon

2. We recommend using Python3 as Python2 is being deprecated (will no longer be updated, debugged, etc)

- You can check the version of Python you have by typing `python -v` in the command line

- If you only have Python2 installed and want to use Python3, you can download this here

- Now we need the computer to recognize Python3 as the default Python by typing `newalias() {echo "python=python3" >> $HOME/.bash_aliases; source ~/.bash_aliases; }`

- Now we can test this by executing `python -v` again

3. With newer versions of Python, the package manager PyPi should already by installed. We can install xpressplot by executing the following: `pip install xpressplot`. This should install xpressplot and all dependencies.

4. Let's test that the installation worked:

```
$ python
```

This will open the python interactive mode. Next, type the following:

```
>>> import xpressplot
```

If the command executes without error, xpressplot and all dependencies have been successfully installed.

NOTE: If any installation up to this point fails due to priviledges warnings, you should run the pip install or other command using `sudo`. You will append this to the beginning of the command and will likely be asked to provide your account password for your machine. `sudo` means "substitute user do", which essentially tells your computer you are a authorized user to install software on the system.

2.1.2 Use

Assuming you are a beginner user, you will likely want to run the interactive notebook. This has many example functions you can run with a toy dataset, which can be easily modified for your use. Instructions are provided with each block of code. In order to run this interactive notebook, we will need Jupyter Notebook, which is automatically installed with the Anaconda package manager

1. Let's install Anaconda

- The version you install depends on the version of Python you are using

- Follow this link to install the appropriate version of Anaconda

2. Let's check that Anaconda installed correctly:

```
$ conda update conda
```

3. Now, let's update Jupyter notebook

```
$ conda install jupyter
```

4. Now we can open Jupyter notebook:

```
$ cd /path/to/notebook/
```

This process of navigating to the directory with the notebook can be made easy by typing :data:'cd ' and dragging and dropping the directory icon to the CLI and pressing Enter

```
$ jupyter notebook
```

This will launch a browser window with Jupyter. Now we can open the `example_notebook.ipynb` file and start running the analysis.

Execute blocks of code in the Jupyter Notebook by pressing Shift + Enter

And that's about it! Feel free to submit any issues you have here. It is helpful to copy and paste any errors that appear or the lines of code you are struggling with.

2.2 Installation

2.2.1 PyPi Install

1) Install xpressplot and associated dependencies via pip:

```
$ pip install xpressplot
```

2.2.2 Conda Install

This feature is not yet available. . .

1) Install xpressplot and associated dependencies via conda:

```
$ conda install -y -c bioconda xpressplot
```

2.2.3 Manual install

1) Or download xpressplot manually:

```
$ git clone https://github.com/XPRESSyourself/xpressplot.git
$ cd xpressplot
$ cd python setup.py install
```

2) Or, to download specific version:

```
$ tag='v0.0.1-beta'
$ wget https://github.com/XPRESSyourself/xpressplot/archive/$tag.zip
$ unzip xpressplot-${tag:1}.zip
$ mv xpressplot-${tag:1} xpressplot
$ cd xpressplot
$ cd python setup.py install
```

3) At the end of the installation instructions, an installation location will be given. Add this to your $PATH:

```
...
Installing xpressplot script to /Users/$USERNAME/anaconda3/bin

Installed /Users/$USERNAME/anaconda3/lib/python3.6/site-packages/xpressplot-0.0.1b0-
↪py3.6.egg
Processing dependencies for xpressplot==0.0.1b0
Finished processing dependencies for xpressplot==0.0.1b0

$ echo "export PATH='/Users/$USERNAME/anaconda3/bin:$PATH' >> ~/.bash_profile
```

2.3 General Usage

xpressplot is intended as a all-in-one toolkit and interface for analysis of sequencing data

2.3.1 Sequence Data

Required format for all functions (unless otherwise noted in documentation).

```
>>> geo.head()
            GSM523242  GSM523243  GSM523244  GSM523245  GSM523246   GSM523247    ...
1007_s_at   8.98104    8.59941    8.25395    8.72981    8.70794     8.10693    ...
1053_at     5.84313    6.59168    8.27881    6.64005    4.65107     7.19090    ...
121_at      6.17189    5.73603    5.55673    5.69374    6.77618     5.84524    ...
1294_at     6.97009    6.80003    5.56620    7.43816    7.36375     5.85687    ...
1405_i_at   10.24611   10.13807   8.84743    9.72365    10.42940    9.17510    ...
```

2.3.2 Metadata

Required format for all functions (unless otherwise noted in documentation).

```
>>> geo.head()
    0                  1
0     GSM523242       mucosa_normal_colon_1 (micro)
1     GSM523243       mucosa_normal_colon_2 (micro)
2     GSM523244       mucosa_adenoma_3 (micro)
3     GSM523245       colonic_crypt_epithelial_cells_normal_colon_4 ...
4     GSM523246       mucosa_normal_colon_5 (micro)
```

2.4 Retrieving Data

2.4.1 Importing Data from File

xpressplot.get_df (file_name, delimiter=',', low_memory=False, gene_axis='row')

Purpose:

Get sequence dataframe from user-provided file.

Assumptions:

- Dataset does not contain axis labels (i.e. a column header for 'gene names')
- Dataset only has gene names and sample_ids as column headers and row indices. Orientation is flexible, but needs to be specified in options if genes are not rows
- If orientation is not default, it is then specified or else function will not be able to properly format the dataframe for downstream application

Parameters:
file_name: Full path of file to import into pandas dataframe
delimiter: Delimiter type for importing file, default: ','
low_memory: Specify memory limits for importing large files, default: False (allows for large imports)
gene_axis: Orientiation of the data, where categorical data is either column-wise, (default: 'col') or row-wise ('row'). Case insensitive

Returns:
data: Pandas dataframe with data matrix

Examples:

```
> import pandas as pd
> import xpressplot as xp
> data = xp.get_df('/path/to/data.csv')
> data
              GSM523242 GSM523243 GSM523244 GSM523245 ...
1007_s_at     8.98104   8.59941   8.25395   8.72981   ...
1053_at       5.84313   6.59168   8.27881   6.64005   ...
121_at        6.17189   5.73603   5.55673   5.69374   ...
1294_at       6.97009   6.80003   5.56620   7.43816   ...
...           ...       ...       ...       ...       ...
```

2.4.2 Importing metadata from file

xpressplot.get_info (file_name, delimiter=",", axis="col", sample_ids=0, labels=1)

Purpose:
Get sample metadata from user-provided file

Assumptions:
* Data categories are not labeled
* If orientation is not default, it is then specified or else function will not be able to properly format the dataframe for downstream application

Parameters:
file_name: full path of file to import into pandas dataframe
delimiter: delimiter type for importing file, default: ','

axis: Orientiation of the data, where categorical data is either column-wise, (default: 'col') or row-wise ('row'). Case insensitive
sample_ids: Column or row number where sample IDs are found (default: 0)
labels: Column or row number where categorical label data are found (default: 1)

Returns:
metadata: Pandas dataframe with metadata

Examples:

```
> import pandas as pd
> import xpressplot as xp
> metadata = xp.get_info('/path/to/metadata.csv')
> metadata
    0         1
0   GSM523242 mucosa_normal_colon
1   GSM523243 mucosa_normal_colon
2   GSM523244 mucosa_adenoma
3   GSM523245 colonic_crypt_epithelial_cells_normal_colon
... ...       ...
```

2.4.3 Importing data from GEO

RNAseq Datasets

A module will be added in the future to automate this conversion and import from GEO
Download the csv or tsv file provided in supplement and ensure formatted follows xpressplot standards
Sometimes the delimiter is formatted incorrectly. If so, a simple find/replace can be used to replace the incorrect delimiter with a t
Remove the gene name column header, but keep the trailing tab
Create a metadata file following xpressplot standards
Import data

MicroArray Datasets

xpressplot.get_geo (geo_id, output_info=False)

Purpose:
Get sample data and metadata from a GEO database

Parameters:
geo_id: GEO ID for dataset of interest, input is case insensitive (ex: GSE20716)
output_info: Output long-form metadata to txt file if True (default: False)

Returns:
data: Pandas dataframe with data matrix
metadata: Pandas dataframe with metadata

Examples:

```
> import pandas as pd
> import xpressplot as xp
> data, metadata = xp.get_geo('GSE20916')
> data
              GSM523242 GSM523243 GSM523244 GSM523245 ...
1007_s_at     8.98104   8.59941   8.25395   8.72981   ...
1053_at       5.84313   6.59168   8.27881   6.64005   ...
121_at        6.17189   5.73603   5.55673   5.69374   ...
1294_at       6.97009   6.80003   5.56620   7.43816   ...
...           ...       ...       ...       ...       ...
> metadata
    0         1
0   GSM523242 mucosa_normal_colon
1   GSM523243 mucosa_normal_colon
2   GSM523244 mucosa_adenoma
3   GSM523245 colonic_crypt_epithelial_cells_normal_colon
... ...       ...
```

2.4.4 Catenate Raw Counts Files

xpressplot.catenate_files (directory, file_suffix='txt', save_file=None, delimiter='t', drop_rows=0)

Purpose:
Compiles expression counts from multiple files into one table. For example, HTSeq-count outputs each alignment file's counts as a separate count file. This module will collect all single count files and compile them into a single count table.

Assumptions:

- File length of each is the same and ordered the same (same genes in the same order)
- Files to parse are expected to be header-less and column[0] should be gene identifiers and column[1] should be expression values

Parameters:
directory: Path to directory containing raw counts files (only tested currently with HTSeq-count output files)
file_suffix: Common suffix of all count files (default: 'txt'). This feature is useful for modification if there other files in the directory that are not count files, as if they do not contain the same suffix, they will not be used in the function.
save_file: Include if you want the resulting counts table saved for later use (default: None)
delimiter: Delimiter style for expression files, will also output files if saved in this same format
drop_rows: Number of rows to drop from the end of each count file. HTSeq-count provides 5 lines of summary statistics at the end of each file, so for HTSeq-count files, use drop_rows=5

Returns:
count_table: Pandas dataframe with the catenated counts. Samples are along columns, genes are along rows

Examples:

```
> counts = xp.catenate_files(count_dir, file_suffix='counts.txt', drop_rows=5)
> counts
        S1_counts.txt S2_counts.txt S3_counts.txt S4_counts.txt
Gene1   66            59            1             82
Gene2   35            0             7             72
Gene3   20            70            87            78
Gene4   96            7             93            38
...     ...           ...           ...           ...
```

2.4.5 Create Count Table from File List

xpressplot.count_table (file_list, gene_column=0, sample_column=1, sep='t', drop_rows=5)

Purpose:
Collate HTseq counts files (similar to catenate_files(), but input is a file list)

Assumptions:
- No headers are included in the count files

Parameters:
file_list: List of files with the path names appended to each file to be collated into a single count table
gene_column: Column location in all count files of gene names
gene_column: Column location in all count files of samples
sep: Separator of counts files
drop_rows: Number of rows to drop from the end of each count file. HTSeq-count provides 5 lines of summary statistics at the end of each file, so for HTSeq-count files, use drop_rows=5

Returns:
count_table: Pandas dataframe with the catenated counts. Samples are along columns, genes are along rows

2.4.6 Drop Samples

xpressplot.drop_samples (data, ids)

Purpose:
Drop samples by sample IDs – pass in a list of names

Assumptions:
- Dataframe axes have been properly formatted (samples are columns, genes are rows)

Parameters:
data: Dataframe containing expression data
ids: List of sample IDs to remove from the dataframe

Returns:
data: Pandas dataframe with modified data matrix

Examples:

```
> data
              GSM523242 GSM523243 GSM523244 GSM523245 ...
1007_s_at     8.98104   8.59941   8.25395   8.72981   ...
1053_at       5.84313   6.59168   8.27881   6.64005   ...
121_at        6.17189   5.73603   5.55673   5.69374   ...
1294_at       6.97009   6.80003   5.56620   7.43816   ...
...           ...       ...       ...       ...       ...
> data = xp.drop_samples(data, metadata, ['GSM523244'])
> data
              GSM523242 GSM523243 GSM523245 ...
1007_s_at     8.98104   8.59941   8.72981   ...
1053_at       5.84313   6.59168   6.64005   ...
121_at        6.17189   5.73603   5.69374   ...
1294_at       6.97009   6.80003   7.43816   ...
...           ...       ...       ...       ...
```

2.4.7 Drop label

xpressplot.drop_label (data, info, label)

Purpose:
Drop samples by label group name

Assumptions:
- Dataframe axes have been properly formatted (samples are columns, genes are rows)
- Only one string is given to drop per call instance of function

Parameters:
data: Dataframe containing expression data
info: Dataframe containing sample information data
label: Name of sample type to drop (string)

Returns:
data: Pandas dataframe with modified data matrix

Examples:

```
> data
              GSM523242 GSM523243 GSM523244 GSM523245 ...
1007_s_at     8.98104   8.59941   8.25395   8.72981   ...
1053_at       5.84313   6.59168   8.27881   6.64005   ...
121_at        6.17189   5.73603   5.55673   5.69374   ...
1294_at       6.97009   6.80003   5.56620   7.43816   ...
...           ...       ...       ...       ...       ...
> data = xp.drop_label(data, metadata, 'mucosa_adenoma')
```

(continues on next page)

(continued from previous page)

```
> data
              GSM523242 GSM523243 GSM523245 ...
1007_s_at     8.98104   8.59941   8.72981   ...
1053_at       5.84313   6.59168   6.64005   ...
121_at        6.17189   5.73603   5.69374   ...
1294_at       6.97009   6.80003   7.43816   ...
...           ...       ...       ...       ...
```

2.4.8 Keep labels

xpressplot.keep_labels (data, info, label_list=None)

Purpose:
Keep samples by list of label names

Assumptions:
- Dataframe axes have been properly formatted (samples are columns, genes are rows)
- Labels provided are in list format

Parameters:
data: Dataframe containing expression data
info: Dataframe containing sample information data
labels: List of sample types to keep

Returns:
data: Pandas dataframe with modified data matrix

Examples:

```
> data
              GSM523242 GSM523243 GSM523244 GSM523245 ...
1007_s_at     8.98104   8.59941   8.25395   8.72981   ...
1053_at       5.84313   6.59168   8.27881   6.64005   ...
121_at        6.17189   5.73603   5.55673   5.69374   ...
1294_at       6.97009   6.80003   5.56620   7.43816   ...
...           ...       ...       ...       ...       ...
> data = xp.keep_labels(data, metadata, ['mucosa_normal_colon', 'mucosa_adenoma'])
> data
              GSM523242 GSM523243 GSM523244 ...
1007_s_at     8.98104   8.59941   8.25395   ...
1053_at       5.84313   6.59168   8.27881   ...
121_at        6.17189   5.73603   5.55673   ...
1294_at       6.97009   6.80003   5.56620   ...
...           ...       ...       ...       ...
```

2.4.9 Rename dataframe column names

xpressplot.rename_cols (data, converters)

Purpose:
Rename column names using dataframe

Parameters:
data: Dataframe to rename column names
converters: Dataframe where column 0 contains old names and column 1 contains new names

Returns:
data: Pandas dataframe with modified data matrix

Examples:

```
> data
              GSM523242 GSM523243 GSM523244 GSM523245 ...
1007_s_at     8.98104   8.59941   8.25395   8.72981   ...
1053_at       5.84313   6.59168   8.27881   6.64005   ...
121_at        6.17189   5.73603   5.55673   5.69374   ...
1294_at       6.97009   6.80003   5.56620   7.43816   ...
...           ...       ...       ...       ...       ...
> conversion_table
    0         1
0   GSM523242 normal
1   GSM523244 adenoma
2   GSM523245 normal
> data = xp.rename_cols(data, conversion_table)
> data
              normal    GSM523243 adenoma   normal ...
1007_s_at     8.98104   8.59941   8.25395   8.72981   ...
1053_at       5.84313   6.59168   8.27881   6.64005   ...
121_at        6.17189   5.73603   5.55673   5.69374   ...
1294_at       6.97009   6.80003   5.56620   7.43816   ...
...           ...       ...       ...       ...       ...
```

2.4.10 Rename genes with GTF

xpressplot.convert_names_gtf (data, gtf, orig_name_label='gene_id "', orig_name_location=0, new_name_label='gene_name "', new_name_location=1, refill=None, sep='t')

Purpose:
Convert row names (genes) of dataframe using GTF as reference for new name

Important Notes:

- A cursory look at the GTF may be required to determine where in the final field the conversion data lies. Position is relative to delimiter in the final field (usally a ";"), so if the new name is in the third position, new_name_location=2, etc.
- This function is pulling original and new gene name information from any row where the third field is "gene". You can run `cat transcripts.gtf | awk '$3 == "gene"' | less -S` from the command line of your reference file to identify the positions of the required text fields

Parameters:
data: Dataframe to convert rows names
gtf: Path and name of gtf reference file
orig_name_label: Label of original name (usually a "gene_id "')
orig_name_location: Position in last column of GTF where relevant data is found (i.e. 0 would be the first sub-string before the first comma, 3 would be the third sub-string after the second comma before the third comma)
new_name_label: Label of original name (usually "gene_name ")
new_name_location: Position in last column of GTF where relevant data is found (i.e. 0 would be the first sub-string before the first comma, 3 would be the third sub-string after the second comma before the third comma)
refill: In some cases, where common gene names are unavailable, the dataframe will fill the gene name with the improper field of the GTF. In this case, specify this improper string and these values will be replaced with the original name
sep: GTF delimiter (usually tab-delimited)

Returns:
data: Pandas dataframe with modified data matrix

Examples:

```
> data
      gene_names     GSM523242 GSM523243 GSM523244 GSM523245 ...
0     YXZ1034C       8.98104   8.59941   8.25395   8.72981   ...
1     YXA7834D       5.84313   6.59168   8.27881   6.64005   ...
2     YXZ349C        6.17189   5.73603   5.55673   5.69374   ...
3     YXZ1994A       6.97009   6.80003   5.56620   7.43816   ...
...   ...            ...       ...       ...       ...       ...
> data = xp.convert_names_gtf(data, '/path/to/transcripts.gtf', new_name_label='gene_
↪name \"', new_name_location=2)
> data
      gene_names    GSM523242 GSM523243 GSM523244 GSM523245 ...
0     Gene1         8.98104   8.59941   8.25395   8.72981   ...
1     Gene2         5.84313   6.59168   8.27881   6.64005   ...
2     Gene3         6.17189   5.73603   5.55673   5.69374   ...
3     Gene4         6.97009   6.80003   5.56620   7.43816   ...
...   ...           ...       ...       ...       ...       ...
```

2.4.11 Rename dataframe row names

xpressplot.rename_rows (data, converters, label='index')

Purpose:
Rename values in an index (row names) or a column

Parameters:
data: Dataframe to rename rows of a column
converters: Dataframe where column 0 contains old names and column 1 contains new names
label: Name of column to convert names; if 'index' is provided, will rename the index of the dataframe

Returns:

data: Pandas dataframe with modified data matrix

Examples:

```
> data
              GSM523242 GSM523243 GSM523244 GSM523245 ...
1007_s_at     8.98104   8.59941   8.25395   8.72981   ...
1053_at       5.84313   6.59168   8.27881   6.64005   ...
121_at        6.17189   5.73603   5.55673   5.69374   ...
1294_at       6.97009   6.80003   5.56620   7.43816   ...
...           ...       ...       ...       ...       ...
> conversion_table
    0         1
0   1007_s_at Gene1
1   121_at Gene2
> data = xp.rename_rows(data, conversion_table)
> data
              GSM523242 GSM523243 GSM523244 GSM523245 ...
Gene1         8.98104   8.59941   8.25395   8.72981   ...
1053_at       5.84313   6.59168   8.27881   6.64005   ...
Gene2         6.17189   5.73603   5.55673   5.69374   ...
1294_at       6.97009   6.80003   5.56620   7.43816   ...
...           ...       ...       ...       ...       ...
```

```
> data
     gene_names    GSM523242 GSM523243 GSM523244 GSM523245 ...
0    1007_s_at     8.98104   8.59941   8.25395   8.72981   ...
1    1053_at       5.84313   6.59168   8.27881   6.64005   ...
2    121_at        6.17189   5.73603   5.55673   5.69374   ...
3    1294_at       6.97009   6.80003   5.56620   7.43816   ...
...  ...           ...       ...       ...       ...       ...
> conversion_table
    0         1
0   1007_s_at Gene1
1   121_at Gene2
> data = xp.rename_rows(data, conversion_table, label='gene_names')
> data
     gene_names    GSM523242 GSM523243 GSM523244 GSM523245 ...
0    Gene1         8.98104   8.59941   8.25395   8.72981   ...
1    1053_at       5.84313   6.59168   8.27881   6.64005   ...
2    Gene2         6.17189   5.73603   5.55673   5.69374   ...
3    1294_at       6.97009   6.80003   5.56620   7.43816   ...
...  ...           ...       ...       ...       ...       ...
```

2.5 Normalization and Quality Control

2.5.1 RPM (Reads per Million)

xpressplot.rpm (data)

Purpose:
Perform reads per million sample normalization on RNAseq data

Formula:
$RPM_g = \frac{1e6 \cdot r_{ge}}{\sum_{g=1}^{n} r_{ge}}$

Assumptions:

- Dataframe contains raw count data, where samples are along columns and genes across rows

Parameters:
data: Input dataframe with counts values

Returns:
data_rpm: Pandas dataframe with RPM-normalized

Examples:

```
> data
      fGSM523242  fGSM523243  fGSM523244  fGSM523245  fGSM523246
Gene1 66          59          1           82          45
Gene2 35          0           7           72          2
Gene3 20          70          85          78          36
Gene4 96          7           93          38          85
Gene5 73          41          92          77          26
> data = xp.rpm(data)
> data
      fGSM523242  fGSM523243  fGSM523244  fGSM523245  fGSM523246
Gene1 227586.2069 333333.3333 3597.1223   236311.2392 231958.7629
Gene2 120689.6552 0.0000      25179.8561  207492.7954 10309.2784
Gene3 68965.5172  395480.2260 305755.3957 224783.8617 185567.0103
Gene4 331034.4828 39548.0226  334532.3741 109510.0865 438144.3299
Gene5 251724.1379 231638.4181 330935.2518 221902.0173 134020.6186
```

2.5.2 R/FPKM (Reads/Fragments per Kilobase Million per Million Mapped Reads)

xpressplot.r_fpkm (data, gtf, feature_type='exon', identifier='gene_name', sep='t')

Purpose:
Perform reads/fragments per kilobase per million mapped reads sample normalization on RNAseq data

Formulae:
$RPKM_g = \frac{1e9 \cdot r_{ge}}{(\sum_{g=1}^{n} r_{ge}) \cdot l_{ge}}$

$FPKM_g = \frac{1e9 \cdot f_{ge}}{(\sum_{g=1}^{n} f_{ge}) \cdot l_{ge}}$

Assumptions:

- Dataframe contains raw count data, where samples are along columns and genes across rows
- As FPKM was developed for paired-end sequencing, it accounts for two reads being able to map to one fragment. Therefore, input counts should have accounted for this is the counting step of sequence quantification. If specifying a paired-end alignment in XPRESSpipe, this will have been accounted for.

- By default, this will take the longest transcript based on combined exon length. If you prefer to use the cumulative exon length of the Ensembl canonical transcript, you must first curate the GTF using `xpresspipe modifyGTF -g transcripts.gtf -l`
- If you performed isoform quantification and wish to length normalize each isoform, provide `identifier='transcript_id'`
- If you would like to normalize based on CDS length, provide `feature_type='CDS'`

Parameters:
data: Input dataframe with counts values
gtf: GTF reference file path and name
feature_type: Label of feature to use in length normalization
identifier: Label for how to group genes/transcripts. If normalizing for a gene, use `gene_name` or `gene_id`. If performing length normalization for isoforms, provide `transcript_id`
sep: GTF delimiter (usually tab-delimited)

Returns:
data_rpkm: Pandas dataframe with R/FPKM-normalized

Examples:

```
> data
      fGSM523242  fGSM523243  fGSM523244  fGSM523245  fGSM523246
Gene1 66          59          1           82          45
Gene2 35          0           7           72          2
Gene3 20          70          85          78          36
Gene4 96          7           93          38          85
Gene5 73          41          92          77          26
> data = xp.r_fpkm(data, '/path/to/transcripts.gtf')
> data
      fGSM523242  fGSM523243  fGSM523244  fGSM523245  fGSM523246
Gene1 15006.3436  21978.9881  237.1833    15581.6457  15294.6567
Gene2 18516.3632  0.0000      3863.1261   31833.8133  1581.6628
Gene3 1552.2985   8901.5987   6882.0428   5059.5089   4176.8031
Gene4 10220.8992  1221.0702   10328.8988  3381.1932   13527.9835
Gene5 12188.2602  11215.7274  16023.5923  10744.2995  6489.1599
```

2.5.3 TPM (Transcripts per Million)

xpressplot.tpm (data, gtf, feature_type='exon', identifier='gene_name', sep='t')

Purpose:
Perform transcripts per million sample normalization on RNAseq data

Formula:
$$TPM_g = \frac{1e6 \cdot r_{ge}}{(\sum_{g=1}^{n}(\frac{1e3 \cdot r_{ge}}{l_{ge}})) \cdot l_{ge}}$$

Assumptions:
- Dataframe contains raw count data, where samples are along columns and genes across rows
- By default, this will take the longest transcript based on combined exon length. If you prefer to use the

cumulative exon length of the Ensembl canonical transcript, you must first curate the GTF using `xpresspipe modifyGTF -g transcripts.gtf -l`
- If you performed isoform quantification and wish to length normalize each isoform, provide `identifier='transcript_id'`
- If you would like to normalize based on CDS length, provide `feature_type='CDS'`

Parameters:
data: Input dataframe with counts values
gtf: GTF reference file path and name
feature_type: Label of feature to use in length normalization
identifier: Label for how to group genes/transcripts. If normalizing for a gene, use `gene_name` or `gene_id`. If performing length normalization for isoforms, provide `transcript_id`
sep: GTF delimiter (usually tab-delimited)

Returns:
data_tpm: Pandas dataframe with TPM-normalized

2.5.4 Batch Normalize

xpressplot.batch_normalize (input_file, batch_file)

Purpose:
Control for batch effects between datasets

Assumptions:
- Requires a properly formatted dataframe for xpressplot usage where samples are normalized previously if desired
- Requires a properly formatted dataframe complying to SVA COMBAT info file (see example below)
- R is installed on your machine and is in your $PATH
- All input files are tab-delimited (with .txt or .tsv suffix)

Parameters:
input_file: Input dataframe file with values (can be normalized or unnormalized)
batch_file: Input dataframe containing batch effect information, column naming convention must be followed and *is* case-sensitive

Examples:

```
> data = pd.read_csv('/path/to/expression.tsv', index_col=0)
> data
                s1_rpf  s1_rna  s2_rpf  s2_rna
ENSG00000227232 66.34   59.13   1.90    82.49
ENSG00000240361 35.73   0.00    7.38    72.94
ENSG00000238009 20.02   70.21   85.10   78.87
ENSG00000241860 96.23   7.49    93.49   38.39
ENSG00000187634 73.91   41.28   92.27   77.93
> batch = pd.read_csv('/path/to/batch_info.tsv', index_col=0)
```

(continues on next page)

(continued from previous page)

```
> batch
  Sample  Batch
0 s1_rpf  batch1
1 s1_rna  batch2
2 s2_rpf  batch1
3 s2_rna  batch2
> xp.batch_normalize('/path/to/expression.tsv', '/path/to/batch_info.tsv')
```

2.5.5 Clean Data

xpressplot.clean_df (data, axis=0)

Purpose:
Cleans NULL values from axis and clears duplicate indices

Assumptions:
- Requires a properly formatted dataframe for xpressplot usage

Parameters:
data: Input dataframe file with values (can be normalized or unnormalized)
axis: Axis to clean NaN values from (default: 0, which corresponds to rows)

Returns:
data_clean: Cleaned pandas dataframe

Examples:

```
> data
                s1_rpf  s1_rna  s2_rpf  s2_rna
ENSG00000227232 66.34   59.13   1.90    NA
ENSG00000240361 35.73   0.00    7.38    72.94
Gene2           20.02   70.21   85.10   78.87
Gene2           96.23   7.49    93.49   38.39
ENSG00000187634 73.91   NA      92.27   77.93
> data = xp.clean_df(data)
> data
                s1_rpf  s1_rna  s2_rpf  s2_rna
ENSG00000240361 35.73   0.00    7.38    72.94
```

2.5.6 Set Gene Threshold

xpressplot.threshold (data, minimum=None, maximum=None)

Purpose:
Cleans gene axis (assumed to by rows) of genes containing values below or above user-determined thresholds

Assumptions:

- Requires a properly formatted dataframe for xpressplot usage

Parameters:
data: Input dataframe file with values (can be normalized or unnormalized)
minimum: Minimum value all samples need of a given gene to avoid dropping across all samples
maximum: Maximum value all samples can have of a given gene to avoid dropping across all samples

Returns:
data_clean: Cleaned pandas dataframe

Examples:

```
> data
                s1_rpf  s1_rna  s2_rpf  s2_rna
ENSG00000227232 66.34   59.13   1.90    82.49
ENSG00000240361 35.73   0.00    7.38    72.94
ENSG00000238009 20.02   70.21   85.10   78.87
ENSG00000241860 96.23   7.49    93.49   38.39
ENSG00000187634 73.91   41.28   92.27   77.93
> data = xp.threshold(data, minimum=5)
> data
                s1_rpf  s1_rna  s2_rpf  s2_rna
ENSG00000238009 20.02   70.21   85.10   78.87
ENSG00000241860 96.23   7.49    93.49   38.39
ENSG00000187634 73.91   41.28   92.27   77.93
```

2.5.7 Prepare xpressplot Dataset

xpressplot.prep_data (data, info, gene_scale=True, print_means=False)

Purpose:
Prepare dataframe for downstream analyses

Assumptions:
- Requires a properly formatted dataframe for xpressplot usage (genes as rows, samples as columns)
- Requires properly formatted xpressplot metadata dataframe

Parameters:
data: xpressplot formatted dataframe of expression values
info: xpressplot formatted sample info dataframe
gene_scale: Scale genes (rows) of data
print_means: Print means for each sample verification

Returns:
data_normalized: Normalized pandas dataframe
data_labeled: Labeled pandas dataframe

2.5.8 Check Sample Expression Distributions

xpressplot.check_samples (data)

Purpose:
Visualize gene expression distributions on a sample-by-sample basis

Assumptions:
- Requires a properly formatted dataframe for xpressplot usage

Parameters:
data: Input dataframe file with values (can be normalized or unnormalized)

Returns:
Boxplot with samples on the x-axis and lump expression distributions for all genes in that sample

Examples:

```
> xp.check_samples(data)
```

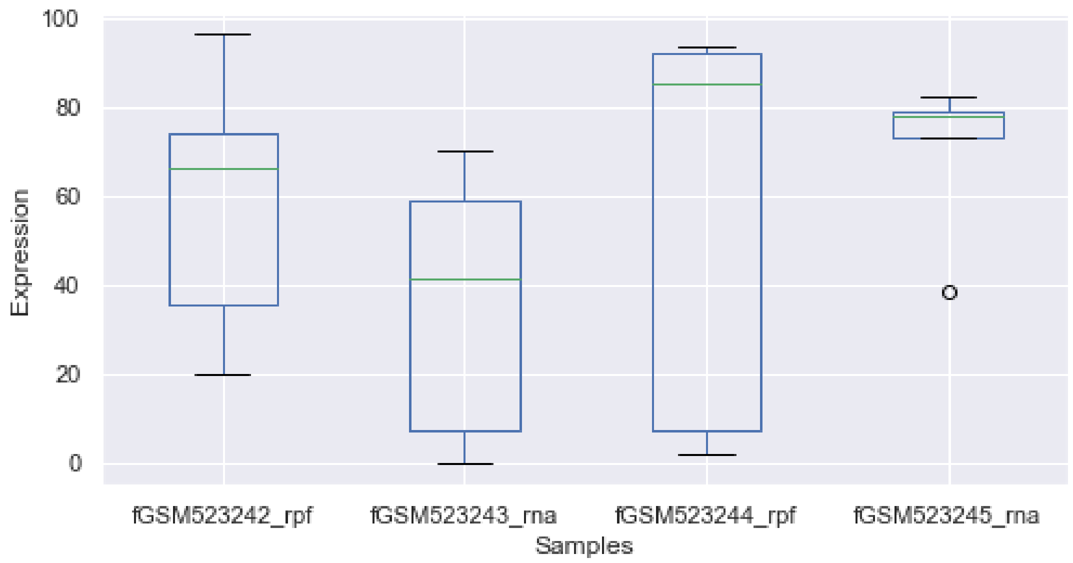

2.5.9 Microarray Probe Collapse

xpressplot.probe_collapse (data, reference, gene_list=None, no_multimappers=True)

Purpose:
Remove multimapping probes and collapse probes mapping to the same gene by averaging the values for those probes per sample

Assumptions:

- Requires a properly formatted dataframe for xpressplot usage
- Assumes GPL .txt file from NCBI is tab delimited

Parameters:
data: Input dataframe file with values (can be normalized or unnormalized)

Returns:
data_collapsed: Pandas dataframe file probes collapsed and the corresponding gene names listed

Examples:

```
> data
            fGSM523242  fGSM523243  fGSM523244  fGSM523245  fGSM523246
1007_s_at   66          59          1           82          45
1053_at     35          0           7           72          2
121_at      20          70          85          78          36
218024_at   96          7           93          38          85
240362_at   73          41          92          77          26
> probe_collapse = xp.probe_collapse(probe_test, '/path/to/gpl_ref.txt')
> probe_collapse
      fGSM523242  fGSM523243  fGSM523244  fGSM523245  fGSM523246
MPC1  84.5        24.0        92.5        57.5        55.5
PAX8  20.0        70.0        85.0        78.0        36.0
RFC2  35.0        0.0         7.0         72.0        2.0
```

```
> data
            fGSM523242  fGSM523243  fGSM523244  fGSM523245  fGSM523246
1007_s_at   66          59          1           82          45
1053_at     35          0           7           72          2
121_at      20          70          85          78          36
218024_at   96          7           93          38          85
240362_at   73          41          92          77          26
> probe_collapse = xp.probe_collapse(probe_test, '/path/to/gpl_ref.txt', no_
↪multimappers=False))
> probe_collapse
                  fGSM523242  fGSM523243  fGSM523244  fGSM523245  fGSM523246
DDR1 /// MIR4640  66.0        59.0        1.0         82.0        45.0
MPC1              84.5        24.0        92.5        57.5        55.5
PAX8              20.0        70.0        85.0        78.0        36.0
RFC2              35.0        0.0         7.0         72.0        2.0
```

2.6 Analysis

The following commands rely heavily on the matplotlib (DOI:10.5281/zenodo.2577644) and seaborn (DOI:10.5281/zenodo.883859) libraries, but have been modified in many cases for ease of plotting given the formatting of xpressplot datasets.

2.6.1 Formatting Notes

Sample Color Palette

A dictionary of experiment groups with corresponding RGB values for colors (use of common color names is not currently tested)

Examples:

```
sample_colors = {'Adenocarcinoma': (0.5725490196078431, 0.5843137254901961, 0.
↪5686274509803921),
                 'Adenoma': (0.8705882352941177, 0.5607843137254902, 0.0196078431372549),
                 'Normal': (0.00784313725490196, 0.6196078431372549, 0.
↪45098039215686275)}
```

2.6.2 Single-gene Analysis

xpressplot.gene_overview (data, info, gene_name, palette, order=None, grid=False, whitegrid=False, save_fig=None, dpi=600, bbox_inches='tight')

Purpose:
Create a boxplot with overlaid swarmplot for each experiment group for a particular gene

Assumptions:

- Dataframe and metadata are properly formatted for use with xpressplot

Parameters:
data: xpressplot-formatted dataframe (Required)
info: xpressplot formatted sample info dataframe (Required)
gene_name: Name of gene to plot (Required)
palette: Dictionary of matplotlib compatible colors for samples (Required)
order: List of experiment groups in order to plot (Default: None)
grid: Set to True to add gridlines (default: False)
whitegrid: Set to True to create white background in figure (default: Grey-scale)
save_fig: Full file path, name, and extension for file output (default: None)
dpi: Set DPI for figure output (default: 600)
bbox_inches: Matplotlib bbox_inches argument (default: 'tight'; useful for saving images and preventing text cut-off)

Examples:

```
>>> xp.gene_overview(data, metadata, gene_name='SEC62', palette=sample_colors,
    order=['Normal','Adenoma','Adenocarcinoma'])
```

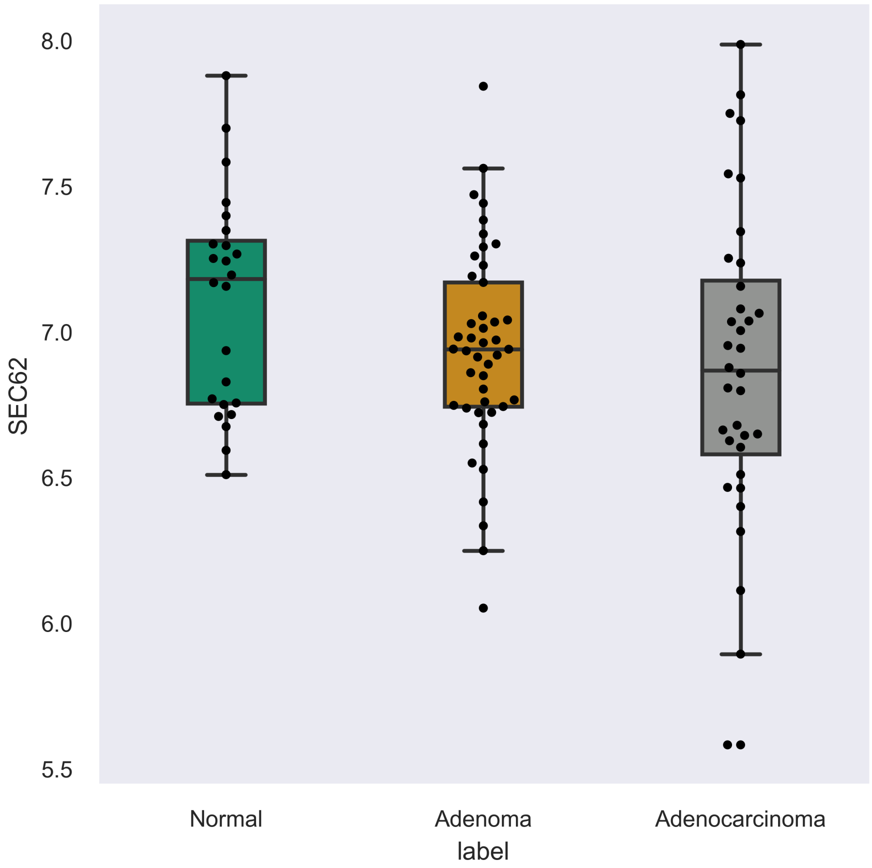


```
>>> xp.gene_overview(data, metadata, 'CCL5', sample_colors, grid=True, whitegrid=True)
```

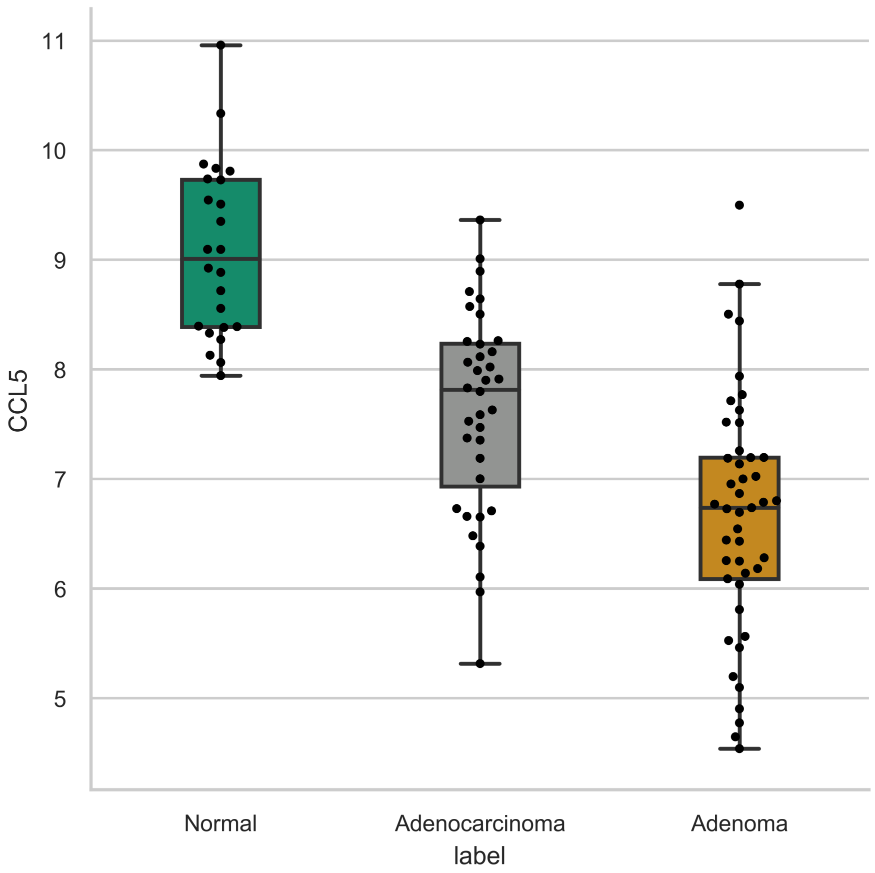

2.6.3 Multi-gene Analysis

xpressplot.multigene_overview (data, info, palette=None, gene_list=None, order=None, scale=None, title=None, grid=False, whitegrid=False, save_fig=None, dpi=600, bbox_inches='tight')

Purpose:
Create violin plots of a subset of gene expressions or total gene expression by experiment group

Assumptions:
- Dataframe and metadata are properly formatted for use with xpressplot

Parameters:
data: xpressplot-formatted dataframe (Required)
info: xpressplot formatted sample info dataframe (Required)
palette: Dictionary of matplotlib compatible colors for samples (Default: None)
gene_list: List of genes to plot (default: None; plots total gene expression for experiment group)
order: List of experiment groups in order to plot (Default: None)
scale: Seaborn violinplot scale argument (default: 'area')
title: Plot title (default: None)
grid: Set to True to add gridlines (default: False)
whitegrid: Set to True to create white background in figure (default: Grey-scale)

save_fig: Full file path, name, and extension for file output (default: None)

dpi: Set DPI for figure output (default: 600)

bbox_inches: Matplotlib bbox_inches argument (default: 'tight'; useful for saving images and preventing text cut-off)

Examples:

```
>>> xp.multigene_overview(data, metadata, palette=sample_colors,
    gene_list=['SEC62','CCL5','STX6'])
```

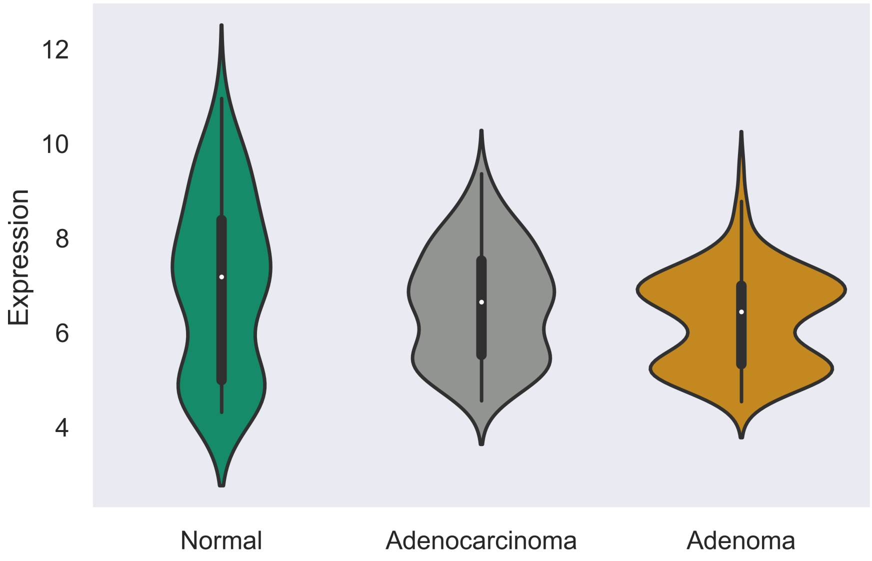


```
>>> xp.gene_overview(data, metadata, palette=sample_colors, gene_list=['STX6'],
    order=['Normal','Adenoma','Adenocarcinoma'])
```

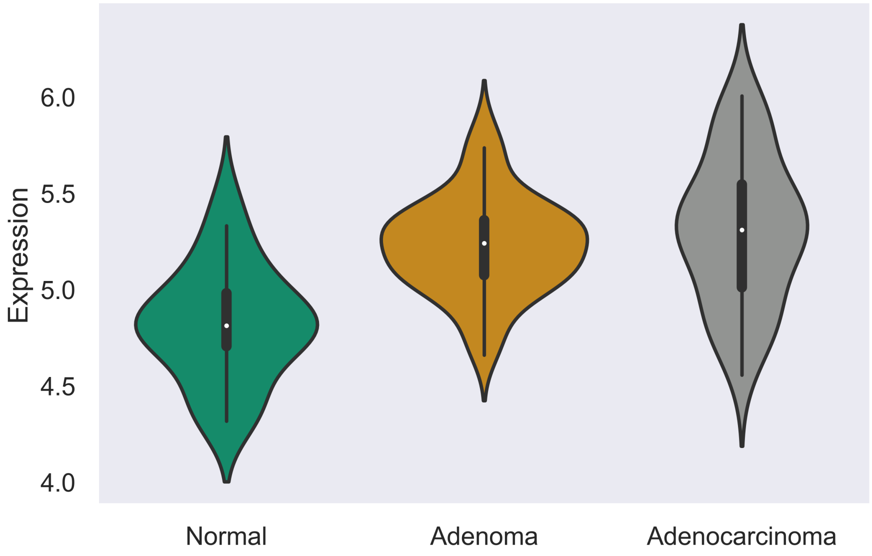

2.6.4 Heatmap

xpressplot.heatmap (data, info, sample_palette=None, gene_info=None, gene_palette=None, gene_list=None, col_cluster=True, row_cluster=False, metric='euclidean', method='centroid', font_scale=0.8, cmap=jakes_cmap, center=0, xticklabels=True, yticklabels=True, linewidths=0, linecolor='#DCDCDC', cbar_kws=None, figsize=(16,6.5), save_fig=None, dpi=600, bbox_inches='tight')

Purpose:
Create clustered heatmaps for gene expression dataframe

Assumptions:
- Dataframe and metadata are properly formatted for use with xpressplot

Parameters:
data: xpressplot-formatted dataframe (Required)
info: xpressplot formatted sample info dataframe (Required)
sample_palette: Dictionary of matplotlib compatible colors for samples (Default: None)
gene_info: xpressplot formatted metadata matrix for genes (column0) and gene groups (column1)
gene_palette: Dictionary of labels and colors for plotting, or valid seaborns clustermap col_colors option
gene_list: List of genes to plot (default: None; plots total gene expression for experiment group)
col_cluster: Cluster columns/samples (default: True)
row_cluster: Cluster rows/genes (default: False)
metric: Seaborn clustermap argument (default: 'euclidean')
method: Seaborn clustermap argument (default: 'centroid')
font_scale: Aspect by which to scale text (default: 0.8)
cmap: Matplotlib colorbar valid entry (default: jakes_cmap; a color-blind friendly color palette)
center: Value at which to center the color scale (default: 0)
xticklabels: Include x-axis labels (default: True)
yticklabels: Include y-axis labels (default: True)
linewidths: Thickness of grid lines (default: 0; no grid-lines printed)
linecolor: Grid line color (default: '#DCDCDC'; or white)
cbar_kw: Matplotlib colorbar additional arguments (default: None)
figsize: Figure size tuple; width, height (default: (16,6.5))
save_fig: Full file path, name, and extension for file output (default: None)
dpi: Set DPI for figure output (default: 600)
bbox_inches: Matplotlib bbox_inches argument (default: 'tight'; useful for saving images and preventing text cut-off)

Examples:

```
>>> xp.heatmap(data, metadata, sample_palette=sample_colors, gene_list=['SEC62','STX6
↪','CCL5'],
    cbar_kws={'label':'z-score'}, figsize=(20,2))
```

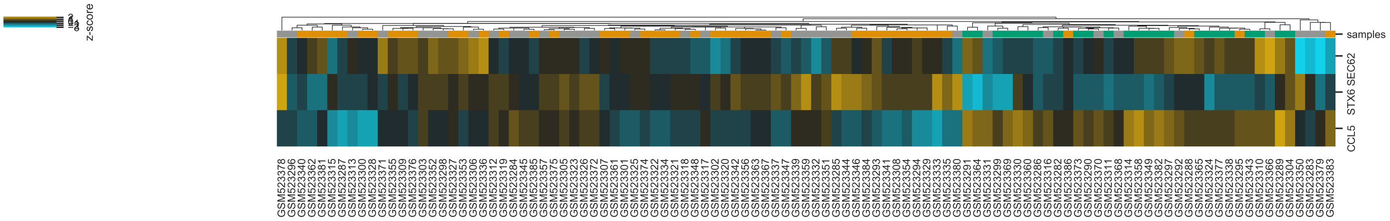


```
>>> xp.heatmap(data, metadata, sample_palette=sample_colors, gene_palette=gene_colors,
    gene_info=gene_metadata, gene_list=['SEC62','STX6','CCL5'], figsize=(20,2),
    row_cluster=True)
```

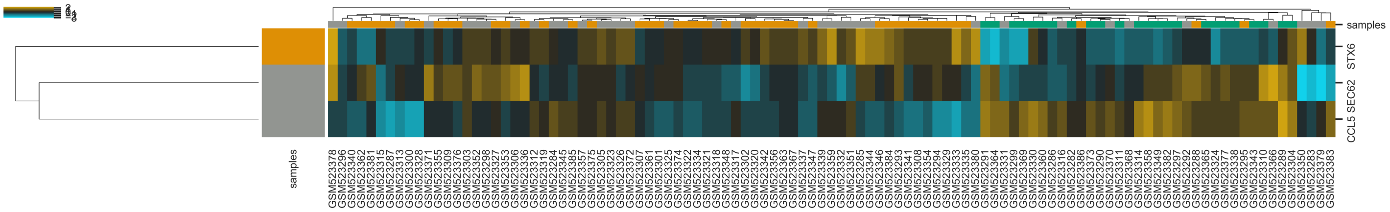


```
>>> xp.heatmap(data, metadata, sample_palette=sample_colors, xticklabels=True, linewidths=.5,
    linecolor='black', gene_list=['SEC62','STX6','CCL5'], figsize=(20,2))
```

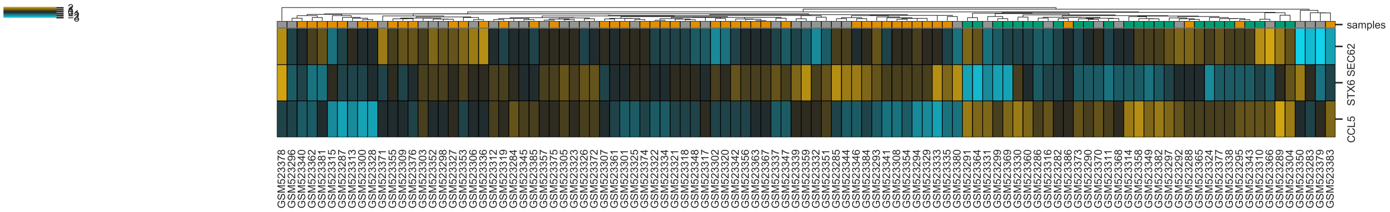

2.6.5 Scatterplot

xpressplot.scatter (data, info, x, y, palette=None, add_linreg=False, order_legend=None, title=None, alpha=1, highlight_points=None, highlight_color='DarkRed', highlight_names=None, alpha_highlights=1, size=30, y_threshold=None, x_threshold=None, threshold_color='b', label_points=None, grid=False, whitegrid=False, save_fig=None, dpi=600, bbox_inches='tight')

Purpose:
Create scatterplot with the option to include a linear least-squares regression fit of the data

Assumptions:
- Dataframe and metadata are properly formatted for use with xpressplot

Parameters:
data: xpressplot-formatted dataframe (Required)
info: xpressplot formatted sample info dataframe (Required)
x: X-axis gene or other metric (Required)
y: Y-axis gene or other metric (Required)
palette: Dictionary of matplotlib compatible colors for samples (Default: None)
add_linreg: Add a linear least-squares regression line (default: False)

order_legend: List of experiment groups in order to display on legend (Default: None)
title: Plot title (default: None)
alpha: Opacity percentage for scatter plot
highlight_points: List of indices to highlight on scatterplot (if desired to plot multiple sets in different colors, lists of lists can be provided)
highlight_color: Color or ordered list of colors to plot highlighted points (if multiple lists are being highlighted, pass colors in same order as a list)
highlight_names: Ordered list of names to use in legend (must follow order provided for highlight_points and highlight_color)
alpha_highlights: Opacity percentage for highlighted elements of scatter plot
size: Marker size
y_threshold: Include a y-axis threshold dotted line (default: None). If a list is provided, each will be plotted
x_threshold: Include a x-axis threshold dotted line (default: None). If a list is provided, each will be plotted
threshold_color: Threshold line color (default: 'b'; black)
label_points: A dictionary where keys are labels and values are a two-element list as [x-coordinate, y-coordinate]
grid: Set to True to add gridlines (default: False)
whitegrid: Set to True to create white background in figure (default: Grey-scale)
save_fig: Full file path, name, and extension for file output (default: None)
dpi: Set DPI for figure output (default: 600)
bbox_inches: Matplotlib bbox_inches argument (default: 'tight'; useful for saving images and preventing text cut-off)

Examples:

```
>>> xp.scatter(data, metadata, 'SEC62', 'STX6', palette=geo_colors, add_linreg=True,
    order_legend=[1,3,2], alpha=.7)
```

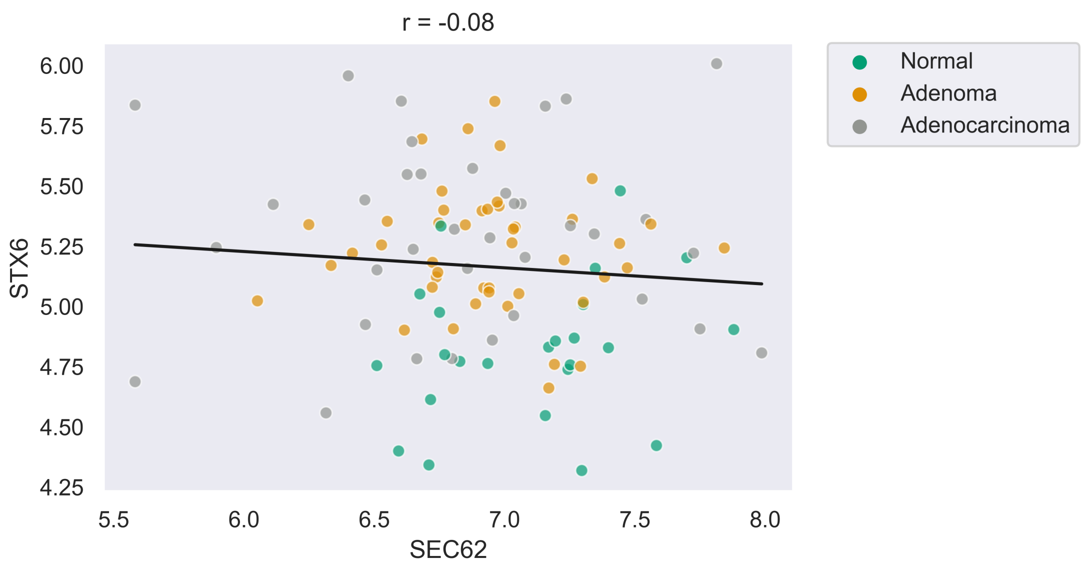


```
>>> xp.scatter(data, metadata, 'SEC62', 'STX6', palette=geo_colors, add_linreg=False,␣
↪alpha=.7)
```

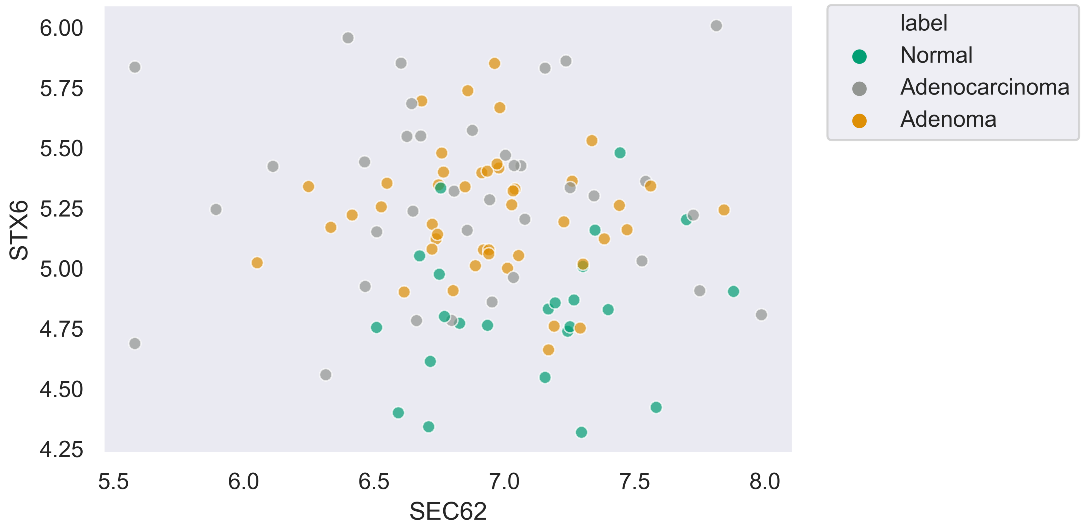


```
>>> xp.scatter(data, metadata, 'SEC62', 'STX6', palette=geo_colors, add_linreg=True,␣
↪alpha=.2,
    title='this is a title', y_threshold=5, x_threshold=[7])
```

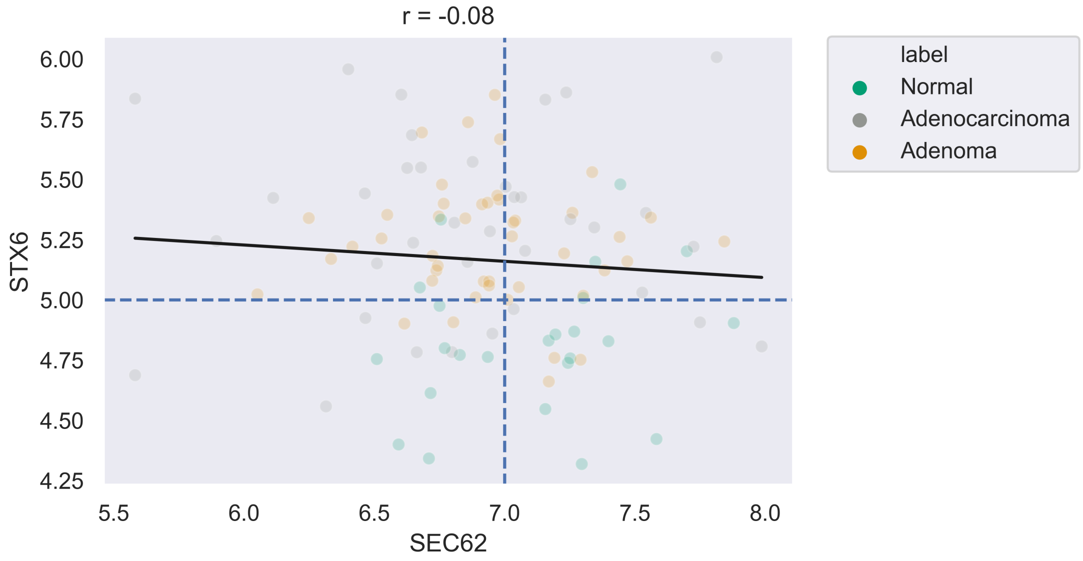

2.6.6 RNA Volcano Plot

xpressplot.rna_volcano (file, order_legend=None, title=None, alpha=1, highlight_points=None, highlight_color='DarkRed', highlight_names=None, alpha_highlights=1, size=30, y_threshold=None, x_threshold=None, threshold_color='b', label_points=None, grid=False, whitegrid=False, interactive=False, save_fig=None, dpi=600, bbox_inches='tight')

Purpose:
Create volcano plot with non-normally distributed data (RNA-seq). See `Volcano Plot` for examples.

Assumptions:

- file is a DESeq2-output table
- Note: Many of the options will be non-functional when using interactive mode

file: Path and file name to DESeq2-output table
order_legend: List of experiment groups in order to display on legend (Default: None)
title: Plot title (default: None)
alpha: Opacity percentage for scatter plot
highlight_points: List of indices to highlight on scatterplot (if desired to plot multiple sets in different colors, lists of lists can be provided)
highlight_color: Color or ordered list of colors to plot highlighted points (if multiple lists are being highlighted, pass colors in same order as a list)
highlight_names: Ordered list of names to use in legend (must follow order provided for highlight_points and highlight_color). Must use if highlighting points.
alpha_highlights: Opacity percentage for highlighted elements of scatter plot
size: Marker size
y_threshold: Include a y-axis threshold dotted line (default: None). If a list is provided, each will be plotted
x_threshold: Include a x-axis threshold dotted line (default: None). If a list is provided, each will be plotted
threshold_color: Threshold line color (default: 'b'; black)
label_points: A dictionary where keys are labels and values are a two-element list as [x-coordinate, y-coordinate]
grid: Set to True to add gridlines (default: False)
whitegrid: Set to True to create white background in figure (default: Grey-scale)
figsize: Set figure size dimensions
interactive: Set as True to create interactive scatter plot (if using this option and saving the output, be sure to include a `html` suffix in the file name)
save_fig: Full file path, name, and extension for file output (default: None)
dpi: Set DPI for figure output (default: 600)
bbox_inches: Matplotlib bbox_inches argument (default: 'tight'; useful for saving images and preventing text cut-off)

2.6.7 Volcano Plot

xpressplot.volcano (data, info, label_comp, label_base, order_legend=None, title=None, alpha=1, highlight_points=None, highlight_color='DarkRed', highlight_names=None, alpha_highlights=1, size=30, y_threshold=None, x_threshold=None, threshold_color='b', save_threshold_hits=None, save_threshold_hits_delimiter=',', label_points=None, grid=False, whitegrid=False, return_data=False, figsize=(10,10), interactive=False, save_fig=None, dpi=600, bbox_inches='tight')

Purpose:
Create volcano plot with normally distributed data

Assumptions:
- Dataframe and metadata are properly formatted for use with xpressplot
- Note: Many of the options will be non-functional when using interactive mode

Parameters:
data: xpressplot-formatted dataframe, sample normalized (Required)
info: xpressplot formatted sample info dataframe (Required)
label_comp: Experiment group name to act as comparison group (Required)

label_base: Experiment group name to act as base group (Required)
order_legend: List of experiment groups in order to display on legend (Default: None)
title: Plot title (default: None)
alpha: Opacity percentage for scatter plot
highlight_points: List of indices to highlight on scatterplot (if desired to plot multiple sets in different colors, lists of lists can be provided)
highlight_color: Color or ordered list of colors to plot highlighted points (if multiple lists are being highlighted, pass colors in same order as a list)
highlight_names: Ordered list of names to use in legend (must follow order provided for highlight_points and highlight_color). Must use if highlighting points.
alpha_highlights: Opacity percentage for highlighted elements of scatter plot
size: Marker size
y_threshold: Include a y-axis threshold dotted line (default: None). If a list is provided, each will be plotted
x_threshold: Include a x-axis threshold dotted line (default: None). If a list is provided, each will be plotted
threshold_color: Threshold line color (default: 'b'; black)
save_threshold_hits: Include path and filename to save points out of bounds of the threshold points (greater than the Y-threshold, and outside of the X-threshold range)
save_threshold_hits_delimiter: Delimiter to use for saving threshold hits (default: ','; .csv)
label_points: A dictionary where keys are labels and values are a two-element list as [x-coordinate, y-coordinate]
grid: Set to True to add gridlines (default: False)
whitegrid: Set to True to create white background in figure (default: Grey-scale)
return_data: Set as True to return dataframe with log2 Fold Changes and -log10 P-values added
figsize: Set figure size dimensions
interactive: Set as True to create interactive scatter plot (if using this option and saving the output, be sure to include a `html` suffix in the file name)
save_fig: Full file path, name, and extension for file output (default: None)
dpi: Set DPI for figure output (default: 600)
bbox_inches: Matplotlib bbox_inches argument (default: 'tight'; useful for saving images and preventing text cut-off)

Examples:

```
>>> xp.volcano(data, metadata, 'Adenoma', 'Normal', highlight_points=['STX6','SCARB1',
↪'CCL5'])
```

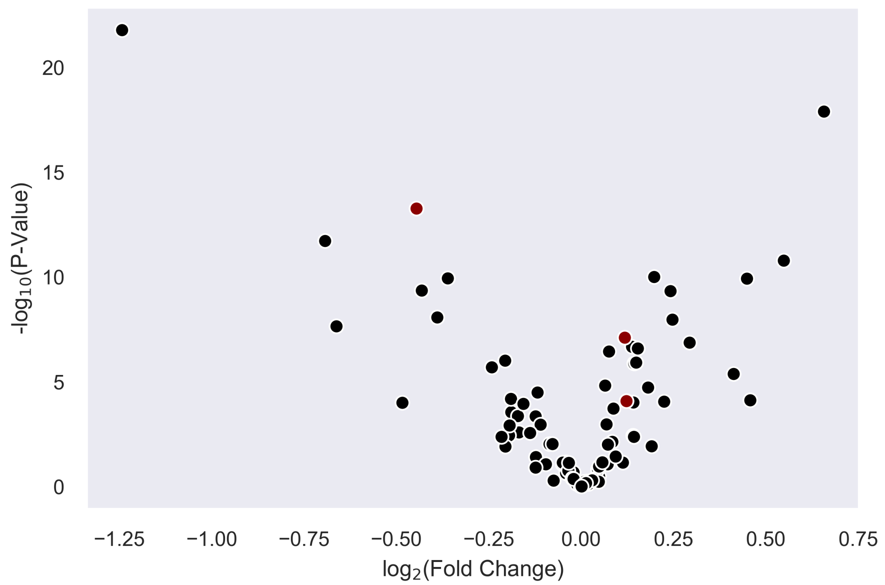


```
>>> xp.volcano(data, metadata, 'Adenoma', 'Normal', highlight_points=['STX6','SCARB1',
↪'CCL5'],
    y_threshold=2, x_threshold=[-1,1], save_threshold_hits=save_threshold)
```

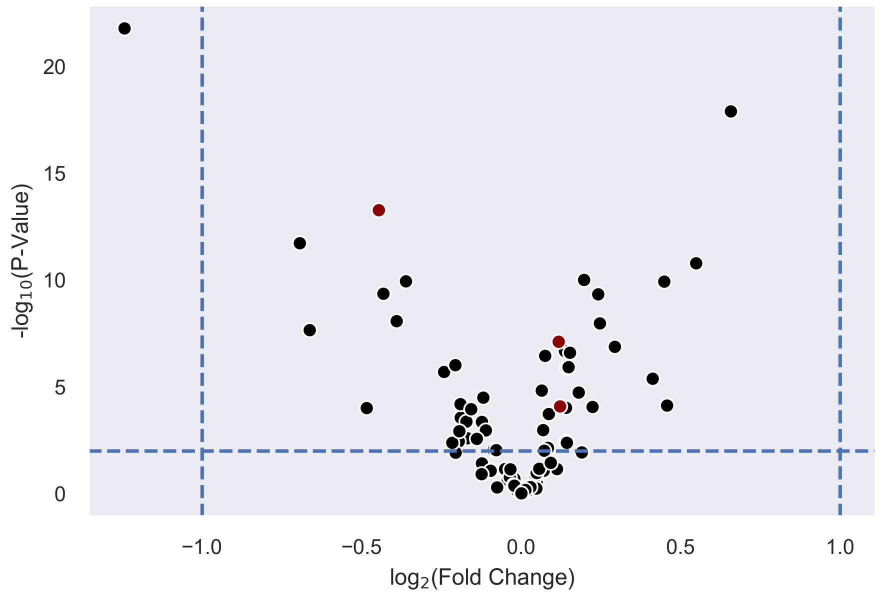


```
>>> xp.volcano(data, metadata, 'Adenoma', 'Normal', highlight_points=[['STX6','SCARB1
↪','CCL5'],['BEST4']],
    highlight_color=['blue','red'], alpha=.3, y_threshold=2, x_threshold=[-1,1],
    label_points={'BEST4':[-1.24288077425345,21.782377963035827]})
```

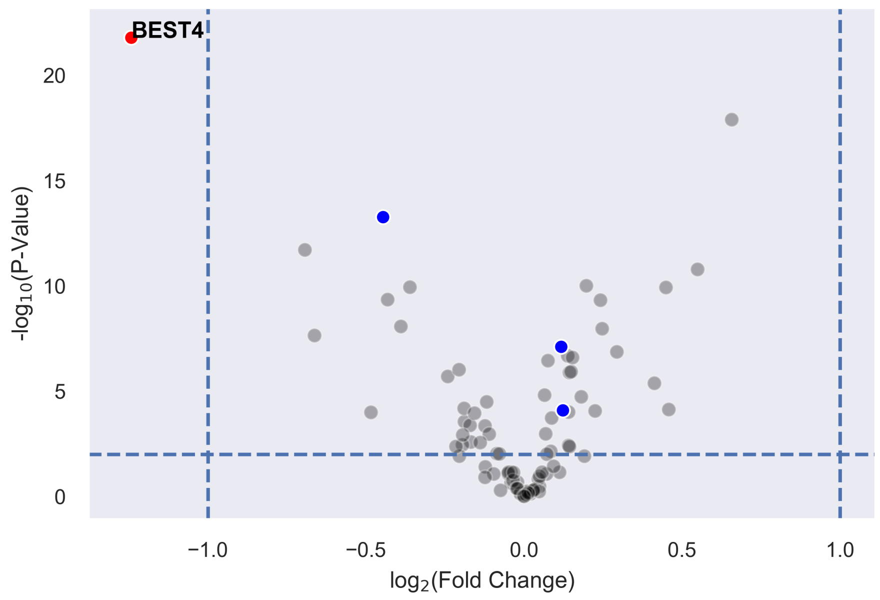

2.6.8 Linear Regression

xpressplot.linreg (data, gene_name, save_file, delimiter=',')

Purpose:
Calculate r, r^2 values, and p-values for every gene against target gene for given dataset

Assumptions:
- Dataframe is properly formatted for use with xpressplot

Parameters:
data: xpressplot-formatted dataframe, sample normalized (Required)
gene_name: Target gene name to run genome-wide comparisons against
save_file: Full file path, name, and extension for file output (default: None)
delimiter: Field separator for output file (default: ',')

Examples:

```
>>> xp.linreg(data, 'STX6', 'path/to/output.csv', delimiter=',')
```

2.6.9 Jointplot

xpressplot.jointplot (data, info, x, y, kind='reg', palette=None, order=None, title_pad=0, title_pos='right', grid=False, whitegrid=False, save_fig=None, dpi=600, bbox_inches='tight')

Purpose:
Create linear regression scatterplot that displays r value, confidence, and density distributions for axes

Assumptions:

- Dataframe and metadata are properly formatted for use with xpressplot

Parameters:

data: xpressplot-formatted dataframe (Required)
info: xpressplot formatted sample info dataframe (Required)
x: X-axis gene or other metric (Required)
y: Y-axis gene or other metric (Required)
kind: Type of plot to create from the seaborns jointplot function (default: 'reg'; linear regression)
palette: Dictionary of matplotlib compatible colors for samples (Default: None)
order: List of experiment groups in order to display on legend (Default: None)
title_pad: Amount of padding to give title from default position (default: 0)
title_pos: Title position (default: 'right'; other options: 'center', 'left')
grid: Set to True to add gridlines (default: False)
whitegrid: Set to True to create white background in figure (default: Grey-scale)
save_fig: Full file path, name, and extension for file output (default: None)
dpi: Set DPI for figure output (default: 600)
bbox_inches: Matplotlib bbox_inches argument (default: 'tight'; useful for saving images and preventing text cut-off)

Examples:

```
>>> xp.jointplot(geo_labeled, meta, 'STX6', 'STX6', kind='reg')
```

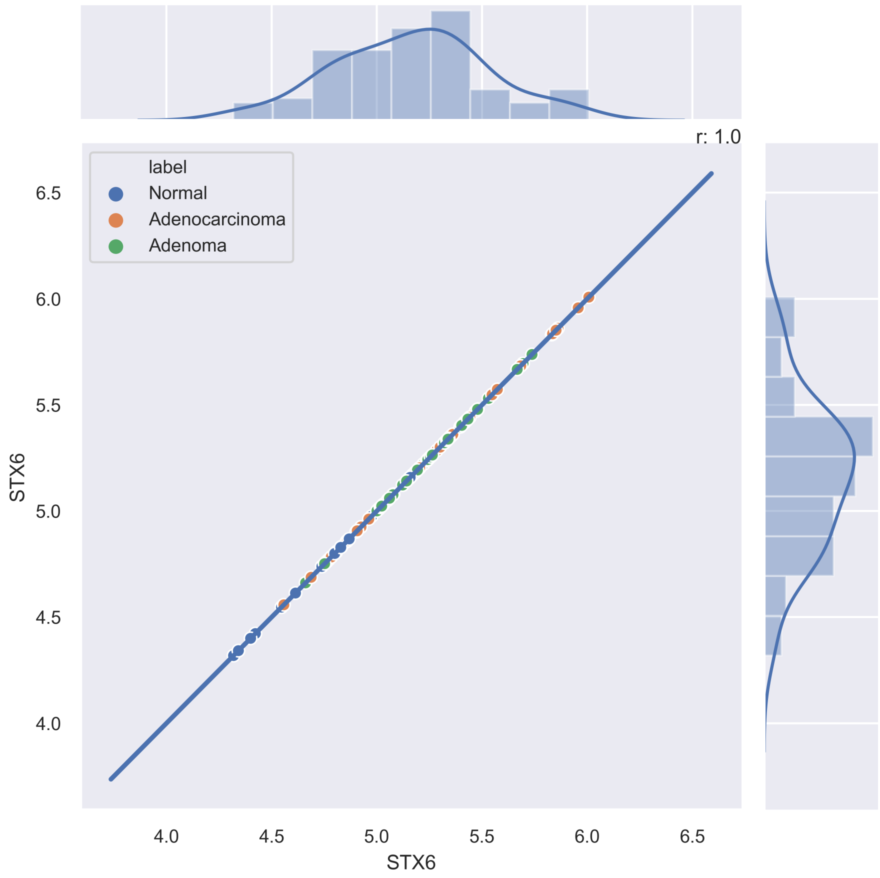


```
>>> xp.jointplot(geo_labeled, meta, 'STX6', 'CCL5', kind='reg', palette=geo_colors,
    order=['Normal','Adenoma','Adenocarcinoma'], title_pad=-305, title_pos='center')
```

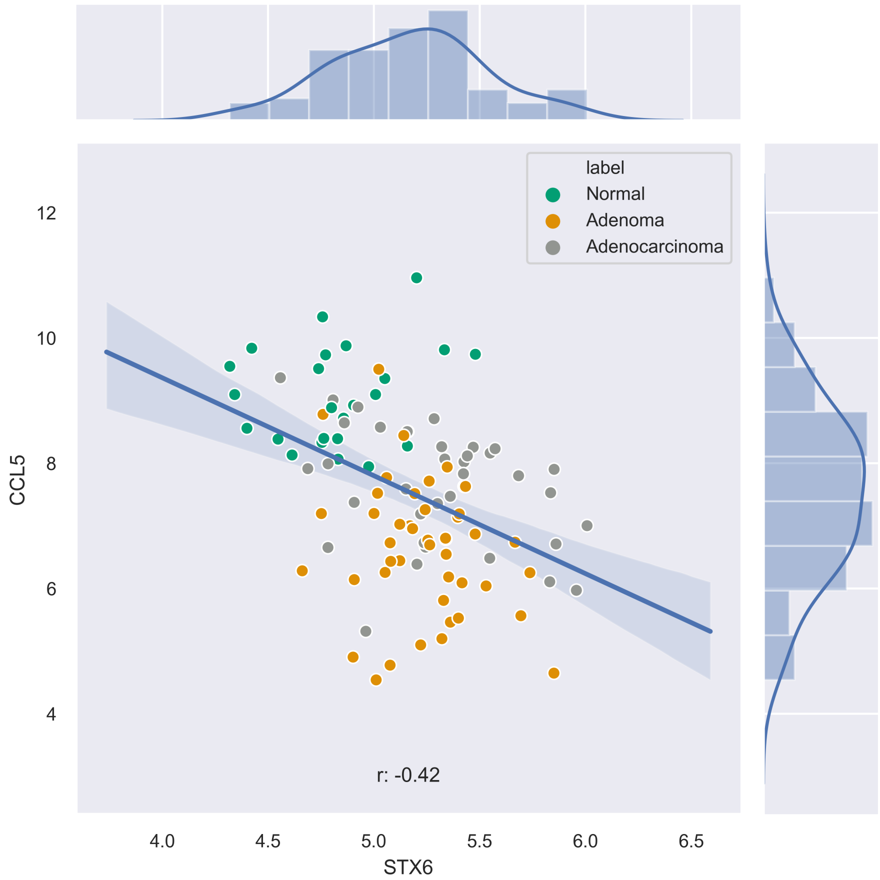


```
>>> xp.jointplot(geo_labeled, meta, 'STX6', 'CCL5', kind='kde', palette=geo_colors,
    order=['Normal','Adenoma','Adenocarcinoma'])
```

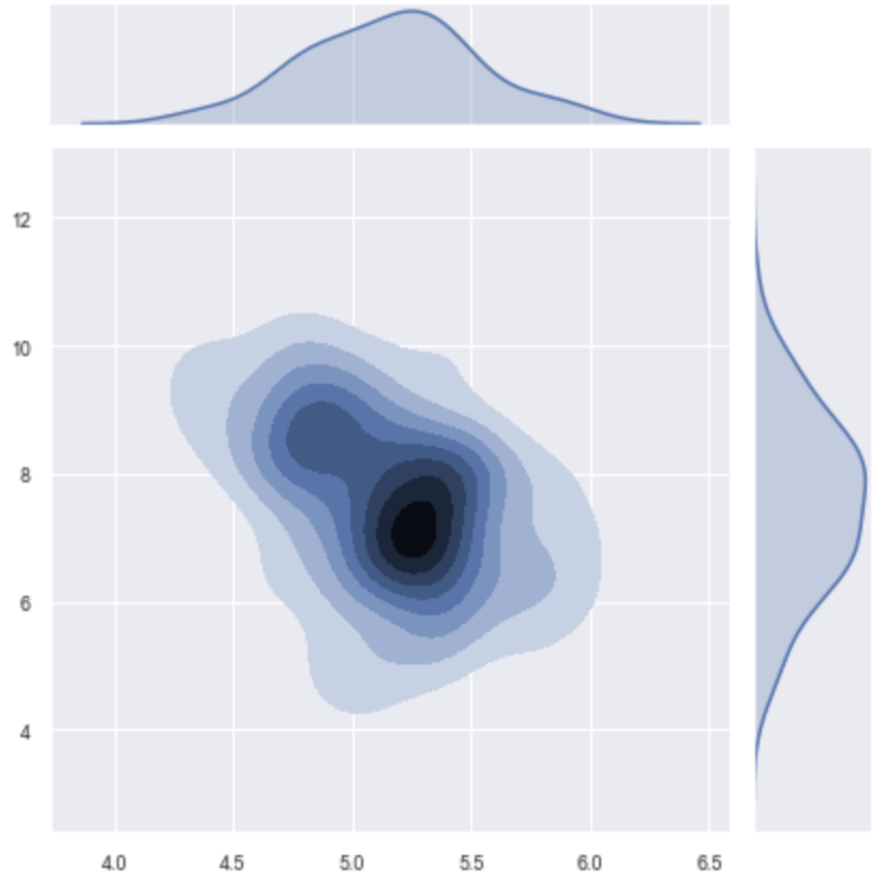

2.6.10 PCA (2-D, 3-D, Interactive)

xpressplot.pca (data, info, palette, grouping='samples', gene_list=None, gene_labels=False, _3d_pca=False, principle_components=[1,2], n_components=10, ci=2, scree_only=False, save_scree=False, size=30, order_legend=None, title=None, fig_size=(10,10), grid=False, whitegrid=False, save_fig=None, dpi=600, bbox_inches='tight', return_pca=False, plotly_login=None)

Purpose:
Plot a 2-D PCA with confidence intervals or a 3-D PCA with no confidence intervals

Assumptions:
- Dataframe and metadata are properly formatted for use with xpressplot

Parameters:
data: xpressplot-formatted dataframe, sample normalized (Required)
info: xpressplot formatted sample info dataframe (Required)
palette: Dictionary of matplotlib compatible colors for samples (Default: None)
grouping: What axis of the data to perform the analysis (default: 'samples' or columns; other options: 'genes', not yet implemented)
gene_list: List of genes to perform PCA across
gene_labels: Option for grouping='genes', not currently implemented

_3d_pca: Set to True to create 3-D PCA plotting principle components 1-3 (default: False)
principle_components: List of principle components to plot for 2-D PCA
n_components: Number of components to evaluate in the general analysis
ci: Confidence intervals to plot (i.e. 1 == CI1 == 68%, 2 == CI2 == 95%, 3 == CI3 == 99%)
scree_only: Only evaluate scree plot for n_components and exit
save_scree: Output scree plot to path and filename (automatically appends '_scree.pdf')
size: Marker size
order_legend: List of experiment groups in order to display on legend (Default: None)
title: Plot title (default: None)
fig_size: Figure size tuple; width, height (default: (16,6.5))
grid: Set to True to add gridlines (default: False)
whitegrid: Set to True to create white background in figure (default: Grey-scale)
save_fig: Full file path, name, and extension for file output (default: None)
dpi: Set DPI for figure output (default: 600)
bbox_inches: Matplotlib bbox_inches argument (default: 'tight'; useful for saving images and preventing text cut-off)
return_pca: Set as True to return dataframe with principle component values added
plotly_login: Include plotly login username and password to create an interactive plot, ex: ['username','password'] – not yet implemented

Notes:

- Exporting 3-D static PCA plots is not currently supported

Examples:

```
>>> xp.pca(geo_labeled, meta, geo_colors, grouping='samples', gene_list=None, gene_
↪labels=False,
    ci=2, principle_components=[1,2], n_components=10, _3d_pca=False, scree_
↪only=False,
    save_scree=None, size=10)
```

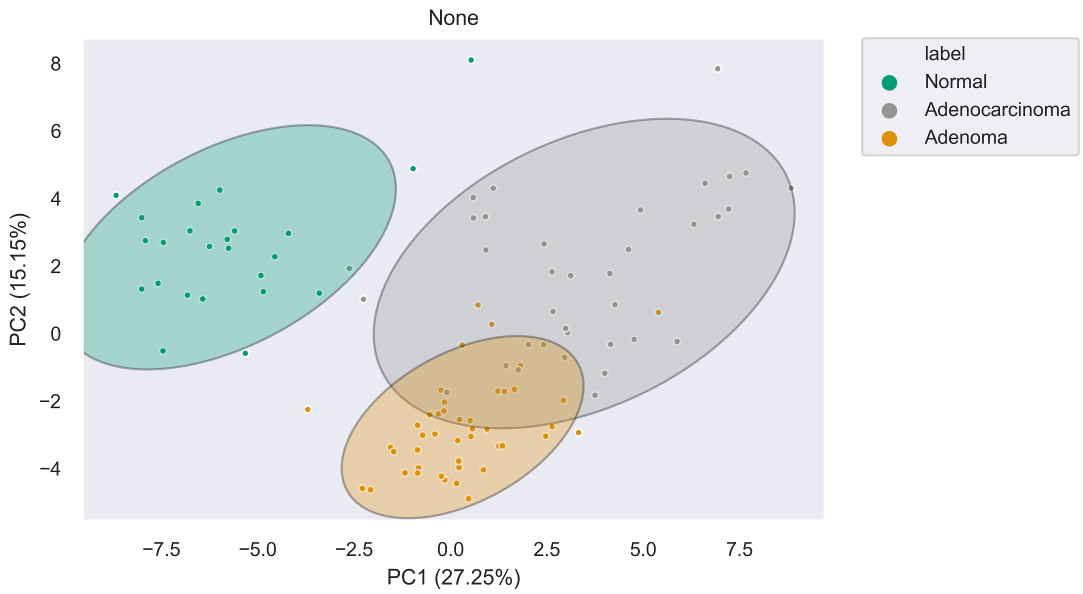

```
>>> xp.pca(geo_labeled, meta, geo_colors, _3d_pca=True, order_legend=[1,3,2], save_
↪fig=pca_file)
```

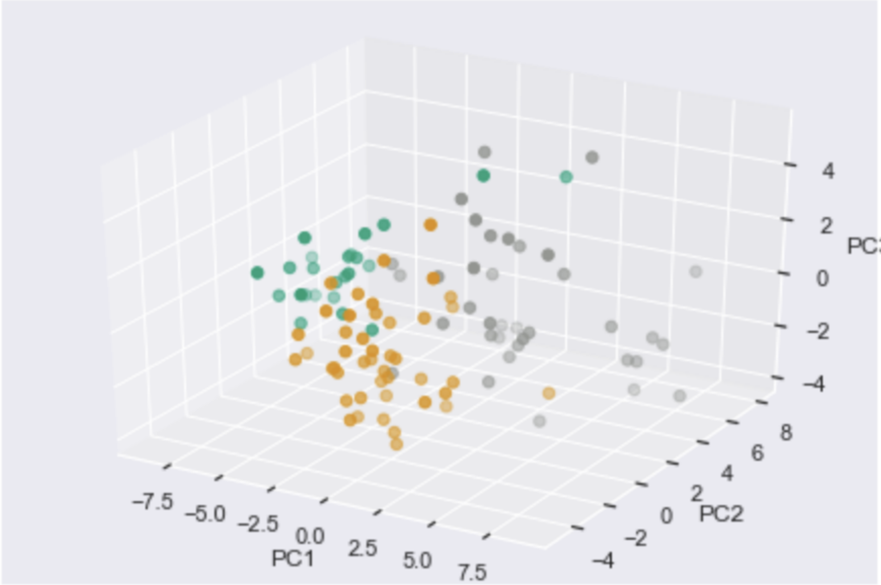


```
>>> xp.pca(geo_labeled, meta, geo_colors, _3d_pca=False, scree_only=True, save_
↪scree=True)
```

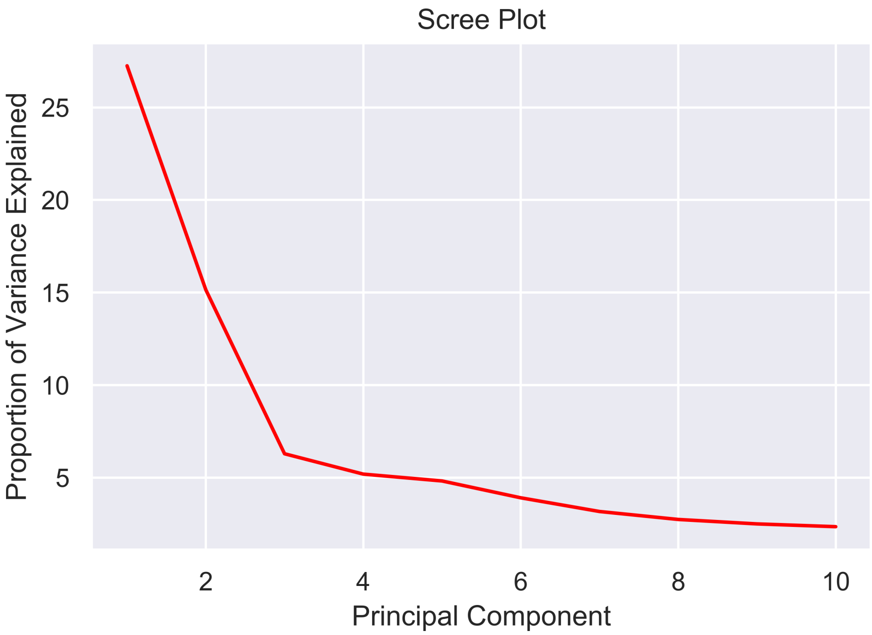

2.7 Updates

2.7.1 v0.2.2

- Fixes backend checks for matplotlib in tests and for HPC usage
- Updated library normalization equations
- Added administrative files

2.7.2 v0.2.1-beta

- Push manuscript submission version to PyPi

CHAPTER 3

License

XPRESSplot is freely available under a GNU General Public License (v3.0).

CHAPTER 4

Questions?

If you have questions, requests, or bugs to report, please use the Github issues forum.
