# Supplementary figures and images for: XPRESSyourself: Enhancing, standardizing, and automating ribosome profiling computational analyses yields improved insight into data
Source: PLoS Comput Biol. 2020 Jan 31;16(1):e1007625. doi: 10.1371/journal.pcbi.1007625 (PMC7015430; doi:10.1371/journal.pcbi.1007625)

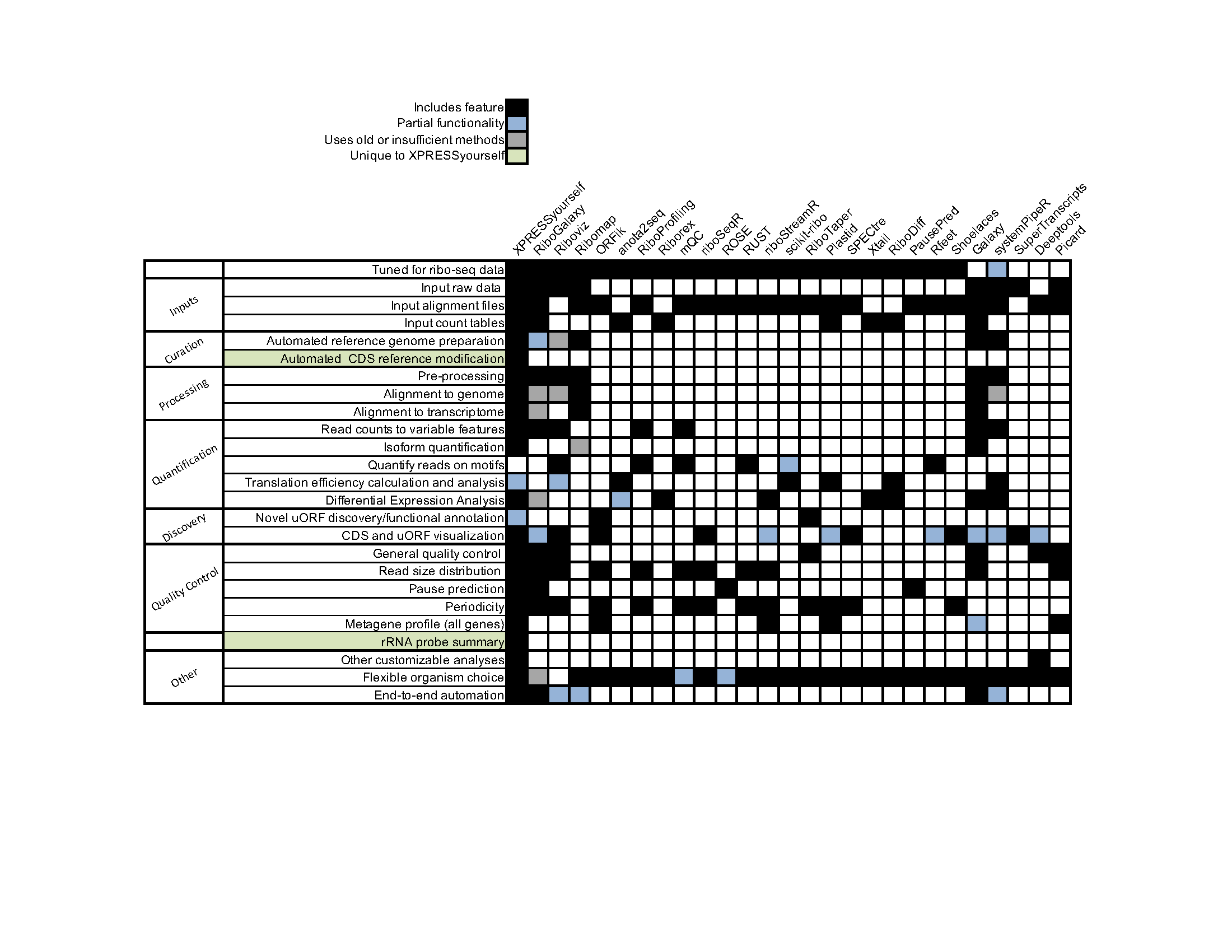

Supplement: S1 Fig — Black boxes indicate full functionality, blue boxes indicate partial functionality, grey boxes indicate incomplete or outdated functionality, and blank boxes indicate no functionality for the specified task. Rankings were compiled using the tools’ documentation, manuscript, and codebase. If a function was not clearly described in any of these venues, a blank box was given. (TIF) [file pcbi.1007625.s007.tif]

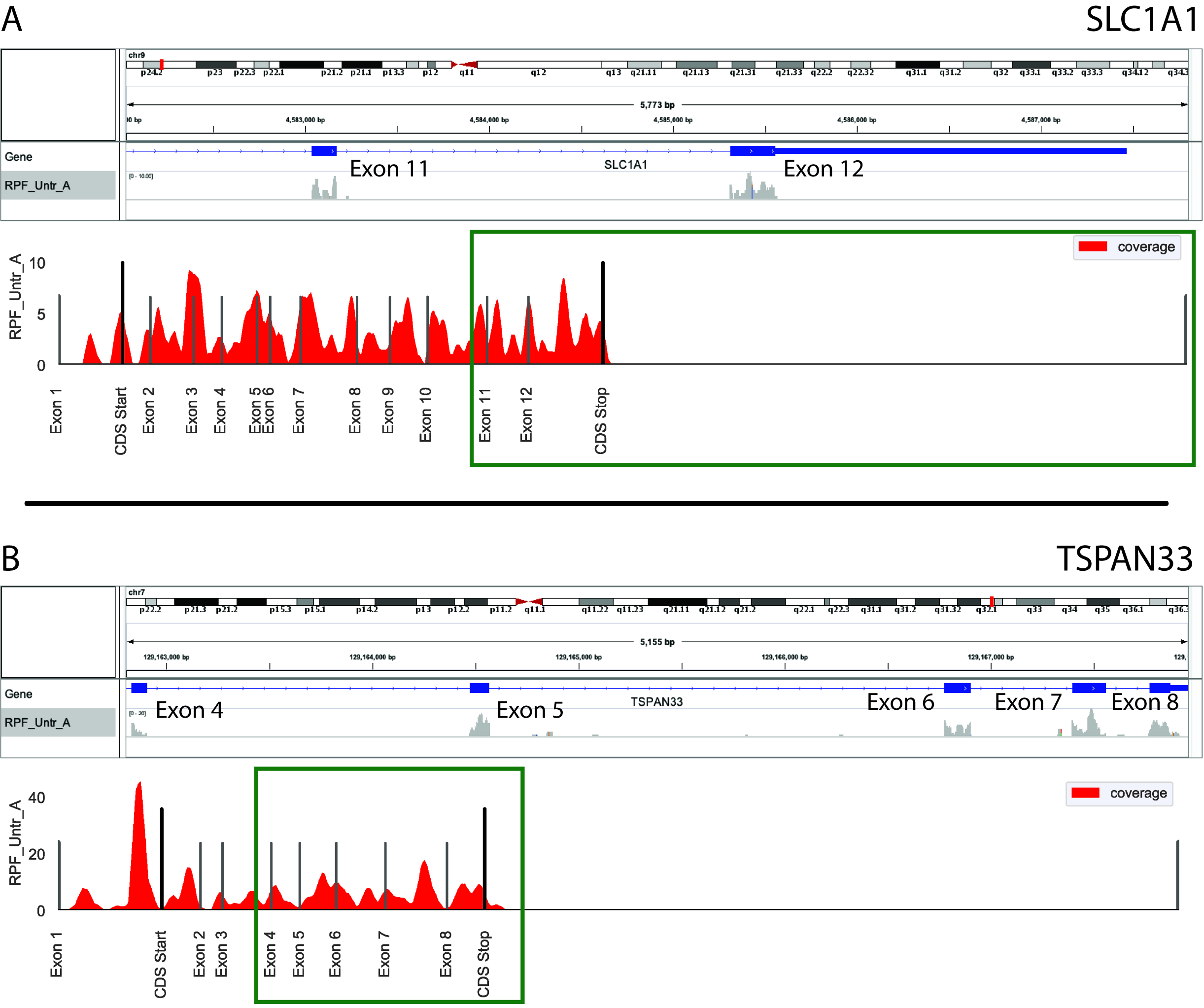

Supplement: S2 Fig — A) Gene coverage from IGV (above) and XPRESSpipe (below) for SLC1A1. B) Gene coverage from IGV (above) and XPRESSpipe (below) for TSPAN33. Introns collapsed by XPRESSpipe. Green box, region displayed in corresponding IGV window. (TIF) [file pcbi.1007625.s008.tif]

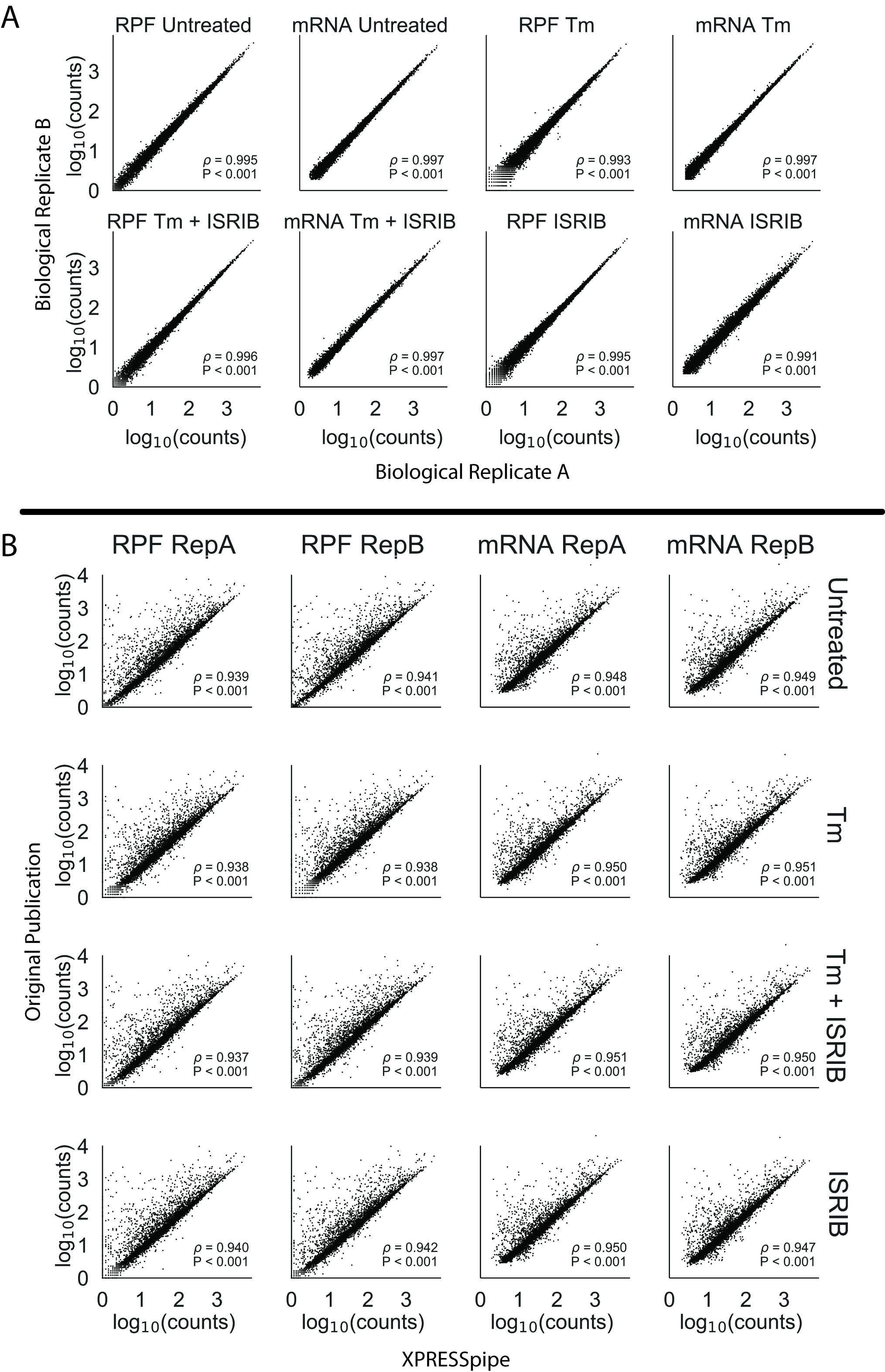

Supplement: S3 Fig — Genes were eliminated from analysis if any RNA-Seq sample for that gene had fewer than 10 counts. A) Comparison of biological replicate read counts processed by XPRESSpipe. B) Comparison of read counts per gene between count data from the original study and the same raw data processed and quantified by XPRESSpipe. RPF, ribosome-protected fragments. Tm, tunicamycin. All ρ values reported are Spearman correlation coefficients. XPRESSpipe-processed read alignments were quantified to Homo sapiens build CRCh38v98 using a protein-coding-only, truncated GTF. (TIF) [file pcbi.1007625.s009.tif]

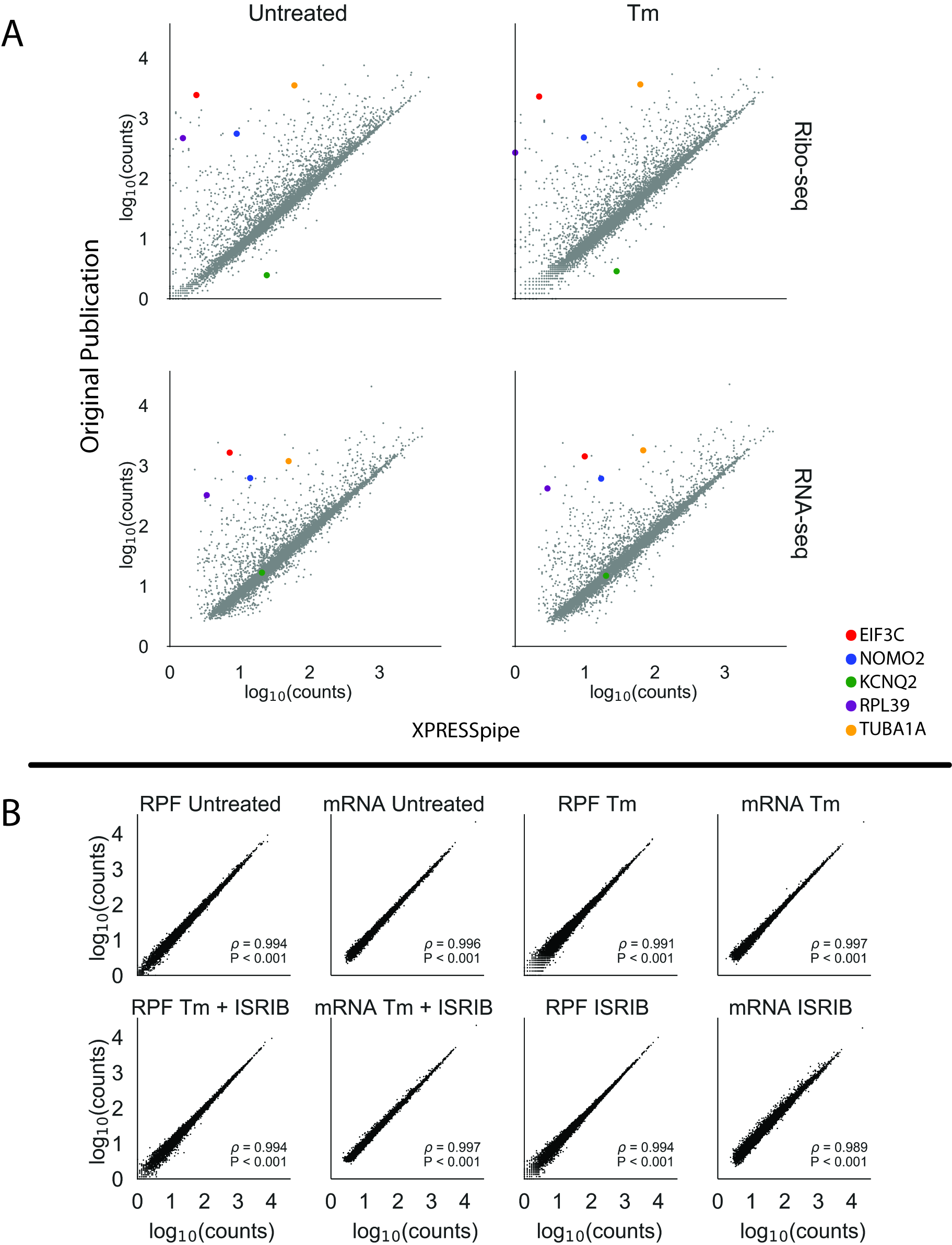

Supplement: S4 Fig — A) Selected highlighted genes show consistent differences between processing methods. B) Spearman correlation plots using the data table provided as supplementary data with the original ISRIB manuscript comparing biological replicates. RPF, ribosome-protected footprint. Tm, tunicamycin. All ρ values reported are Spearman correlation coefficients. (TIF) [file pcbi.1007625.s010.tif]

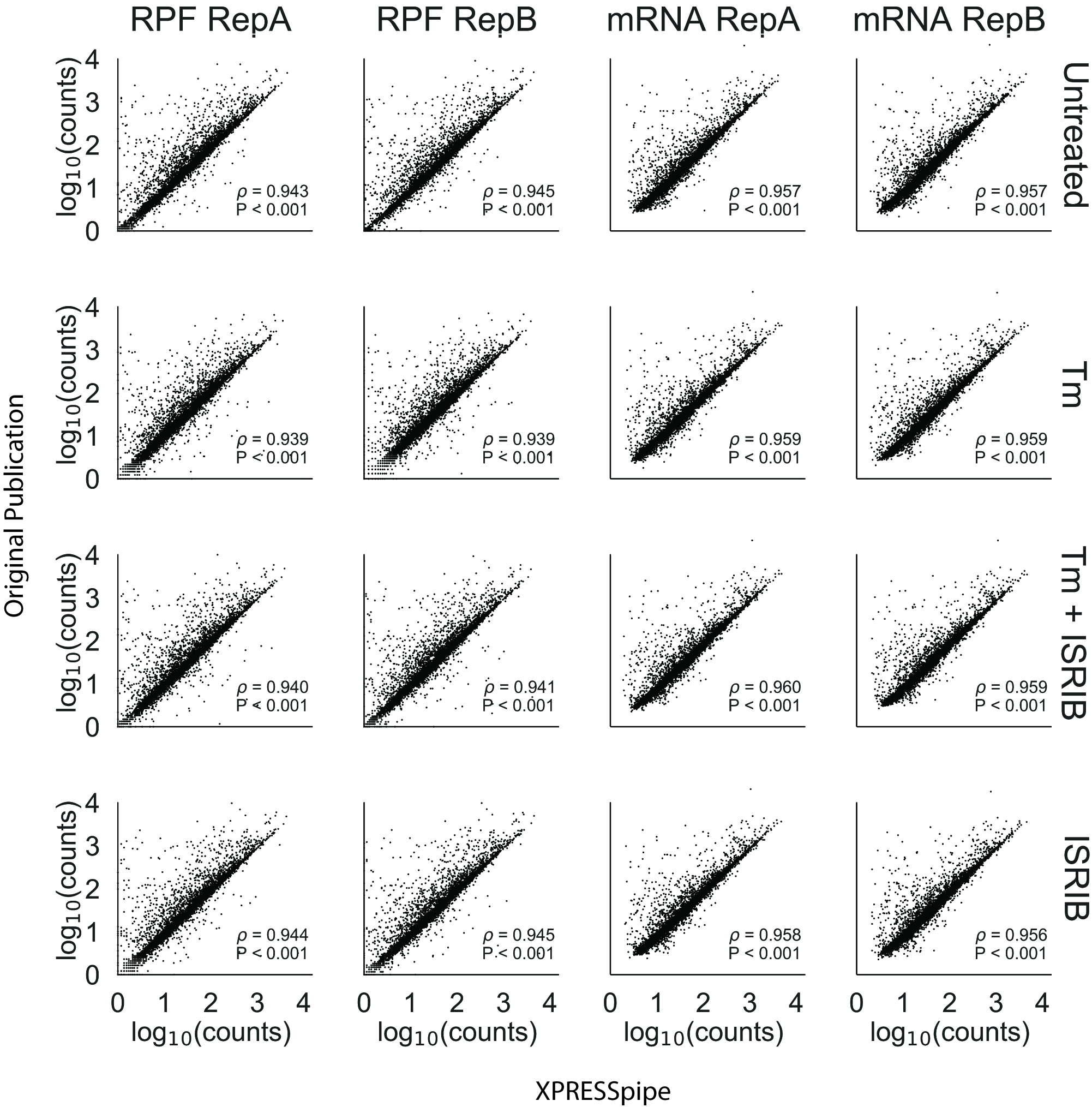

Supplement: S5 Fig — Original samples were processed using Ensembl human build GRCh38v72, as in the original manuscript, and compared with the original count data provided with the manuscript. XPRESSpipe-prepared counts were thresholded similarly as the original data (each gene needed to have at least 10 counts across all mRNA samples). RepA, biological replicate A. RepB, biological replicate B. RPF, ribosome-protected footprint. Tm, tunicamycin. All ρ values reported are Spearman correlation coefficients. (TIF) [file pcbi.1007625.s011.tif]

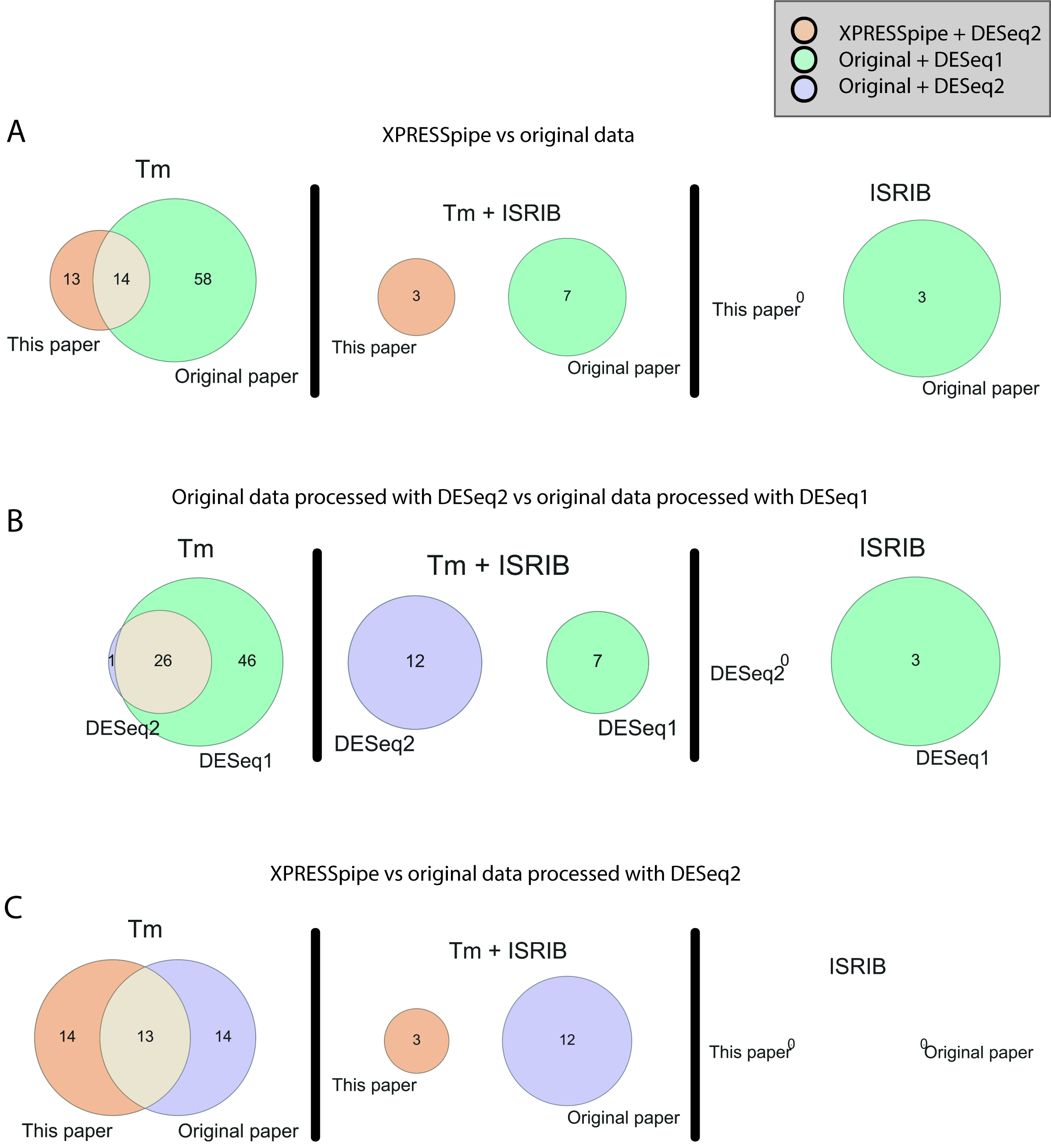

Supplement: S6 Fig — A) XPRESSpipe-processed data (orange) versus data as originally presented within original manuscript using original methods (green). B) Comparison of analyses using provided count table in original publication using DESeq2 (purple) versus original analysis provided in manuscript using DESeq1 (green). C) XPRESSpipe-processed (orange) versus originally-processed data (purple), both using DESeq2 for differential expression analysis. Light brown regions indicate overlap between gene lists. Thresholds used were the same as those used in the original study: |log2(Fold Change)| > 1, FDR < 0.1. (TIF) [file pcbi.1007625.s012.tif]

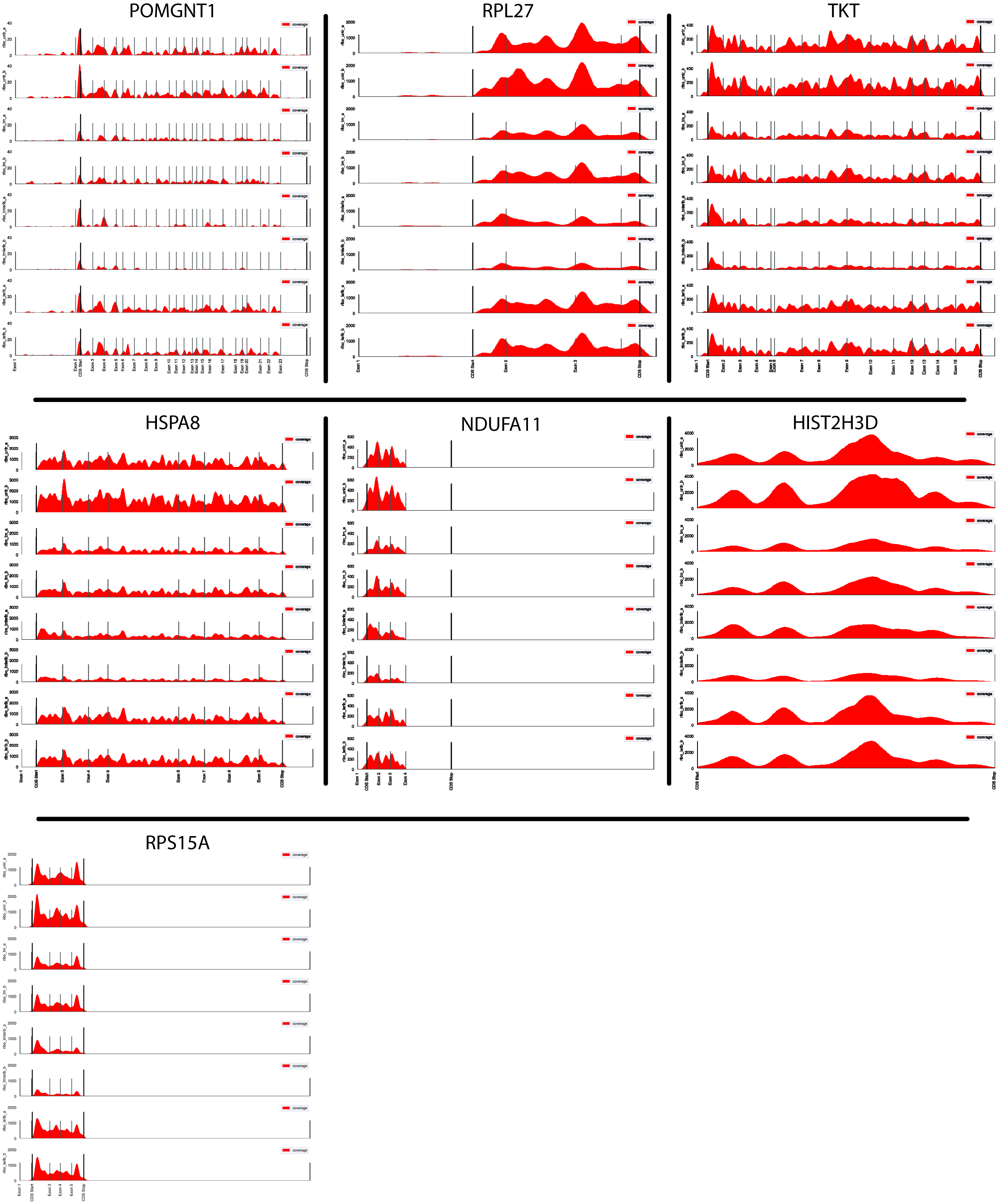

Supplement: S7 Fig — Coverage plots were generated using XPRESSpipe’s geneCoverage module, which collapses introns within the representation. (TIF) [file pcbi.1007625.s013.tif]
