# Supplementary material for: XPRESSyourself: Enhancing, standardizing, and automating ribosome profiling computational analyses yields improved insight into data
Source: PLoS Comput Biol. 2020 Jan 31;16(1):e1007625. doi: 10.1371/journal.pcbi.1007625 (PMC7015430; doi:10.1371/journal.pcbi.1007625)
Supplement: Attachment — Submitted filename: xpressyourself_plos-cb_revision_summary.pdf [file pcbi.1007625.s014.pdf]

We thank the reviewers for their thoughtful comments and suggestions. We have considered these points, and feel these revisions dramatically improve the clarity and presentation of the manuscript. We have addressed each comment below in green italic text.

Reviewer #1: I do not have extensive experience in the area of ribosomal profiling data analysis, but have reviewed the manuscript with respect to the bioinformatic tool description, analyses, and the accompanying software. The manuscript is carefully written, and it provides a useful pipeline for the uniform processing of ribosomal profiling data with best practices in mind. The software should be of utility to the community of bioinformatic analysts. The software is well documented at https://xpresspipe.readthedocs.io and https://xpressplot.readthedocs.io, and the GitHub repository for the paper provides all of the code used to produce the analyses in the paper.

I have the following minor comments:

* There are some ribosomal profiling software packages existing, although they probably handle some subset of the full pipeline. Michel [5] and Carja [8] are cited, but there appear to be at least a few more from searching with Google, e.g.,

https://doi.org/10.1186%2Fs12864-018-4912-6
https://doi.org/10.1016/j.cmpb.2018.10.018

And a few in the Bioconductor project are listed here

https://bioconductor.org/packages/release/BiocViews.html#___RiboSeq

I was expecting a table describing these, and e.g. a grid of checkboxes with respect to supported features compared to XPRESSpipe. Presumably this would also help to convince users to use XPRESSpipe as it would cover a broader range of the pipeline, and also indicate that some of these may now not be using best practices.

We have added the requested table as a supplement and reference it and the relevant citations in the introduction, where we discuss the current state of ribosome profiling computational analysis. We agree that the addition of this table adds clarity to the functionality of XPRESSyourself.

* The intron-removal for the purposes of visualization reminds me of this paper, which could be cited if appropriate:

https://genomebiology.biomedcentral.com/articles/10.1186/s13059-017-1284-1

We thank the reviewer for directing us to this reference. We have included a brief discussion and comparison of this tool, superTranscripts, with XPRESSyourself under the "Gene Coverage Profile" section of the manuscript. superTranscripts can perform the same tasks as the geneCoverage sub-module in XPRESSpipe; however, XPRESSpipe performs these tasks in an automated manner and does not require the user to perform additional steps within an external tool, such as IGV. Additionally, during its implementation within XPRESSpipe, geneCoverage is automatically run on housekeeping genes so that the user can quickly visualize representative coverage across control genes. Other tools designed for ribosome profiling can also perform similar tasks to some extent, but again, not in the automated and robust manner provided by XPRESSpipe, and often without the appropriate annotations to aid the user in identifying interesting upstream translation events.

* There appear to be a lot of noisy log2 fold changes in Figures 3A-D. Can these be fixed by filtering of low

count features or use of pseudocounts in calculating the ratio, or the shrinkage techniques in the DESeq2 software that is used in calculating the TE?

We agree that the referenced figures contain a small number of noisy log_2(fold changes) that seem to aberrantly fall perfectly on horizontal lines. These data represent genes that pass RNA-seq thresholding but contain low or no RPF counts, which result in null FDR values for statistical analyses of translational efficiency. We highlight some of these cases in the code associated with the manuscript figures. Because some of the genes highlighted in the original study have a very low translational efficiency in one-or-more conditions, we did not want to impose a threshold nor a pseudocount on RPF values. As an alternative, we raised the threshold for RNA-seq data from 10 counts to 25 counts. We chose to threshold at 25 counts across RNA-seq samples and not a higher value in order to maintain ATF5 in the dataset, which was emphasized in the original study that generated this data (one RNA-seq sample only had 29 counts in our processing). Overall, this adjusted expression cut-off removed most of the noisy data points while keeping those few protein-coding genes that are indeed robustly expressed but which produce no RPFs. The relevant figures have been updated, and the list of putative neurodegenerative targets has been updated due to the resulting changes in FDR values.

* The software appears to be well described and documented, and the online documentation points to the GitHub Issues for user support. Consider also mentioning how users can obtain support in the manuscript. Additionally, the authors could provide information about how users would find out about changes to the software over time (e.g. as best practices evolve, where should users look to find descriptions of changes since the initial publication?).

We have updated the documentation to provide version-update summaries. In the ReadTheDocs pages, this can be found under the "Updates" tab. In the README files on each tool's landing page, links to the GitHub "Versions" tab are provided. These additions have also been referenced in the manuscript under the "Architecture and Organization" sub-heading.

Reviewer #2: The Manuscript describes XPRESSyourself, a comprehensive software package for analyzing the ribosome profiling data. As the Authors correctly point out, this experimental technique is gaining popularity, and at the same time, there is a lack of robust software tools to deal with idiosyncrasies if this data type. The XPRESSyourself tool appears to be expertly designed, with thoughtful considerations given to the accuracy, efficiency, robustness, and user-friendliness. The manuscript is well written and presents a biologically interesting story that arose from the re-analysis of a previously published dataset. I have several suggestions that I believe could improve the paper.

1. Figure 1. It would be helpful to indicate directly in Figure 1 (in addition to the description in the body of the text) which tools and/or custom scripts are used for each of the steps, and which choices are available for the users. Also, specifying the format of the output files at each step (BED, BAM, txt, etc) would be useful for bioinformaticians who are willing to design their own pipeline extensions.

The requested information has been included in the updated Figure 1. The legend has likewise been updated to describe these additions.

2. Figure 2. Presenting multiple scatter plots that all look pretty much the same seems to be wasteful of space. I would recommend presenting just one plot in (A) and (B) each, and moving all others to Supplementary materials. Instead, I would show the box-plots for the Spearman R between replicates and between methods (B). In addition to Spearman R, I would also recommend checking Pearson correlation of log(count+1), which sometimes gives a different picture.

We now show two plots for both 2A and 2B (the worst representative correlation for a ribo-seq sample and an RNA-seq sample) for each comparison type (internal replicate comparisons and cross-method comparisons).

We have moved the rest of the original figure to a supplemental figure. We also included panels C and D to Figure 2 with the suggested boxplots with a violin plot to show distributions of data points for both Spearman and Pearson correlations. By both Pearson and Spearman, replicates and between-method comparisons are well correlated (some differences are seen between methods, which we discussed in the manuscript, likely due to the updated methodologies used in XPRESSpipe).

3. In addition to correlation coefficients, it would instructive to compare the lists of significantly differentially expressed and TE genes yielded by the old and the current method. It can be presented as a Venn diagram. What are the genes with a significant TE increase in Tm treatment that were identified with XPRESSyourself but not with the old method?

We have included the requested Venn diagrams comparing the differentially expressed significant hits from the original study and the same data processed by XPRESSpipe as a supplemental figure. We feel that their addition aids in depicting the magnitude of differences we observed between the two methods. We find that our processing returned fewer "strong" differentially expressed genes for each condition. It is likely this stems from the use of alignment (STAR vs. Tophat2) and/or differential expression (DESeq2 vs. DESeq1) software(s). However, as pointed out in other studies, some ribosome profiling analyses are not fully transparent in their methodology, so it is difficult to precisely determine the cause of these differences. We did, however, re-analyze the count data included with the original study using DESeq2 and find the results much more in line with those produced by XPRESSpipe. We believe that about half of the differences we see with our processing compared to the original stems from alignment differences, and the remainder from the usage of DESeq2 over DESeq1.

4. I would recommend that the discussion about computational costs and requirements is moved to the main text, as it is important for many users. Also, it would be interesting to break the overall run-time and RAM requirements into separate values for each of the tools/modules.

We have moved this information to the main text as requested. Sub-module statistics for processing time and memory requirements are now provided within the original table provided for this section.

5. "...systematic biological artifact…" If it's biological, it's not an artifact

We have changed the wording to "systematic biological signal" in the manuscript, while still making clear that this signal is not thought to reflect gene-to-gene differences in translational efficiency.

Reviewer #3: The authors suggest a toolkit (XPRESSyourself) for better analyzing ribo-seq (which is mainly based on various previous software/tools) and demonstrate it via the analysis of publicly available ribosome profiling data related to neurodegenerative phenotypes. As I describe below my impression is that the tool does not include novel enough aspects to be published in a journals such PLoS-CB. Nevertheless, I provide below some suggestions for improvements.

Major
------

1) They provide a code and not a GUI; thus a biologist with no programming experience can use it.
This means, that all the researchers that can use this code have programming expertise and thus can easily write all the simple analyses they provide or call the functions/tools that they use (since this is very easy to do I guess that this is what the potential users will typically prefer to do). Thus, eventually it is not clear to me who will use this tool.

We acknowledge that the lack of a GUI could be challenging or intimidating for some without programming experience. We therefore went to great lengths to make overcoming these challenges as easy as possible for as many users as possible. For example, all functionality is thoroughly explained with documentation and examples at xpresspipe.readthedocs.io and xpressplot.readthedocs.io. In addition, we supply instructional walkthrough videos and Jupyter notebooks that walk the user through the analysis step-by-step. We also include in the documentation a walkthrough on how to interface with compute clusters and run XPRESSpipe, which is the environment we imagine most using this tool due to the computational requirements of these tools. Although the walkthrough videos are limited to single, standard examples for XPRESSpipe, we have additional resources, accessible by executing "xpresspipe build" in the command line (instructions are referenced in the README), which walk the user through the customization choices that may be necessary for processing and analysis of their specific data. In all, we have attempted to limit the amount of coding the user would need to perform de novo. To this end, the entire processing and analyzing of a standard ribosome profiling dataset can be performed in two to three simple commands. In addition, we had individuals with no programming experience test the software and they responded that they were able to process their data with minimal difficultly.

We hope that the primary contribution of this work is to serve as an open-source software pipeline that allows users of all computational skill levels to implement rigorous and reproducible ribosome-profiling analysis with current best practices, which has to date eluded many wishing to use this sequencing method. By creating a resource such as XPRESSyourself, users are saved from having to rewrite functionality, both trivial and non-trivial. We will explain some more complex components that XPRESSyourself addresses in more detail in response to a later question below. Additionally, learning the ins and outs of processing sequence data, especially ribosome profiling data, is not a trivial task and we have gone to lengths in the documentation to describe the choices a user must make in data processing to automate the procedures for the user, while also helping make sure they are not just blindly allowing a black box to process their data. We hope that XPRESSyourself will be both an enriching experience, as well as minimize one of the primary bottlenecks in data analysis.

2) There are many tools for analyzing ribo-seq (in addition to the one cited see e.g. PMID: 30049792,https://www.biorxiv.org/content/10.1101/106922v1.full, PMID: 27347386, PMID: 28158331, PMID: 30401579 .. this is a very partial list you should search for more additional tools). I do not find a clear explanation re. why we need an additional tool (you should clearly explain what are the *non trivial* aspects that you provide ?).

We thank the reviewer for pointing out these additional references, and we have added these along with others to the software comparison table that was added in response to a comment above. This table summarizes the functions of these various tools. We feel this table clearly demonstrates how XPRESSyourself combines and improves on these tools.

As discussed in response to the previous comment, XPRESSpipe acts as a central reference for best practices of ribosome profiling data processing. Although there are pre-existing tools for ribosome-profiling analysis, these tools perform a limited number and inconsistent set of quality control tasks and other analyses and do not provide end-to-end functionality. For example, one such reference provided above presents anota2seq for performing differential expression analysis. This tool has certain limitations, such as requiring three or more replicates per experimental condition. Most studies will only generate two biological replicates per condition at most due to the time and cost of preparing ribosome profiling samples, as was done in the reference study used in our manuscript (DOI: 10.7554/eLife.05033).

Despite the extant tools for analyzing ribosome-profiling data, it is clear that there is no open-source pipeline currently available that leaders in the ribosome-profiling field are comfortable using. Many of these tools are outdated, inaccessible, unreliable, or only perform very specific analytical tasks. We believe XPRESSyourself fills the need of an open-source pipeline that can go from raw sequencing to analyzed and visualized data with minimal user input, and fills it not only for labs with experience working with ribosome-profiling data but more importantly for new users that may not have extant, lab-specific tools to fall back on.

Additionally, we have clarified additional non-trivial aspects of our work in our revised manuscript. For example, we more explicitly discuss the fundamental benefit of having a uniform standard built upon current best practices for analyzing this specialized data type. As discussed above, for a wet-lab user of ribosome profiling, learning the intricacies and considerations of processing sequence data can be difficult and time-consuming to do correctly, particular with the idiosyncrasies specific to ribosome profiling. We provide extensive documentation on considerations being made at each step with these data. So, besides knowing they are using best practices for analyzing their ribosome-profiling data, they are also better able to understand why each step of the pipeline is implemented the way it is. As we will discuss more below in response to another comment of this reviewer, writing a robust, recursive script to process CDS truncation, a necessary step in ribosome profiling analysis, is not a trivial task, especially for those without any computational background. To our knowledge, no tool is publicly available for this task, particularly when genomic alignment is preferred or required over transcriptomic alignment. Other useful tools, such as our rRNA probe generator, offer the ability to identify the most problematic rRNA fragments across sequence libraries. This tool is gaining more relevance as ribosome profiling moves into new organisms where commercial kits may be ineffective at subtractive hybridization of rRNA fragments as produced during the RNase 1 digestion step of ribosome profiling. In fact, even with standard model organisms, commercial kits are often insufficient for this task. If not specifically removed, a few fragments of rRNA can easily make up more than 90% of a sequencing library. Other tools, such as the metagene and gene coverage profilers, again offer tools where few similar tools exist and offer a fully integrated, automated, and ribosome profiling-tuned option.

As a whole, we hope it is clear that developing simple scripts for these tasks is not possible and require more advanced programming knowledge than that possessed by many wet-lab scientists and trainees. Thus, XPRESSyourself should greatly reduce the bottleneck in analyzing these data for the typical user.

3) " For example, the pile-up of ribosomes at the 5'- and 3'- ..."
The authors give a long example re. why their tool is needed: it is the first to deal with the bias at the coding sequence ends. However their solution is trivial -- cutting/ignoring the ends of the coding sequence; any user that can use their code can *very* easily write a code for ignoring the ends.

Some solutions to this problem may indeed be more trivial than others. For example, once a user has a transcriptome-aligned BAM file, they could load a GTF file into R and use the GenomicFeatures package to flatten and modify the CDS start and end sites, then use this annotation object within R. However, in our trials with this solution, these modified objects cannot be back converted into a correctly formatted GTF file. Another solution could arise using the GenomicAlignments package in R, where the user could conditionally remove alignments from a transcriptome-aligned BAM file falling in these 5' and 3' ends; however, proper implementation would require consideration of edge cases and correct position cut-offs. Additionally, this method does not indicate where the CDS starts or ends for a transcript, further limiting its use out of the box. Both of these solutions limit the user to using only transcriptome-aligned files. We have included a short script in the manuscript repository where these methods were explored. Additionally, this solution allows for limited downstream functionality, as we will describe in more detail below, and even implementation of this particular solution could be challenging for a beginning programmer.

In order to build a more broadly applicable solution to truncating CDSs, we modify the GTF file directly to preserve original file formatting. However, in genomic space, many CDSs are interrupted by an intron well before the point of truncation is reached (45 nt). In fact, many CDSs are interrupted by more than one intron before reaching the point of truncation. In all, this problem is applicable for 2-15% of all transcripts across model organisms (see table below, now included in the supplement of our manuscript). We determined that a recursive strategy was necessary to treat each CDS equally and remove the same number of nucleotides from each before mapping ribosome-protected fragments and RNA-seq. Would this task be trivial for a trained bioinformatician or computational biologist? Likely yes, as could be considered the case with most computational biology tools, though it might take non-trivial time to accurately implement and test. Again, we believe it is preferable to have such a tool publicly available and not require it to be rebuilt by every lab that wants to analyze ribosome-profiling data using best practices. Would this task be trivial for most users of XPRESSyourself? No. This scenario emphasizes the utility of XPRESSyourself, which is to provide a rigorous and reproducible implementation of all processing tasks required for correct analysis and interpretation of ribosome-profiling data.

We feel strongly that this approach is the best one as this allows for genomic coordinate-aligned BAM file output and downstream use with the largest swath of downstream methods. Particularly, it allows for quantification with software such as HTSeq and featureCounts, and downstream analysis with DESeq2. This also allows for quantification with methods such as Cufflinks for transcriptome abundance calculation. Aside from this, it is generally accepted that straight transcriptome alignments underperform compared to genomic alignment methods. While some are willing to accept these different margins between methods, we feel this sacrifice would be inappropriate for this reference implementation summarizing best methods.

Statistics in the table below were compiled using Ensembl v97 in most cases, with the exception of Arabidopsis thaliana, v44 (recent version with different versioning). Statistics are the number of transcripts where the 5' end requires recursive truncation. Emphasizing this need, numerous Ensembl canonical transcripts only contain 3-6 nucleotides in the first coding exon before an intron in genomic space. Therefore, the lack of recursive trimming of the first 45 nt would lead to unequal trimming of coding sequences and lead to drastic biases in counting between genes based on intron position relative to start codon. This table is now included in the methods section of the manuscript.

	# CDSs requiring recursive truncation	*# of transcripts total in organism*	*Percentage of recursive CDSs to total transcripts*
Arabidopsis thaliana	*4,428*	*54,013*	*8.20%*
Caenorhabditis elegans	*5,048*	*61,451*	*8.21%*
Danio rerio	*8,987*	*59,876*	*15.01%*
Drosophila melanogaster	*4,262*	*34,793*	*12.25%*
Homo sapiens	*21,718*	*226,788*	*9.58%*
Mus musculus	*12,779*	*142,333*	*8.98%*
Rattus norvegicus	*5,002*	*41,078*	*12.18%*
Saccharomyces cerevisiae	*160*	*7,127*	*2.24%*
Xenopus tropicalis	*3,854*	*24,197*	*15.93%*

I thought that they could give a novel/sophisticated solution that will deal with the fact that these regions do also include important signals (e.g. PMID: 25505165) that we may want to specifically study.. or suggest what is the exact region to remove to decrease bias but not removing important signals. Without such a solution their suggested feature is not helpful.

A note referencing this consideration for ribosome profiling data and the associated reference have been included in the "GTF Modification" section of the manuscript.

It is the standard in the field to ignore ~45-50 nt from the 5' end and ~5-15 nt from the 3' end for quantification, and we therefore use this standard for our pipeline (see DOI: 1016/j.celrep/2016.01.043 and 10.1016/j.ymeth.2017.05.028). By default, XPRESSpipe truncates 45 nt from the 5' end and 15 nt from the 3' end of each transcript. While it is true that these 5' and 3' regions include biologically important initiation and termination signals, standard ribosome-footprint profiling is generally not the appropriate experimental method to dissect these signals. However, if a user wished to include these regions in their analysis, the pipeline can easily be run without a truncated reference. Additionally, users can specify to quantify translation in the untranslated regions of genes, or anywhere else in the genome if they so choose.

4) There are additional previous papers on ribo-seq biases/challenges that should be cited/mentioned (PMID: 27638886, PMID: 27160013).

We have included the suggested references, along with several others, to the introduction of the manuscript.

5) The biological example they provide (in the section " Benchmarking Against Published Ribosome Profiling Data and New Insights ") is not very convincing: first, I guess that the improvement is mainly due to the usage of a new alignment tool (STAR); thus, this is mainly an advertisement to STAR.

A main point of this manuscript, in our view, is that by providing the user with the most current and well-vetted methods for use under the hood in XPRESSpipe, analysis will be more rigorous and reliable. Therefore, we agree that our study demonstrates some benefits of STAR compared to predecessors, but more in the sense that the STAR algorithm is superior to its predecessors, as verified by benchmarking studies, and will thus yield more reliable results. We additionally now show the other improvements in analysis can be accounted for from the use of DESeq2 over its predecessor. Additionally, in this manuscript we wanted to emphasize the utility of revisiting old datasets to optimize the yield of information the scientific community can obtain from publicly available data using improved analytical—and in this case, user-friendly—methods. We have revised the text in the manuscript to clarify this point.

Second, most of the results are similar in the old and new analyses.

In response to another reviewer's suggestion, we provided a Venn diagram to more systematically detail the differences and similarities between our analysis and the analysis provided in the original manuscript.

Although several of the results we highlight mirror those of the original manuscript, this was intentionally done to show future users that this pipeline has been vetted and can produce reliable results. We chose to analyze the ISR/ISRIB dataset because the canonical post-transcriptional regulation of ATF4 and other genes during integrated stress response (ISR) has been studied for decades and is well understood outside of the context of high-throughput sequencing. However, as the original study used now outdated methodologies in their computational analysis, we hoped to highlight some of the results and insights one might obtain from the re-analysis of such a dataset today. ISRIB is a particularly interesting case because much effort has been expended in recent years to test this small molecule's role in neuroprotection. For this reason, we decided to examine genes that are translationally down-regulated during the ISR without ISRIB treatment, but which have significantly less translational downregulation with ISRIB treatment. Genes fitting this criterion may further explain ISRIB's neuroprotective capabilities. These patterns were not considered in the original study and are unique to our analysis.

Third, they suggest that they found relevant down regulated genes that the old paper did not find; however, the comparison is not performed in a systematic manner: you should compare all the genes you found and the old paper found and give some general quantitative measure (over *all* of them) that shows that overall your are better (e.g. has the old paper found relevant genes that you haven't found? do you find false-positive -- genes that seem to be differentially translated but not seem to be relevant?).

Unfortunately, without a ground truth-validated dataset, we have no way of comparing false-positive vs true positive rates. Eventually, these targets could be experimentally validated as we point to in our discussion within the manuscript, but we feel such experiments would be outside the scope of this paper. We remain interested in following up on these putative targets in the future. However, as best we can, we now outline what measures we employed to reduce false-positives and provide a more systematic comparison across methods, in addition to the Venn diagrams that were added as a supplementary figure in response to a reviewer's suggestion.

During our differential expression analysis, we set the Benjamini-Hochberg FDR α to 0.1 to reduce false-positives. As changes in translation efficiency (TE) were the primary metric we used to develop our list of putative gene targets, we can say that the likelihood that the TE of genes down-regulated during Tm treatment is a false-positive hit after p-value adjustment ranges from 1.12e-05 to 1.31e-16. Looking at TE recovery during Tm + ISRIB treatment, all except POMGNT1 had an FDR <= 8.69e-04, indicating that these genes showed significant upregulation (>= 1.4 fold change) compared to WT TE levels; thus our confidence in saying this target was downregulated during Tm treatment and recovered to WT levels during Tm + ISRIB treatment is relatively high. For the remainder of the putative targets, the likelihood that their TE recovery increase over WT levels falls within a false-positive range of 8.69e-4 to 1e-6. Therefore, we feel we can say with high confidence that these changes are statistically significant and that they would be reasonable targets to follow-up to confirm their role during ISRIB treatment and whether they play any role in the neuroprotective capabilities of ISRIB. However, aside from comparing the genes themselves between our analysis and that of the original publication, making a systematic comparison of which processing method is correct is difficult without a ground truth, until otherwise validated. We hope that the included Venn diagrams will at least address this partially.

Furthermore, based on a recent publication exploring "dataset decay", or the increase in false-positive rate with subsequent analyses of the same dataset (https://doi.org/10.1101/801696), we further explored the reliability of these hits. Based on a search of publications citing the study we re-analyzed, there are 199 citations to date (Google Scholar, 26 Oct 2019). By sampling 20 of these publications, we see that ~10% have re-analyzed the data in some fashion. Based on the experimental α-debt dataset decay method, this decreases our α to approximately 4.77E-08. For the Tm treated cells, this only causes the FDR of POMGNT1 during Tm treatment to drop below the α-threshold.

It is not possible to perform the exact analysis with the original data due to the lack of exact details on how it was originally performed. We can, however, perform an approximate systematic comparison of the results for neuroprotective gene targets between the methods. Using our pattern-identification methodology, we find 5/7 (71.4%) of our hits with neural functions, and the same 5/7 (71.4%) have annotated severe neurological defects resulting from perturbations in the gene. The original study identified seventeen genes with a similar pattern, with 12/17 (70.6%) possessing some neural function annotation, and only 4/17 (23.5%) are associated with some type of neurological defect. We want to point out that our targets' neurological defects often presented as cerebral hypoxia or brain atrophy. The majority are tied together through the commonality of metabolic insufficiencies. For the targets arising from the original study, two were associated with epilepsy, one with neural tube defects, and one shown to be able to help brain function when overexpressed. Based on these comparisons, along with the use of well-vetted software we use in each step of the pipeline (as discussed and cited throughout the manuscript), we feel the targets obtained from XPRESSpipe processed data are interesting based on their constrained function and disease outcome and worth further follow-up.

To further emphasize this point, we processed the original publication's count data using DESeq2 (instead of the now deprecated DESeq1 as used in the original publication) and find thirteen genes that fit our pattern-identification methodology from this data. Four of the genes we identified with strong neurological phenotypes are now present by way of this re-processing. More details on this comparison can be found in the supplemental Venn diagrams and in the updated Results section.

6) One challenge in the field is dealing with various splicing isoform in the case of ribo-seq. Do you have a solution for this while considering *all* the relevant isoforms ?

We agree that this is a challenge in the field. Unfortunately, it is a challenge where solutions are necessarily limited by the short length of ribosome-protected fragments and therefore the limited ability to precisely identify what splice isoform each fragment is derived from. In theory, the accompanying bulk RNA-seq libraries should likewise be fragmented to similar sizes as the footprints to control for technical sequencing biases. Although software to handle this challenge of isoform abundance of ribosome footprints is limited, one potential solution is Ribomap (DOI: 10.1093/bioinformatics/btw085). This package couples read quantification with mRNA transcript abundance calculated by an alignment program like Cufflinks (DOI: 10.1038/nprot.2012.016). It appears that, based on their benchmarking and validation, this method offers a reasonable solution to this challenge, even if just one of the early iterations of a solution. However, it is difficult to perform a full benchmarking validation as there are no other similar solutions currently available to our knowledge. Additionally, this methodology assumes the user's bulk RNA-seq libraries read length distributions are significantly longer than 17-33 nt distribution expected from ribosome footprints. As discussed above, this approach could lead to undesired biases during sequencing. It will be interesting to see more development on this challenging alignment problem in the future; however, we will not be including this tool in a toolkit summarizing best-practices until further benchmarking validation has been performed.

7) Please increase the fonts in figure 2.

We have increased the figure font sizes as requested.
